# Global prevalence of *Ascaris* infection in humans (2010–2021): a systematic review and meta-analysis

**DOI:** 10.1186/s40249-022-01038-z

**Published:** 2022-11-18

**Authors:** Celia Holland, Mahdi Sepidarkish, Gwendoline Deslyper, Ali Abdollahi, Soghra Valizadeh, Abolfazl Mollalo, Sanaz Mahjour, Sahar Ghodsian, Ali Ardekani, Hamed Behniafar, Robin B. Gasser, Ali Rostami

**Affiliations:** 1 Department of Zoology, School of Natural Sciences, Trinity College Dublin, College Green, Dublin 2, DO2PN40 Ireland; 2 Department of Biostatistics and Epidemiology, School of Public Health, Babol University of Medical Sciences, Babol, Iran; 3 Department of Surgery, Faculty of Medicine, Tehran Medical Sciences, Islamic Azad University, Tehran, Iran; 4 University of Mohaghegh Ardabili, Ardabil, Iran; 5 Department of Public Health and Prevention Science, School of Health Sciences, Baldwin Wallace University, Berea, OH USA; 6 Department of Psychiatry and Behavioral Sciences, Northwestern Feinberg School of Medicine, Chicago, IL 60611 USA; 7 Department of Parasitology and Mycology, School of Medicine, Shahid Beheshti University of Medical Sciences, Tehran, Iran; 8 Health Policy Research Center, Institute of Health, Shiraz University of Medical Sciences, Shiraz, Iran; 9 Department of Medical Parasitology, Sarab Faculty of Medical Sciences, Sarab, East Azerbaijan Iran; 10 Department of Veterinary Biosciences, Melbourne Veterinary School, Faculty of Veterinary and Agricultural Sciences, The University of Melbourne, Parkville, VIC Australia; 11 Infectious Diseases and Tropical Medicine Research Center, Health Research Institute, Babol University of Medical Sciences, Babol, Iran

**Keywords:** *Ascaris*, Global prevalence, Intensity of infection, Systematic review, Meta-analysis

## Abstract

**Background:**

Ascariasis is one of the most important neglected tropical diseases of humans worldwide. The epidemiology of *Ascaris* infection appears to have changed with improvements in sanitation and mass drug administration, but there is no recent information on prevalence worldwide. Here, we performed a systematic review and meta-analysis to assess the global prevalence of human *Ascaris* infection from 2010 to 2021.

**Methods:**

We searched MEDLINE/PubMed, and Scopus databases for studies measuring prevalence of *Ascaris* infection, published between 1 January 2010 and 1 January 2022. We included studies of the general human population in endemic regions, which used accepted coprodiagnostic methods, and excluded studies of people with occupations with an increased risk or probability of ascariasis and/or specific diseases other than ascariasis. We applied random-effects models to obtain pooled prevalence estimates for six sustainable development goal regions of the world. We extrapolated the prevalence estimates to the global population in 2020, to estimate the number of individuals with *Ascaris* infection. We conducted multiple subgroup and meta-regression analyses to explore possible sources of heterogeneity, and to assess relationships between prevalence estimates and demographic, socio-economic, geo-climatic factors.

**Results:**

Of 11,245 studies screened, we analysed 758 prevalence estimates for a total number of 4,923,876 participants in 616 studies from 81 countries. The global prevalence estimated was 11.01% (95% confidence interval: 10.27–11.78%), with regional prevalences ranging from 28.77% (7.07–57.66%) in Melanesia (Oceania) to 1.39% (1.07–1.74%) in Eastern Asia. We estimated that ~ 732 (682–782) million people harboured *Ascaris* worldwide in 2021. The infected people in Latin America and the Caribbean region had a higher prevalence of high intensity infection (8.4%, 3.9–14.1%). Prevalence estimates were higher in children, and people in rural communities or in countries or regions with lower income and human development indices. There was a trend for a higher prevalence in regions with increasing mean annual relative humidity, precipitation and environmental temperature.

**Conclusions:**

Our findings indicate that, despite a renewed commitment by some communities or authorities to control ascariasis, a substantial portion of the world’s human population (> 0.7 billion) is infected with *Ascaris*. Despite the clinical and socioeconomic importance of ascariasis, many past routine surveys did not assess the intensity of *Ascaris* infection in people. We propose that the present findings might stimulate the development of customised strategies for the improved control and prevention of *Ascaris* infection worldwide.

**Supplementary Information:**

The online version contains supplementary material available at 10.1186/s40249-022-01038-z.

## Background

Human ascariasis is one of the most important neglected tropical diseases (NTDs) worldwide [1]. It is caused by the intestinal nematode of the genus *Ascaris*—a soil-transmitted helminth (STH) [2]. Transmission occurs as a consequence of the accidental ingestion of embryonated eggs from contaminated soil, food and/or water. *Ascaris* eggs are resistant and have the potential to survive for long periods of time, particularly under warm and moist conditions [3]. Ascariasis is prevalent and has been estimated to affect ~ 819 million people [4]. The intensity of *Ascaris* infection is highest in children of 5 to 15 years of age, and has an over-dispersed or aggregated distribution, with most individuals harbouring light infections, and a relatively small proportion of the population harbouring heavy infection [5]. Furthermore, there is consistency in the pattern of re-infection or predisposition in humans [6].

Ascariasis can have acute and chronic manifestations, the latter being associated with significant nutritional and growth deficits [7]. Moreover, there is an association between ascariasis and impaired cognitive development, but the mechanism/s is/are not yet well understood [8]. *Ascaris* undergoes a larval migration via the liver and the lungs before establishing in the small intestine as a dioecious adult stage. This hepatopulmonary migration has significant, but underestimated pathological and public health impact, such as hepatic and pulmonary disorders [9]. Acute complications caused by adult worms might include intestinal impaction and/or obstruction of biliary and/or pancreatic ducts [7]. Despite the apparent rarity of acute ascariasis, such complications might have a high fatality rate [10].

The commonest method used for the diagnosis for *Ascaris* infection is the microscopic detection of eggs in faecal samples [11]. Counting adult worms upon expulsion following treatment is regarded as a “gold standard” for estimating the intensity of infection [11], but this approach is very rarely performed and only usually in the context of research projects, rather than routine parasite surveys or monitoring during large-scale deworming programmes.

Key epidemiological measures used to determine the extent of *Ascaris* infection are the prevalence of infection (% of persons infected in a particular population) and the intensity of infection. Intensity can be expressed as the arithmetic mean number of worms (“worm burden”), of eggs per gram of faeces (EPG), geometric mean EPG, or median EPG. The central importance of knowledge of prevalence and intensity of infection to the understanding of the epidemiology and impact of macroparasites in humans and other animals cannot be underestimated [5]. However, the number of routine surveys and monitoring programmes that utilise a measure of intensity remains low. In 2000, World Health Organization (WHO) recommended EPG thresholds (“cut-offs”) that can be used to classify infection intensity as low, moderate and high EPG [12].

Over the last few decades, large-scale deworming programmes have been implemented to “control” STHs in endemic regions. Despite variations in treatment efficacy among STHs, albendazole and mebendazole appear to remain efficacious anthelmintics against human ascariasis [13]. However, anthelmintic treatment alone will not lead to a marked reduction or elimination of ascariasis without accompanying improvements in socioeconomic conditions and the provision of clean water, improved sanitation and hygiene [14]. Thus, WHO set new targets that included the elimination of morbidity due to STHs (defined as the prevalence of moderate and heavy infection intensities of < 2%) in preschool- and school-age children by 2030 and universal access to at least basic sanitation and hygiene by 2030 in STH-endemic areas [15].

Pigs infected with *Ascaris* can represent a reservoir for human infection [16, 17], despite controversies about the species status of *Ascaris* of humans and pigs [16, 18, 19]. Part of the motivation to undertake the present review was the need to critically re-evaluate the extent of ascariasis in humans in endemic regions, since the last estimate in 2010, published in 2014 [4]. Here, we report a comprehensive systematic review and meta-analysis to estimate the prevalence and intensity of *Ascaris* infection in the general human population in endemic regions from 1 January 2010 to 1 January 2022. We also evaluated the impact of geographical, climatic and socio-demographic factors on the prevalence of *Ascaris* infection in different countries and regions.

## Methods

### Search strategy and selection criteria

This meta-analysis study was designed and conducted in accordance with the Cochrane Handbook of Systematic Reviews [20], and is reported following the Preferred Reporting Items for Systematic Reviews and Meta-Analyses (PRISMA) guidelines [21] (Additional file 5: Checklist S1). Five investigators (C.H., H.B., A.A., G.D. and A.R.) began the search of the relevant studies on April 2020, through MEDLINE/PubMed, and Scopus, without language restriction. An updated search was performed on February 2022. Searches were limited to studies published between 1 January 2010 and 1 January 2022. The search terms used included: “ascariasis”, “*Ascaris*”, “*Ascaris lumbricoides*”, “intestinal parasites”, intestinal helminths”, “soil transmitted helminths”, “prevalence”, “incidence”, “epidemiology” and “occurrence”. These keywords were combined using Boolean operators ‘OR’ and/or ‘AND’ in database searches (Additional file 4: Fig. S1). To identify additional studies, the same investigators independently searched the Google Scholar engine, and scrunitised the reference lists of eligible studies. All citations retrieved were imported to Endnote software X8 (Thompson and Reuters, Philadelphia, USA), and 10,185 duplicates and irrelevant papers were deleted, leaving 1060 citations. Four independent investigators (S.M., S.V., A.A. and A.R.) reviewed the titles, abstracts, and full texts of articles for eligibility. The online tool “Google Translate” (https://translate.google.com/) was used to assist the translation of articles published in languages other than English.

### Selection criteria

Studies were considered eligible for inclusion if they were peer-reviewed, observational studies which reported the prevalence of *Ascaris* infection*.* Two trained researchers applied inclusion criteria: a sample size of at least 50; published after 1 January 2010; the tested population should be representative of the general population (i.e. randomly-selected people of different ages, socioeconomic status and ethnic backgrounds, without occupations or specific diseases increasing the probability of acquiring ascariasis); and studies that used an internationally accepted coprodiagnostic method with suitable performance characteristics (e.g., Kato-Katz, formalin-ether, McMaster, FLOTAC or Mini-FLOTAC techniques). In order to reduce bias in the estimation of ascariasis prevalence in the general population, studies of the following population groups were excluded: patients with gastrointestinal disorders, patients with diarrhoea, HIV^+^ patients, patients receiving corticosteroid treatment, patients with any immunodeficiency, haemodialysis patients, patients with tuberculosis, inmates in prisons, pregnant women, mentally retarded patients, immigrants, patients with atopy and other allergies, workers exposed to wastes, wastewater and faecal sludge. In addition, studies were excluded if they were: (1) comparing diagnostic methods; (2) serological investigations; (3) reporting an intervention approach, without the availability of baseline (reference) data; (4) used datasets that overlapped with those of other articles; (5) recorded prevalence after mass drug administration; (6) were performed in non-endemic areas, such as European and North American countries; (7) had a sample size of ≤ 50 individuals; (8) were case reports or series; or (9) were letters, commentaries, reviews or systematic reviews without original data.

### Extraction and quality evaluation of data

A data collection form was developed in Microsoft Excel (version 2016; Microsoft Corporation, Redmond, USA); data were extracted independently by five investigators (H.B., S.V., G.D., S.G., S.M. and A.A.), and unanimity was reached on discrepancies after discussion, and also consultation with the senior investigators (C.H. and A.R.). Data were extracted for the following domains: (I) study characteristics (the first author's last name, year of publication, diagnostic methods, country; city, ‘sustainable development goal’ (SDG)-regions or sub-regions [22]); (II) participant characteristics [type of population (children, adult or both), the number of participants, the number of people that tested positive for *Ascaris* infection, age, sex, residence and intensity of infection (low, moderate, high)]; (III) socio-economic variables (World Bank–income category [23], gross national income per capita [23] and the human development index [HDI] [24]); and (IV) geo-climatic factors (latitude, longitude, mean relative humidity, mean annual precipitation and mean environmental temperature). The data sources for geo-climatic factors were: https://gps-coordinates.org/, https://www.timeanddate.com/ and https://en.climate-data.org/. Furthermore, we recorded total global, regional populations (both sexes) in 2020, estimated by the United Nations [25]. For interventional studies, we extracted only baseline data, and for case-control studies, we only extracted data for healthy people. The data on intensity of *Ascaris* infection were extracted from studies that assessed and graded intensity according to the criteria defined by WHO [12]. The investigators independently assessed the risk of bias for each study using the Joanna Briggs Institute (JBI) Critical Appraisal Tools for cross-sectional, case-control, cohort and randomized controlled studies, and each study was categorized to have a low, moderate or high risk of bias, as recommended for these tools [26–28].

### Meta-analysis

All statistical analyses were conducted using Stata statistical software (v.13 Stata Corp., College Station, TX, USA). The prevalence of *Ascaris* infection in each study was calculated by dividing the number of test-positive cases by the study population. To estimate the pooled prevalence of *Ascaris* infection, we used a DerSimonian and Laird random-effects model (REM) [29]. REM is capable of incorporating proportions close to, or at the margins (i.e., with a very low or very high prevalence), into conservative pooled prevalence estimates and 95% confidence intervals (*CI*s). We calculated the pooled prevalence rates at a 95% *CI* using the ‘metaprop’ command in Stata software. The pooled prevalence in each country was estimated by synthesizing the prevalence rates of all studies from the same country. We stratified estimates into the six endemic SDG regions and the 14 SDG sub-regions (Table 1). We estimated heterogeneity using the χ^2^ test with Cochran’s Q statistic and quantified with *I*^2^; *I*^2^ of > 75% was considered to reflect substantial heterogeneity [30]. To calculate the number of people infected with *Ascaris*, we extrapolated prevalence estimates to the total human population (in 2020) living in a country and/or a region—according to the UN Population Division [25]. Data were entered into ArcGIS 10.2 (ESRI, Redlands, CA, US) to produce maps presenting *Ascaris* infection prevalence estimates on a country level.

**Table 1:** Global and regional prevalence of *Ascaris* infection, and estimated numbers of infected people (results from 758 datasets performed in 81 countries)

SDG regions^a^ (number of datasets available for a particular region)	Number of people screened (total)	Number of test positive people	Pooled prevalence, % (95% *CI*)	Estimated global or regional population (2020)	Estimated number of infected people in millions (95% *CI*)
**Global (758)**^b^	**4,923,876**	**164,879**	**11.01 (10.27–11.78)**	**6,646,738,000**	**731,805,853 (682,619,992–782,985,736)**
**Eastern and South-eastern Asia (162)**	**1,153,432**	**35,374**	**9.82 (8.44–11.30)**	**2,346,709,000**	**230,446,823 (198,062,239–265,178,117)**
South-eastern Asia (97)	174,530	27,780	19.06 (15.51–22.87)	668,620,000	127,438,972 (103,702,962–152,913,394)
Eastern Asia (65)	978,902	7594	1.39 (1.07–1.74)	1,678,090,000	23,325,451 (17,955,563–29,198,766)
**Latin America and the Caribbean (110)**	**182,807**	**13,973**	**12.75 (10.75–14.88)**	**653,962,000**	**83,380,155 (70,300,915–97,309,545)**
South America (86)	173,479	12,352	11.58 (9.49–13.84)	430,760,000	49,882,008 (40,879,124–59,617,184)
Central America (20)	7184	1424	18.83 (13.04–25.42)	179,670,000	33,831,861 (23,428,968–45,672,114)
Caribbean region (4)	2144	197	11.41 (4.38–21.09)	43,532,000	4,967,001 (1,906,701–9,180,898)
**Sub-Saharan Africa (353)**	**774,117**	**92,804**	**11.66 (10.56–12.81)**	**1,094,366,000**	**127,603,075 (115,565,049–140,188,284)**
Western Africa (117)	138,888	10,583	1107 (8.75–13.63)	401,861,000	44,486,012 (35,162,837–54,773,654)
Eastern Africa (184)	594,973	75,655	11.12 (9.86–12.45)	445,405,000	49,529,036 (43,916,933–55,452,922)
Southern Africa (6)	3426	697	14.48 (6.27–25.30)	67,504,000	9,774,579 (4,232,500–17,078,512)
Middle Africa (46)	36,830	5869	15.31 (11.39–19.69)	179,596,000	27,496,147 (20,455,984–35,362,452)
**Central and Southern Asia (92)**	**389,695**	**15,434**	**12.91 (10.01–16.13)**	**2,014,709,000**	**260,098,931 (201,672,370–324,972,561)**
Southern Asia (89)	375,197	14,839	13.10 (10.01–16.54)	1,940,370,000	254,188,470 (194,231,037–320,937,198)
Central Asia (3)	14,498	595	8.02 (0.24–24.90)	74,339,000	5,961,987 (178,413–18,510,411)
**Northern Africa &Western Asia (37)**	**2,422,161**	**6869**	**2.04 (1.47–2.70)**	**525,869,000**	**10,727,727 (7,730,274–14,198,463)**
Northern Africa (7)	20,313	239	4.01 (0.85–9.20)	246,232,000	9,873,903 (2,092,972–22,653,344)
Western Asia (30)	2,401,848	6630	1.71 (1.14–2.38)	279,637,000	4,781,792 (3,187,861–6,655,360)
**Oceania** (Melanesia sub-region) **(4)**^c^	**1664**	**425**	**28.77 (7.07–57.66)**	**11,123,000**	**3,200,087 (786,396–6,413,521)**

*SDG*, Sustainable Development Goal; *CI,* confidence interval

^a^Sustainable Development Goal regions as defined by the United Nations

^b^Only population of endemic area were considered in the analyses and population of North America, Europe and Australia & New Zealand are not considered

^c^Countries (Solomon Islands and Papua New Guinea) with eligible data were only from Melanesia sub-region, therefore only population of this sub-region were considered in extrapolation to estimate the number of infected people

We undertook several subgroup, as well as univariate and multivariate meta-regression, analyses to explore possible sources of heterogeneity and effects of socio-economic, study characteristics, geo-climatic parameters on the prevalence of *Ascaris* infection in people. These analyses were performed using the ‘metareg’ command in Stata [31] in relation to study participants (children, adults and total population); diagnostic method used; age, sex and residence; country income level (low, lower-middle, upper-middle or high) and country HDI (low, medium, high or very high); risk of bias levels for each study (low, moderate or high); geographical latitude and longitude; mean annual environmental temperature, relative humidity and precipitation; year of publication; and the start and end dates of sampling. Results were considered as statistically significant if the *P* value was < 0.1.

## Results

### Study characteristics

**Fig. 1:**
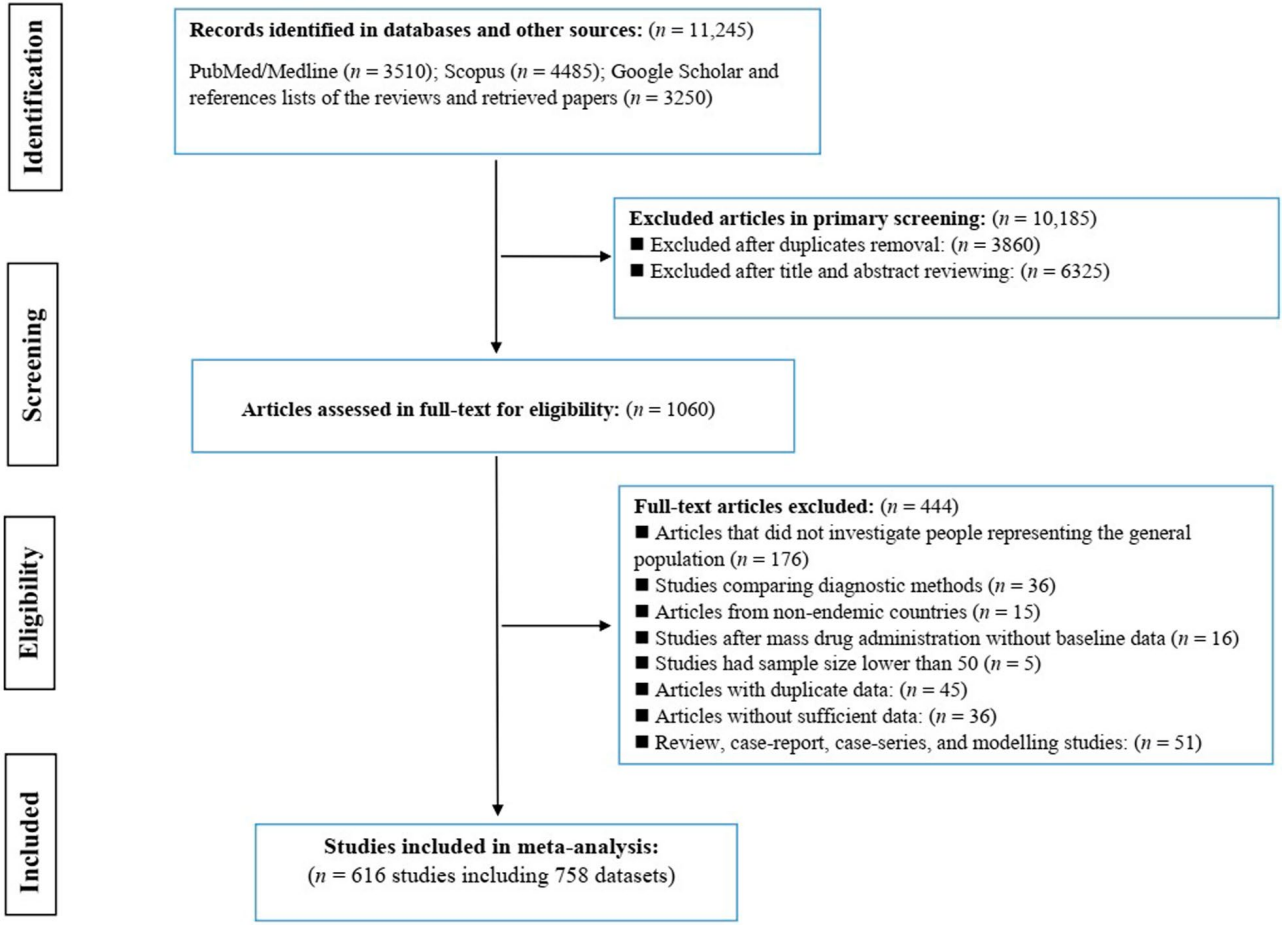
The literature search strategy and study-selection process, indicating numbers of studies excluded and included

Our search of electronic databases identified a total of 11,245 articles; following the removal of duplicate articles and a critical appraisal of article titles and abstracts, 1060 potentially relevant articles were identified for full-text evaluation (Fig. 1). After applying the eligibility criteria, 616 articles containing 758 datasets were included in the quantitative synthesis (Fig. 1); these datasets represented 4,923,876 people from 81 countries in six SDG regions and 14 SDG sub-regions. Of these datasets, 353 were from sub-Saharan Africa, 162 from Eastern & South-eastern Asia, 110 from Latin America and the Caribbean, 92 from Central and Southern Asia, 37 from Northern Africa and Western Asia, and four from Oceania. Considering SDG sub-regions; Eastern Africa (*n* = 184), Western Africa (*n* = 117), South-Eastern Asia (*n* = 97), Southern Asia (*n* = 89), and South America (*n* = 86) had highest eligible datasets, while Northern Africa (*n* = 7), Southern Africa (*n* = 6), Caribbean region (*n* = 4), Oceania (*n* = 4) and Central Asia (*n* = 3) had the lowest eligible datasets. The main characteristics of the studies included are provided in Additional file 1: Table S1. In this study, it should be noted that only populations in endemic areas were considered in the analyses; populations in North America, Europe and Australia & New Zealand were not considered.

### Global and regional prevalence of *Ascaris* infection

**Fig. 2:**
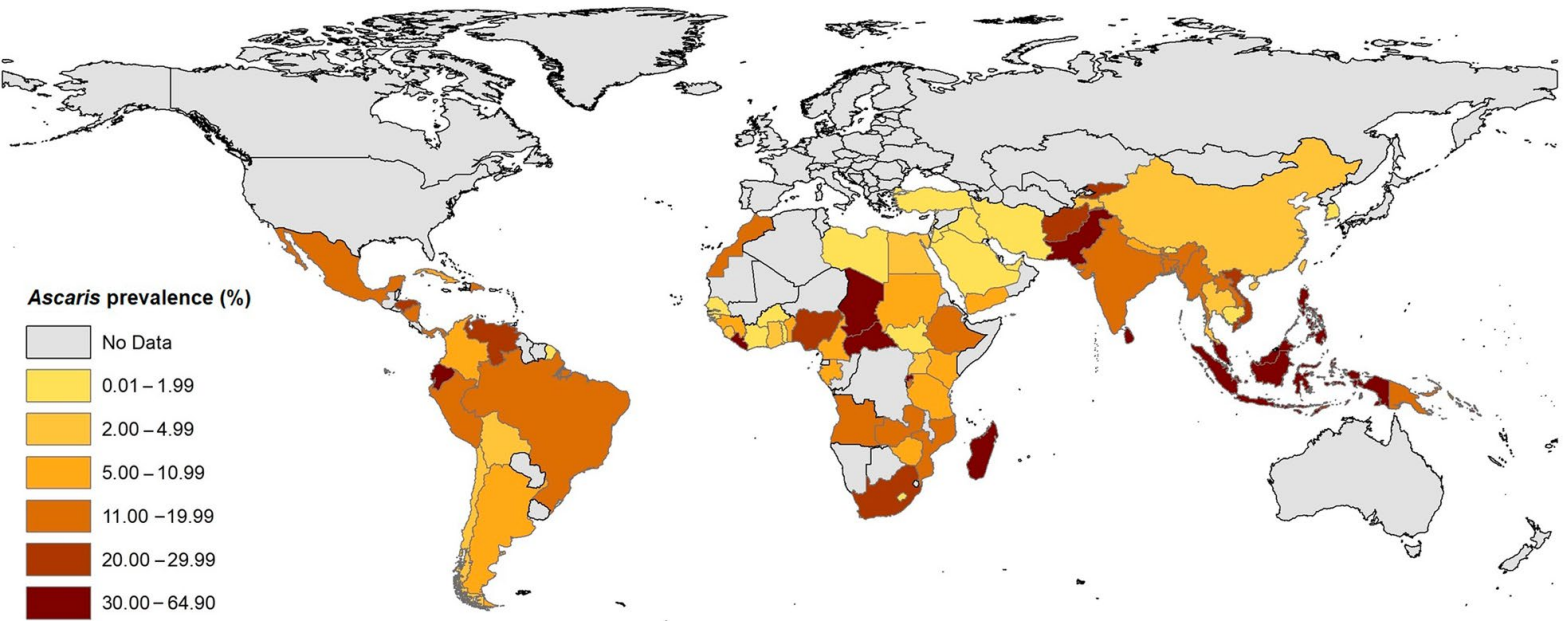
Estimated *Ascaris* infection prevalence rates in the general human population in different countries using the geographic information system (GIS)

Overall, 164,879 people from a general population of 4,923,876 tested positive for *Ascaris* infection, indicating a pooled prevalence of 11.01% (95% *CI:* 10.27–11.78%). Significant heterogeneity (*I*^2^ = 99.8%, *P* < 0.001) was found between studies. The highest pooled prevalence estimates were for Central & Southern Asia (12.91%, 10.01–16.13%), Latin America & the Caribbean (12.75%, 10.75–14.88%) and sub-Saharan Africa (11.66%, 10.56–12.81%), while the lowest prevalence was from Northern Africa & Western Asia (2.04%, 1.47–2.70%) (Table 1). Considering the SDG sub-regions, the highest prevalence rates were: 28.77% (7.07–57.66%) in Melanesia (Oceania), 19.06% (15.51–22.87%) in South-Eastern Asia, 18.83% (13.04–25.42%) in Central America and 15.31% (11.39–19.69%) in Middle Africa, and the lowest prevalences were 1.39% (1.07–1.74%) in Eastern Asia, 1.71% (1.14–2.38%) in Western Asia, and 4.01% (0.85–9.20%) in Northern Africa (Table 1). Countries (for which ≥ 3 eligible studies were available) with the highest prevalence rates were Chad (48.19%), Liberia (42.45%), Ecuador (39.58%), Madagascar (37.16%), the Philippines (34.69%), Solomon Islands (33.96%), Indonesia (32.02%), Rwanda (31.22%) and Sri Lanka (30.81%) (Table 2). Figure 2 shows the *Ascaris* infection prevalence estimates for individual countries.

**Table 2:** Prevalence estimates of *Ascaris* infection, and estimated numbers of infected people in 81 countries

Country (number of datasets available for a particular country)	Number of people screened (total)	Number of test positive people	Pooled prevalence, % (95% *CI*)	Estimated population size (2020)	Estimated number of infected people (95% *CI*)
Afghanistan (6)	6626	1434	20.32 (17.44–23.36)	38,928,346	7,910,231 (6,789,103–9,093,661)
Angola (4)	1289	178	13.11 (4.40–25.43)	32,866,272	4,308,768 (1,446,115–8,357,892)
Argentina (18)	8799	346	5.40 (2.75–8.83)	45,195,774	2,440,571 (1,242,883–3,990,786)
Bangladesh (3)	11,593	1601	16.33 (1.92–40.75)	164,689,383	26,893,776 (3,162,036–67,110,923)
Benin (32)	28,094	1671	6.76 (5.45–8.19)	12,123,200	819,528 (657,077–992,890)
Bhutan (4)	7375	19	0.36 (0.01–1.16)	771,608	2778 (77–8950)
Bolivia (4)	1286	48	2.86 (0.01–10.01)	11,673,021	333,848 (1167–1,168,469)
Brazil (35)	68,648	7211	13.57 (9.42–18.34)	212,559,417	28,844,312 (20,023,097–38,983,397)
Burkina Faso (2)	3899	4	0.06 (0.01–0.19)	20,903,273	12,542 (2090–39,716)
Burundi (4)	38,605	3645	14.04 (7.66–21.97)	11,890,784	1,669,466 (910,834–2,612,405)
Cambodia (6)	39,182	1522	1.03 (0.02–3.23)	16,718,965	172,205 (3343–540,022)
Cameroon (23)	24,694	3039	10.35 (7.69–13.35)	26,545,863	2,747,496 (2,041,376–3,543,872)
Central African Republic (2)	1052	681	64.90 (61.97–67.77)	4,829,767	3,134,518 (2,993,006–3,273,133)
Chad (3)	1621	679	48.19 (32.14–64.43)	16,425,864	7,915,624 (528,980–10,583,184)
Chile (2)	68,245	2461	3.39 (3.25–3.53)	19,116,201	648,039 (621,276–674,801)
China (36)	947,639	7584	3.99 (3.27–4.77)	1,439,323,776	57,429,018 (47,065,887–68,655,744)
Colombia (9)	7260	790	9.74 (5.46–15.05)	50,882,891	4,955,993 (321,205–7,657,875)
Côte d'Ivoire (13)	27,380	318	0.82 (0.46–1.28)	26,378,274	216,302 (121,340–337,642)
Cuba (3)	1590	100	9.44 (2.95–18.89)	11,326,616	1,069,232 (334,135–2,139,598)
DR Congo (8)	5800	802	14.05 (3.84–29.16)	89,561,403	12,583,377 (3,439,158–26,116,105)
Ecuador (5)	2232	766	39.58 (24.36–55.91)	17,643,054	6,983,121 (4,297,848–9,864,231)
Egypt (5)	2013	111	3.84 (0.67–9.21)	102,334,404	3,929,641 (685,640–9,424,998)
Ethiopia (88)	345,265	46,976	13.46 (12.27–14.69)	114,963,588	15,474,099 (14,106,032–16,888,151)
French Guiana (1)	9555	20	0.21 (0.13–0.32)	298,682	627 (388–956)
Gabon (4)	1582	181	7.98 (2.01–17.27)	2,225,734	177,613 (44,737–384,384)
Ghana (7)	3176	89	3.63 (1.30–6.98)	31,072,940	1,127,948 (40,395–2,168,891)
Guinea (1)	420	34	8.10 (5.67–11.13)	13,132,795	1,063,756 (744,629–1,461,680)
Guinea-Bissau (1)	1274	1	0.08 (0.01–0.44)	1,968,001	1574 (196–8659)
Honduras (3)	2969	681	23.24 (17.10–30.01)	9,904,607	2,301,830 (1,693,687–2,972,372)
India (29)	294,942	7657	15.88 (9.07–24.14)	1,380,004,385	219,144,696 (125,166,397–333,133,058)
Indonesia (10)	8488	2443	32.02 (20.16–45.17)	273,523,615	87,582,261 (55,142,361–123,550,617)
Iran (11)	28,546	47	0.22 (0.02–0.55)	83,992,949	184,784 (16,798–461,961)
Iraq (5)	1,897,756	420	0.27 (0.13–0.45)	40,222,493	108,600 (52,289–181,001)
Jordan (1)	21,906	84	0.38 (0.31–0.47)	10,203,134	38,771 (31,629–47,954)
Kenya (30)	127,853	17,156	8.90 (5.66–12.87)	53,771,296	4,785,645 (3,043,455–6,920,365)
Republic of Korea (29)	31,263	10	0.01 (0.001–0.03)	51,269,185	5127 (512–15,380)
Kyrgyzstan (1)	1262	292	23.14 (20.84–25.57)	6,524,195	1,509,698 (1,359,642–1,668,236)
Laos PDR (26)	26,107	2610	11.19 (7.34–15.71)	7,275,560	814,135 (534,026–1,142,990)
Lebanon (1)	7477	14	0.19 (0.10–0.31)	6,825,445	6828 (12,968–21,159)
Lesotho (1)	301	0	0.01 (0.001–1.22)	2,142,249	214 (21–26,135)
Liberia (3)	2068	844	42.45 (12.12–76.38)	5,057,681	2,146,985 (612,990–3,863,056)
Libya (1)	18,000	90	0.50 (0.40–0.61)	6,871,292	34,356 (27,485–41,914)
Madagascar (4)	3349	846	37.16 (4.63–78.90)	27,691,018	10,289,982 (1,282,094–21,848,213)
Malaysia (22)	9243	2915	30.60 (19.19–43.35)	32,365,999	9,903,995 (6,211,035–14,030,660)
Mexico (8)	870	221	19.65 (6.19–37.96)	128,932,753	25,335,286 (7,980,937–48,942,873)
Morocco (1)	300	38	12.67 (9.12–16.97)	36,910,560	4,676,567 (3,366,243–6,263,722)
Mozambique (3)	1539	212	16.23 (0.01–52.81)	31,255,435	5,072,757 (3125–16,505,995)
Myanmar (6)	5126	833	19.04 (7.03–35.11)	54,409,800	10,359,626 (3,825,009–19,103,281)
Nepal (16)	17,888	1712	7.95 (1.81–17.75)	29,136,808	2,316,376 (527,376–5,171,783)
Nicaragua (8)	3245	508	17.44 (8.45–28.80)	6,624,554	1,155,322 (559,774–1,907,871)
Nigeria (49)	24,664	6849	23.77 (18.13–29.90)	206,139,589	48,999,380 (37,373,107–61,635,737)
Pakistan (12)	5113	1013	30.09 (14.62–48.32)	220,892,340	66,466,505 (32,294,460–106,691,000)
Palestine (5)	227,005	3588	0.70 (0.01–2.47)	5,101,414	35,709 (510–126,005)
Panama (1)	100	14	14.01 (7.87–22.37)	4,314,767	604,499 (339,572–965,213)
Papua New Guinea (1)	627	95	15.15 (12.43–18.20)	8,947,024	1,355,474 (1,112,115–1,628,358)
Peru (6)	2935	269	12.75 (6.52–20.62)	32,971,854	4,203,911 (2,149,764–6,798,796)
Philippines (13)	60,921	15,748	34.69 (27.01–42.79)	109,581,078	38,013,676 (29,597,849–46,889,743)
Rwanda (4)	6568	2999	31.22 (19.30–44.54)	12,952,218	4,043,682 (2,499,778–5,768,918)
Saint Lucia (1)	554	97	17.51 (14.43–20.93)	183,627	32,153 (26,497–38,433)
Sao Tome and Principe (2)	792	309	37.82 (34.47–41.23)	219,159	82,886 (75,544–90,359)
Saudi Arabia (2)	1419	13	0.77 (0.34–1.34)	34,813,871	268,066 (118,367–466,505)
Senegal (2)	3741	34	0.81 (0.54–1.13)	16,743,927	135,625 (90,417–189,206)
Sierra Leone (5)	10,635	610	4.13 (1.99–6.98)	7,976,983	329,449 (158,742–556,793)
Solomon Islands (3)	1037	330	33.96 (3.28–76.10)	686,884	233,265 (22,529–522,718)
South Sudan (1)	450	3	0.67 (0.14–1.94)	11,193,725	74,997 (15,671–217,158)
South Africa (5)	3125	697	20.12 (14.27–26.70)	59,308,690	11,932,908 (8,463,350–15,835,420)
Sri Lanka (8)	3114	1356	30.81 (21.75–40.69)	21,413,249	6,597,422 (4,657,381–8,713,051)
Sudan (3)	411	59	9.55 (0.01–33.44)	43,849,260	4,187,604 (4384–14,663,192)
Tajikistan (2)	13,236	303	2.24 (1.99–2.50)	9,537,645	213,643 (189,799–238,441)
Tanzania (23)	25,240	2101	7.3 (1.83–16.14)	59,734,218	4,360,598 (1,093,136–9,641,102)
Thailand (9)	19,490	213	3.62 (0.79–8.27)	69,799,978	2,526,759 (551,419–5,772,458)
Timor-Leste (2)	2832	803	28.09 (26.44–29.76)	1,318,445	370,351 (348,596–392,369)
Togo (2)	33,537	129	0.38 (0.32–0.45)	8,278,724	31,459 (26,492–37,254)
Turkey (8)	232,834	2205	1.04 (0.27–2.26)	84,339,067	877,126 (227,715–1,906,063)
Uganda (15)	13,745	386	3.46 (2.04–5.22)	45,741,007	1,582,639 (933,116–2,387,680)
United Arab Emirates (2)	10,600	19	0.01 (0.001–0.03)	9,890,402	990 (98–2967)
Venezuela (6)	4519	441	21.04 (6.87–40.20)	28,435,940	5,982,921 (1,953,549–11,431,248)
Viet Nam (3)	3141	693	25.31 (1.73–63.40)	97,338,579	24,636,394 (1,683,957–61,712,659)
Yemen (6)	2851	287	10.36 (4.97–17.37)	29,825,964	3,089,969 (1,482,350–5,180,770)
Zambia (5)	4677	475	11.57 (7.64–16.19)	18,383,955	2,127,023 (1,404,534–2,976,362)
Zimbabwe (4)	27,271	797	5.08 (2.48–8.52)	14,862,924	755,036 (368,600–1,266,321)

*CI,* confidence interval; *DR* Congo, Democratic Republic of the Congo; *Laos PDR,* the Lao People’s Democratic Republic

An extrapolation to the 2020 world population indicated that ~ 731.8 million (range: 682,619,992 to 782,985,736) people harboured *Ascaris* infection. The sub-regions with the highest burden of *Ascaris* infection were Southern Asia (~ 254.1 million people), South-eastern Asia (~ 127.4 million people), South America (~ 49.8 million people), Eastern Africa (~ 49.5 million people) and Western Africa (~ 44.4 million people). More detail on the global and regional *Ascaris* infection prevalences and burdens are given in Table 1.

### Intensity of *Ascaris* infection

In total, 137 of 758 datasets included information on the intensity of *Ascaris* infection (Additional file 2: Table S2). According to WHO guidelines, we appraised these data sets and estimated the prevalence of low, moderate and high intensities among infected people in each SDG region (for which ≥ 3 eligible studies were available). Our analyses showed that the highest prevalence of high intensity of *Ascaris* infection among infected people was found in Latin America and the Caribbean regions (8.35%, 3.88–14.11%), followed by Eastern and South-Eastern Asia (3.80%, 1.07–7.59%), Central and Southern Asia (0.62%, 0.01–0.63%), and Sub-Saharan Africa (0.26%, 0.01–0.97%). Our findings indicated that the prevalence of high intensity infection was lower in 2018–2021 (1.43%, 0.44–2.79%) compared with 2010–2013 (3.32%, 1.11–6.31%). Detail on low and moderate infection intensities are presented in Table 3.

**Table 3:** Intensity of *Ascaris* infection based upon numbers of infected people with cut-offs for low, medium or high intensity infection

	Number of datasets	Number of people screened (total)	Number of test positive people	Intensity of infection		
				Low, % (95% *CI*)	Moderate, % (95% *CI*)	High, % (95% *CI*)
Global						
Central and Southern Asia	8	20,771	5891	88.54 (73.39–98.19)	9.43 (1.50–21.93)	0.62 (0.01–0.63)
Eastern and South-Eastern Asia	36	405,721	7893	66.59 (56.04–76.45)	24.67 (17.77–32.19)	3.80 (1.07–7.59)
Latin America and the Caribbean	19	12,005	2769	55.96 (48.28–63.51)	31.20 (24.01–38.88)	8.35 (3.88–14.11)
Sub-Saharan Africa	81	143,231	14,642	83.31 (78.95–87.31)	12.22 (9.15–15.59)	0.26 (0.01–0.97)
Year of publication						
2010–2013	31	26,710	4429	70.51 (59.43–80.57)	20.93 (13.82–28.95)	3.32 (1.11–6.31)
2013–2017	50	72,982	13,153	77.94 (69.83–85.19)	15.15 (10.16–20.80)	1.41 (0.11–3.62)
2018–2021	64	482,619	13,924	77.55 (72.13–82.59)	17.78 (13.82–22.07)	1.43 (0.44–2.79)

*CI,* confidence interval

### Prevalence of *Ascaris* infection according to sex, age, residence and population

According to the type of population studied, 494 datasets related to children, 256 to both children and adults (all age groups) and eight studies to adults. In subgroup analysis, the global *Ascaris* infection was 13.50% (12.39–14.65%) in studies of children, 6.89% (6.19–7.63%) in studies of all age groups and 8.85% (1.53–20.25%) in studies recruiting only adults (Table 4).

**Table 4:** Prevalence estimates of *Ascaris* infection, according to a priori-defined subgroups and socio-demographic geographic parameters

Variable: Subgroup	Number datasets	Number of people screened (total)	Number of test positive people	Pooled prevalence, % (95% *CI*)	Prevalence ratio, % (95% *CI*)
Type of population					
Children	494	969,034	112,608	13.50 (12.39–14.65)	8.82 (8.73–8.91)
Adult	8	8479	290	8.85 (1.53–20.25)	2.59 (2.31–2.90)
Total population	256	3,946,363	51,981	6.89 (6.19–7.63)	1
Gender					
Male	138	477,521	22,559	14.61 (12.64–16.68)	1
Female	138	417,804	22,260	14.67 (12.61–16.86)	1.12 (1.10–1.14)
Residence					
Urban	143	75,535	6222	10.02 (7.29–13.12)	1
Rural	143	118,029	17,341	17.33 (14.21–20.69)	1.78 (1.73–1.83)
Age, years					
≤ 5	51	17,863	2192	15.19 (10.69–20.29)	1.97 (1.87–2.07)
6–11	50	64,428	4008	15.96 (11.68–20.74)	1
12–18	71	176,149	23,029	14.14 (11.67–16.80)	2.10 (2.03–2.17)
19–30	9	7538	1373	19.52 (9.55–31.65)	2.92 (2.76–3.09)
31–50	5	2323	637	22.53 (8.93–39.94)	4.40 (4.09–4.74)
≥ 51	4	1467	378	25.82 (23.59–28.12)	4.14 (3.77–4.54)
Diagnostic method used					
Routine parasitological methods^a^	353	3,207,496	43,680	9.22 (8.45–10.01)	1
Kato-Katz	368	1,693,547	117,567	12.68 (11.40–14.01)	5.09 (5.04–5.15)
Others^b^	37	22,833	3275	11.90 (7.29–17.43)	10.53 (10.19–10.88)
Income					
Low	153	473,915	59,829	13.23 (11.64–14.91)	5.34 (5.15–5.54)
Lower middle	385	1,072,320	77,869	12.17 (10.89–13.52)	3.07 (2.96–3.19)
Upper middle	178	3,252,040	24,217	9.97 (9.14–10.83)	0.31 (0.30–0.32)
High	42	125,601	2964	0.88 (0.33–1.64)	1
Human development index (HDI)					
Low	273	583,335	70,802	12.31 (11.01–13.67)	5.64 (5.51–5.77)
Medium	210	2,524,185	43,210	11.71 (10.24–13.25)	0.79 (0.77–0.81)
High	190	1,444,298	42,864	11.45 (10.17–12.79)	1.37 (1.34–1.41)
Very high	85	372,058	8003	5.01 (3.90–6.25)	1
Risk of bias					
Low	378	4,405,898	139,663	7.81 (6.94–8.73)	1
Moderate	269	477,606	21,430	14.28 (12.68–15.96)	1.41 (1.39–1.43)
High	111	40,372	3786	16.23 (12.77–20.02)	2.95 (2.86–3.05)
Year of publication					
2010–2013	206	513,557	24,369	9.18 (7.87–10.58)	2.24 (2.20–2.27)
2014–2017	300	1,221,037	73,006	12.61 (11.15–14.15)	2.82 (2.79–2.85)
2018–2021	252	3,189,282	67,504	10.73 (9.57–11.95)	1
Year of start sampling					
Before 2010	197	1,500,014	39,518	8.52 (7.57–9.53)	1
2010–2015	412	2,862,290	108,649	12.59 (11.22–14.02)	1.44 (1.42–1.45)
2016–2021	149	561,572	16,712	10.24 (8.53–12.09)	1.12 (1.10–1.14)
Year of end sampling					
Before 2010	150	504,076	28,385	9.91 (8.21–11.74)	2.04 (2.01–2.07)
2010–2015	413	3,390,700	108,179	12.15 (11.01–13.34)	1.15 (1.14–1.17)
2016–2021	195	1,029,100	28,315	9.57 (8.42–10.79)	1

*CI,* confidence interval

^a^Direct wet mount, Formalin-ether and other routine sedimentation or concentration methods

^b^McMaster, Lumbreras rapid sedimentation, Lutz’s (sedimentation) technique, FLOTAC, Mini-FLOTAC, Polymerase chain reaction (PCR), Ritchie, Stoll’s dilution egg count

Of the 616 studies included, 138 reported separate, pooled prevalence rates for males and females. Subgroup analysis revealed pooled prevalence of 14.67% (12.61–16.86%) in males and 14.61 (12.64–16.68%) in females. A total of 71 studies reported pooled prevalence rates for different age specific groups; a subgroup analysis revealed the lowest and highest prevalence rates of 14.14% (11.67–16.80%) and 25.82% (23.59–28.12%) in people of 12–18 and ≥ 51 years of age, respectively (Table 4). In addition, 143 studies reported prevalence rates for people in urban or rural regions; a subgroup analysis revealed that people living in rural areas had a higher prevalence (17.33%, 14.21–20.69%) of *Ascaris* infection than those living in urban areas (10.02%, 7.29–13.12%) (Table 4).

### Prevalence of *Ascaris* infection in relation to diagnostic methods used and risk of bias

Various diagnostic methods were used in the studies included. We stratified these diagnostic methods into three categories: (1) the Kato-Katz method for 368 datasets (2) direct wet mount, formalin-ether and/or other routinely used sedimentation and/or concentration methods for 353 datasets; (3) other techniques, including McMaster, Lumbreras rapid sedimentation, Lutz sedimentation, flotation methods (FLOTAC and Mini-FLOTAC), Ritchie, Stoll’s dilution egg count and/or polymerase chain reaction (PCR) for 37 datasets. If more than one diagnostic test was used in a study and total positivity was recorded, we utilised this value; however, if this was not the case, we selected data relating to the diagnostic method that recorded the higher prevalence. Subgroup analysis revealed pooled prevalence rates of 12.68% (11.40–14.01%), 9.22% (8.45–10.01%), and 11.90% (7.29–17.43%) using Kato-Katz, routine parasitological methods, and other non-routine diagnostic methods, respectively (Table 4).

A critical appraisal using JBI tools showed that 378 datasets had a low risk of bias (score: 7–9/9), 269 datasets had a moderate (4–6/9), and 111 studies had a high risk of bias (≤ 3/9). Moreover, the prevalence for studies with a low, moderate and high risks of bias were 7.81% (6.94–8.73%), 14.28% (12.68–15.96%), and 16.23% (12.77–20.02%), respectively (Table 4).

### Prevalence of *Ascaris* infection in relation to socio-demographic variables

For the 758 datasets included, 153, 385, 178 and 42 datasets represented countries with low, lower middle, upper middle and high income levels, respectively. Subgroup analysis (Table 4), according to income level, revealed the highest prevalence rates of *Ascaris* infection in countries with low (13.23%, 11.64–14.91%) and lower middle (12.17%, 10.89–13.52%) income levels, with the lowest prevalence estimated for high income countries (0.88%, 0.33–1.64%). On the other hand, totals of 273, 210, 190 and 85 datasets represented countries with low, medium, high and very high HDI levels. Subgroup analysis according to HDI level, indicated that the highest and lowest prevalence rates were estimated for countries with low (12.31%, 11.01–13.67%) and very high (5.01%, 3.90–6.25%) HDI levels (Table 4). Random-effects meta-regression analyses showed a significant, decreasing trend in prevalence with increasing income (coefficient [*C*] = − 4.37e−06; *P*-value = 0.0001) and HDI (*C* = − 0.145; *P*-value = 0.0001) levels (Additional file 4: Fig. S2A, B).

### Prevalence of *Ascaris* infection over time

Another subgroup analysis was conducted to explore the prevalence of *Ascaris* infection over time. For this analysis, we stratified time into three categories: year of publication; year of the start of sampling; and year of the end of sampling. Subgroup analysis based on publication year showed prevalence rates of 9.18% (7.87–10.58%), 12.61% (11.15–14.15%), and 10.73% (9.57–11.95%) for studies published 2010–2013, 2014–2017 and 2018–2021, respectively (Table 4). Subgroup analyses according to the beginning and end dates of sampling, revealed that studies conducted between 2010 and 2015 reported the highest prevalence rates. Random-effects meta-regression analysis showed a non-significant, increasing trend in prevalence rates over time (*C* = 0.001; *P*-value = 0.4; Additional file 4: Fig. S3). Considering the start and end dates of sampling, subgroup analyses showed that studies between 2010 and 2015 reported higher prevalence rates than those performed before 2010 or between 2016 and 2021 (Table 4).

### Relationship between the prevalence of *Ascaris* infection and geographical location/climate

According to geographical parameters, the highest and lowest prevalence rates were at latitudes 0–20° (13.41%, 12.34–14.51%), and 40–60° (0.53%, 0.17–1.04%); as well as longitudes at 60–80° (16.72%, 14.23–19.36%) and 40–60° (4.55%, 3.54–5.69%), respectively (Additional file 3: Table S3). Meta-regression analyses showed significant decreasing trends in prevalence with increasing geographical latitude (*C* = − 0.003, *P*-value < 0.001), while a non-significant increasing trend was observed with increasing geographical longitudes (*C* = 0.0001, *P*-value = 0.07) (Additional file 4: Fig. S4A, B).

Considering climate parameters, the highest prevalences were in regions with mean relative humidity of ≥ 80 (i.e. 18.60%, 15.54–21.86%), mean annual precipitation of > 200 mm (15.26%, 11.04–20.01%), and a mean annual temperature of 25–29 °C (14.81%, 13.24–16.46%), while the lowest prevalences were estimated for regions with a mean relative humidity of ≤ 40 (5.61%, 4.48–6.85%), a mean annual precipitation of 0–50 mm (4.72%, 4.02–5.48%), and a mean annual temperature of 9–13 °C (0.92%, 0.51–1.43%) (Additional file 3: Table S3). There was a significant, increasing prevalence trend with increasing relative humidity (*C* = 0.002; *P*-value < 0.001), precipitation rate (*C* = 0.000; *P*-value < 0.001) and environmental temperature (*C* = − 0.0006; *P*-value < 0.001) (Additional file 4: Fig. S5A–C).

### Univariate and mulitivariate meta-regression analyses to identify source of heterogeneity

Additional file 3: Table S4 shows the results of the univariate and multivariate meta-regression analysis of study characteristics exploring the source of heterogeneity of the prevalence estimates *Ascaris* infection. As depicted in Additional file 3: Table S4, univariate analysis revealed that all covariates were significantly associated with heterogeneity of prevalence estimates, except for longitude (β, 0.05; 95% *CI:* − 0.00008–0.0005, *P*-value = 0.139), implementation year start (β, 0.05; 95% *CI:* − 0.001–0.004, *P*-value = 0.280), and implementation year start (β, 0.05; 95% *CI:* 0.03–0.06, *P*-value = 0.709). In the final multivariate meta-regression, the following variables including income level (β, 0.05; 95% *CI:* − 0.00007 to − 0.00002, *P*-value < 0.001), geographical longitude (β, 0.05; 95% *CI:* 0.0006–0.001, *P*-value < 0.001), temperature (β, 0.05; 95% *CI:* 0.001–0.006, *P*-value = 0.001), humidity (β, 0.05; 95% *CI:* 0.0003–0.002, *P*-value = 0.013), and; year of the start of sampling (β, 0.05; 95% *CI:* − 0.014 to − 0.0007, *P*-value = 0.030) remained significantly associated with heterogeneity of the prevalence of *Ascaris* infection.

## Discussion

Ascariasis continues to be an NTD of major public health significance worldwide, causing substantial morbidity in endemic regions [7]. Here, we performed a global systematic review and a meta-analysis of published studies of *Ascaris* infection in endemic regions. Our findings indicate that, globally, 11% of the ~ 6.6 billion people living in endemic regions, representing the ~ 732 million people, harbour *Ascaris*. When compared with the estimate by Pullan et al. [4], derived from data collected up to 2010, our estimates indicate a 3.5% reduction in prevalence (14.5% vs 11.0%) and a 10% reduction in the number of people with *Ascaris* infection (819 vs 730 million infected people). These findings are consistent with other recent studies [4, 32–34], reporting a reduction in the prevalence of STH infections globally. Pullan et al. [4] reported a substantial reduction in the prevalence of *Ascaris* infection in all endemic regions between 1990 (~ 32%) and 2010 (14.5%). Our findings from subgroup analyses also indicated a lower prevalence between 2016 and 2021 than between 2010 and 2015, although meta-regression analyses showed a non-significant, increasing trend over time. The likely explanation for this reduction in the prevalence of *Ascaris* infection in endemic regions is mass drug administration (MDA) within the context of control programmes [35].

Our results indicated that the prevalence of *Ascaris* infection varies among SDG regions and sub-regions, with the lowest prevalences (< 4%) in countries in Eastern Asia (China and the Republic of Korea) and Western Asia (Iraq, Iran, Palestine, Turkey and the United Arab Emirates) and the highest prevalences (> 20%) in some countries in Oceania (Papua New Guinea and the Solomon Islands), South-eastern Asia (Indonesia, Laos PDR, Malaysia, Myanmar, the Philippines, Sri Lanka and Vietnam), the Latin and Caribbean region (Brazil, Colombia, Ecuador, Honduras and Nicaragua), sub-Saharan Africa (including Angola, Burundi, Central African Republic, Chad, DR Congo and Madagascar) and South Asia (Afghanistan, Bangladesh, India and Pakistan). Lower prevalences in countries such as the Republic of Korea, China, Iran and Turkey are likely due to socio-economic improvements that have occurred over the past three decades [4, 36]. In tandem with community-based MDA, substantial improvements in personal and public hygiene (including the availability of toilets; hand washing before eating and after defaecation; consumption of washed vegetables and filtered water) have been achieved [37–39]. Improved water quality, sanitation and hygiene have been shown to be significantly associated with a lower prevalence of STHs, particularly *Ascaris* [39]. In contrast, a higher prevalence of *Ascaris* infection in South Asia, sub-Saharan Africa and Latin America might reflect less improvement in these measures. Furthermore, our results showed that the prevalence of *Ascaris* infection is higher in countries with lower levels of income and HDI.

Another explanation for the variation in prevalence of *Ascaris* infection in SDG regions is differences in environmental factors, such as temperature, humidity, rainfall, soil moisture, and contamination [40]. Environmental factors have an important impact on the embryonation of *Ascaris* eggs; consequently, egg development, viability and infectivity depend on a particular environmental temperature (≥ 25 °C) and humidity (≥ 55%) [41]. Some laboratory studies have demonstrated that higher temperature and humidity facilitates larval development, and other investigations have indicated that extreme humidity, desiccation and/or temperature can lead to compromised embryonation and larval development within eggs [41–43]. The present findings showed an increased prevalence of *Ascaris* infection in areas with higher environmental temperature, rainfall and humidity, such as in countries within South-East and Southern Asia, SubSaharan Africa and Latin America.

Another factor that may influence the observed variation in prevalence could be differential specificity and sensitivity of the diagnostic methods employed in individual studies. Diagnostic methods for the detection of *Ascaris* eggs include: direct microscopy of faecal smears, the Kato-Katz [44], McMaster [45], formol-ether concentration [46], FLOTAC [47] and mini-FLOTAC [48] techniques. The present investigation showed that 48.5% of studies included used the Kato-Katz method, as recommended by WHO. However, this latter method performs poorly when egg numbers in faecal samples are low [7, 49]. The remainder of the studies used a range of established methods. Variation in the standardisation and performance of diagnostic methods undoubtedly contributes to variability in estimates of prevalence and/or intensity of *Ascaris* infection. Molecular methods are now also being used or evaluated, including PCR-based detection of *Ascaris* DNA in faecal samples [50]. Some such approaches utilise a multiplex or a multi-parallel approach to detect and/or distinguish different parasite species [51, 52]. However, such methods are not yet in routine use around the world, as they require specialised equipment and trained personnel. Medley et al. [53] discussed some concerns about some coprodiagnostic methods employed for STHs, including *Ascaris.*

Despite the large number of studies reporting the global prevalence of *Ascaris* in humans, a major knowledge gap remains in relation to infection intensity. Only 19% of the datasets used in this systematic review contain information on infection intensity. “Intensity” was reported in a number of ways, but, most commonly, the proportion of individuals recorded to have low, moderate or high intensity was based upon EPG “cut-off”, in accord with 2002 WHO guidelines [12]. Without knowledge of infection intensity, it is challenging to assess the public health impact of a parasite such as *Ascaris* [1, 5]*.* The paucity of data on infection intensity is particularly problematic when (i) the prevalence is low; (ii) MDA is no longer implemented; and (iii) there is a need to identify high risk individuals who remain predisposed to heavy infection [6]. In tandem with the observed reduction in prevalence over time, we also observed a substantial reduction in the proportion of individuals with high intensity infection over time. The prevalence of moderate or high intensity infections is recommended as a key indicator of the success of whether STHs have been eliminated as a public health problem [54]. Therefore, this reduction of high intensity infections is of major epidemiological importance, and reflects, at least in part, the success of control strategies/programmes. Montresor et al. [55] also raised some issues regarding the implementation and effectiveness of preventative MDA in endemic regions. These authors reported that, of 96 countries endemic for ascariasis, four did not implement preventative MDA, 23 countries implemented preventative MDA without effective coverage, and 42 and 28 countries implemented MDA for < 5 and > 5 years, respectively.

An important issue that was not possible to evaluate in the present study, but should be highlighted, is the potential for cross transmission of *Ascaris* between pigs and humans (and vice versa). There has been considerable debate, over many years, as to species status of *A. lumbricoides* (humans) and *A. suum* (pigs), and whether they represent distinct species *or* operational taxonomic units that are reproductively isolated or are capable of interbreeding and producing viable offspring [17, 56]. Although their status is controversial, there is clear evidence that cross transmission of *Ascaris* occurs between pigs and humans living in close proximity, particularly in non-endemic regions [18, 57]. Some studies indicated that 4–7% of *Ascaris* from people in Guatemala and China were *A. lumbricoides* × *A. suum* ‘hybrids’ [19, 58]; other investigations have identified three main haplotype clusters (A, B and C) for *Ascaris*, employing part of the mitochondrial (mt) cytochrome *c* oxidase 1 (*cox-1*) gene (383 bp) as a marker [59–62]. There is genetic divergence between clusters: worms in clusters A and B (representing humans or pigs) were more closely related to each other, whereas worms in cluster C (from pigs or humans cross-infected with pig *Ascaris*) were genetically more distinct [16, 59, 60, 62]. It has been shown that haplotypes belonging to clusters A and B are geographically distributed worldwide in both host species (pig or human), but in different proportions [17]; the majority of worms studied in China belonged to cluster B [61], whereas most worms isolated from Uganda belonged to cluster A [59]; however, there was no clear geographical association. Moreover, important new information has recently emerged from a genome analysis of *Ascaris* worms recovered from human subjects in Kenya where pig husbandry is rare [16]. Most of these worms had mitochondrial genomes that clustered more closely to *A. suum* than to *A. lumbricoides*, suggesting that pig-*Ascaris* infection in people in Kenya might have been acquired previously from infected humans who had lived closely with pigs or non-human primates or another host animal harbouring pig-*Ascaris*, rather than from pigs [16, 63]. Phylogenetic analyses also revealed evidence to support a highly interbred *Ascaris* species genetic complex [16]. In many endemic regions, a close association between people and pigs is maintained and, therefore, may be one of the reasons for the persistence of *Ascaris* infection over time. However, it is not possible using the current coprodiagnostic methods to determine whether *Ascaris* hybrids do indeed exist.

Easton et al. [16] recommended that a One Health approach be employed for the control of human ascariasis, because pigs can serve as a reservoir for human *Ascaris* infection, also with potential implications for the spread of anthelmintic resistance. Evidence provided by these authors [16] also signals a need to utilise genome-based approaches to unravel the complexity of the enigmatic and controversial questions regarding the species status of human- and pig-*Ascaris* and host affiliation(s), and the genetic composition of *Ascaris* populations in both humans and pigs in different countries around the world.

The present study has a number of strengths. By including 616 studies and results for ~ 5 million people from 81 countries, this is one of the most comprehensive reviews on the epidemiology of *Ascaris* to date. We only included data for the ‘general population’ and excluded high risk groups, with the intent of minimising prevalence overestimates. However, this may have led to underestimates of *Ascaris* infection prevalence; therefore, our estimates might not be entirely representative of individuals in all communities. We also pooled data to highlight differences within and between different regions around the world, and assessed the impact of geographical, climatic and socio-economic factors on the prevalence of *Ascaris* infection.

However, some limitations of the present study are acknowledged. First, we included only studies listed in PubMed and Scopus; therefore we may have missed some studies, such as those published in non-indexed journals. However, we searched Google Scholar to identify additional, potentially relevant studies; nonetheless, given the large number of datasets studied, the inclusion of any potentially (unintentionally) omitted studies would not have significantly altered the overall findings. Second, as mentioned above, in some cases, our estimates might not be representative of national prevalences or of all communities in a country, particularly for those with small numbers of eligible studies. Data were not available for many countries, and there was insufficient data for some important endemic regions, including Central Asia, sub-Saharan Africa and Oceania. Moreover, estimates for countries such as Malaysia and Indonesia might not be truly representative of their whole population, because the majority of studies conducted in these countries focused on high-risk regions or communities. Although, in the present study, we included only studies published after 2010, in a substantial number of studies, sampling dates were before 2010 (see Table 4); therefore, as a result of changes in social and environmental conditions over time, the prevalence estimates from earlier studies may not have been entirely reflective of the infection status at the time. Third, few studies reported data on infection intensity or age/sex-specific prevalences. A fourth limitation is the existence of high heterogeneity recorded across all regions and sub-groupings, which is expected in global prevalence estimates across time periods and locations [64–66]; the sources of this heterogeneity are explained in our results.

## Conclusions

The present findings on the prevalence and intensity of *Ascaris* infection should assist health policy makers in designing and supporting ascariasis intervention/control programmes that improve public health and reduce the burden of infection and disease. Our findings indicate that, despite a significant reduction in prevalence and intensity of *Ascaris* infection over past decades, a substantial portion of the world’s human population (i.e. ~ 732 million) is still infected with *Ascaris*, calling for enhanced efforts. Furthermore, despite its central epidemiological importance, many routine surveys fail to assess the intensity of *Ascaris* infection. This study calls for continued global efforts to control and prevent human ascariasis, to work toward achieving key SDGs by reducing the burden of STHs and increasing human health and wellbeing.

## Supplementary Information

**Additional file 1: Table S1.** Main characteristics of the included studies.

**Additional file 2: Table S2.** The main characteristics of studies that included intensity of *Ascaris* infection.

**Additional file 3: Table S3.** Prevalence estimates for *Ascaris* infection based on sub-groups according to different geographic and climate parameters, calculated using a random effects model. **Table S4.** Association between study variables and heterogeneity of *Ascariasis* prevalence estimates.

**Additional file 4: Figure S1.** Search strategy in databases. **Figure S2.** Random-effects meta-regression analyses of the prevalence of *Ascaris* infection in general population according to (A) a country's income level, showing a statistically significant downward trend in prevalence in countries with higher income levels; (B) human development index (HDI), showing a statistically significant downward trend in prevalence in countries with higher HDIs. **Figure S3.** Random-effects meta-regression analyses of the prevalence of *Ascaris* infection in the general human population, according to publication year, showing a statistically non-significant upward trend in prevalence in recent years. **Figure S4.** Random-effects meta-regression analyses of the prevalence of *Ascaris* infection in the general human population, according to (A) geographical latitude, showing a statistically significant downward trend in prevalence with increasing geographical latitude; and (B) geographical longitude, showing a statistically non-significant upward trend in prevalence with increasing geographical longitude. **Figure S5.** Random-effects meta-regression analyses of the prevalence of *Ascaris* infection in the general human population, according to (A) the mean annual relative humidity, showing a statistically significant upward trend in prevalence with increasing humidity; and (B) the mean annual precipitation rate, showing a statistically significant upward trend in prevalence with increasing precipitation; (C), the mean annual temperature, showing a statistically significant upward trend in prevalence with increasing environmental temperature.

**Additional file 5: Checklist S1.** PRISMA checklist.

## Data Availability

The all data are presented in the manuscript and Additional files.

## Abbreviations

NTDs: Neglected tropical diseases
STH: Soil-transmitted helminth
EPG: Eggs per gram
WHO: World Health Organization
PRISMA: Preferred Reporting Items for Systematic Reviews and Meta-Analyses
SDG: Sustainable development goal
JBI: Joanna Briggs Institute
REM: Random-effects model
*CI*s: Confidence intervals
HDI: Human development index
PCR: Polymerase chain reaction
