# Supplementary material for: Global prevalence of Ascaris infection in humans (2010–2021): a systematic review and meta-analysis
Source: Infect Dis Poverty. 2022 Nov 18;11:113. doi: 10.1186/s40249-022-01038-z (PMC9673379; doi:10.1186/s40249-022-01038-z)
Supplement: Supplementary file 1 — Additional file 1: Table S1. Main characteristics of the included studies. [file 40249_2022_1038_MOESM1_ESM.docx]

**Table S1.** Main characteristics of the included studies.

| First author's last name/ Pub year (Ref) | Type of population | Country | Diagnostic Method | Human Development Index | Level of Income | Sample size | Infected Total | Risk of bias |
| --- | --- | --- | --- | --- | --- | --- | --- | --- |
| Central and Southern Asia |  |  |  |  |  |  |  |  |
| Ragunathan et al. (2010)^1^ | Children | India | Routine parasitological methods* | Medium | Lower-middle | 1172 | 205 | Low |
| Rayan et al. (2010)^2^ | Children | India | Routine parasitological methods* | Medium | Lower-middle | 195 | 2 | High |
| Steinmann et al. (2010)^3^ | Children | Kyrgyzstan | Kato-Katz | Medium | Lower-middle | 1262 | 292 | Low |
| Kahanal et al. (2011)^4^ | Children | Nepal | Routine parasitological methods* | Medium | Lower-middle | 142 | 28 | High |
| Marothi et al. (2011)^5^ | Population based | India | Routine parasitological methods* | Medium | Lower-middle | 5990 | 173 | Moderate |
| Taheri et al. (2011)^6^ | Children | Iran | Routine parasitological methods* | High | Lower-middle | 2169 | 12 | Low |
| Gunawardena et al. (2011)^7^ | Children | Sri Lanka | Kato-Katz | High | Lower-middle | 341 | 105 | Moderate |
| Gunawardena et al. (2011)^7^ | Children | Sri Lanka | Kato-Katz | High | Lower-middle | 377 | 43 | Moderate |
| Gunawardena et al. (2011)^7^ | Children | Sri Lanka | Kato-Katz | High | Lower-middle | 377 | 139 | Moderate |
| Gunawardena et al. (2011)^7^ | Children | Sri Lanka | Kato-Katz | High | Lower-middle | 388 | 88 | Moderate |
| Gunawardena et al. (2011)^7^ | Children | Sri Lanka | Kato-Katz | High | Lower-middle | 407 | 87 | Moderate |
| Matthys et al. (2011)^8^ | Children | Tajikistan | Kato-Katz | Medium | Lower-middle | 594 | 26 | Low |
| Choubisa et al. (2012)^9^ | Population based | India | Routine parasitological methods* | Medium | Lower-middle | 224 | 10 | Moderate |
| Parameshwarappa et al. (2012)^10^ | Population based | India | Routine parasitological methods* | Medium | Lower-middle | 1000 | 35 | Moderate |
| Shrestha et al. (2012)^11^ | Children | Nepal | Routine parasitological methods* | Medium | Lower-middle | 260 | 6 | Moderate |
| Ashok et al. (2013)^12^ | Children | India | Routine parasitological methods* | Medium | Lower-middle | 208 | 6 | Moderate |
| Kaliappan et al. (2013)^13^ | Population based | India | Routine parasitological methods* | Medium | Lower-middle | 1237 | 19 | Low |
| Shobha et al. (2013)^14^ | Population based | India | Routine parasitological methods* | Medium | Lower-middle | 880 | 18 | Low |
| Shrestha et al. (2013)^15^ | Children | Nepal | Routine parasitological methods* | Medium | Lower-middle | 495 | 112 | Moderate |
| Tiwari et al. (2013)^16^ | Children | Nepal | Routine parasitological methods* | Medium | Lower-middle | 530 | 31 | Low |
| Ranjan et al. (2013)^17^ | Children | India | Kato-Katz | Medium | Lower-middle | 347 | 28 | Moderate |
| Kaliappan et al. (2013)^13^ | Population based | India | Routine parasitological methods* | Medium | Lower-middle | 1237 | 19 | Low |
| Sherkhonov et al. (2013)^18^ | Children | Tajikistan | Kato-Katz | Medium | Lower-middle | 12,642 | 277 | Low |
| Dhanabal et al. (2014)^19^ | Population based | India | Routine parasitological methods* | Medium | Lower-middle | 256 | 16 | Moderate |
| Abdi et al. (2014)^20^ | Children | Iran | Routine parasitological methods* | High | Lower-middle | 650 | 25 | Low |
| Bilakshan et al. (2014)^21^ | Children | Nepal | Routine parasitological methods* | Medium | Lower-middle | 935 | 18 | Low |
| Jafari et al. (2014)^22^ | Population based | Iran | Routine parasitological methods* | High | Lower-middle | 652 | 0 | Low |
| Dhanabal et al. (2014)^19^ | Population based | India | Routine parasitological methods* | Medium | Lower-middle | 256 | 16 | Moderate |
| Ullah et al. (2014)^23^ | Children | Pakistan | Routine parasitological methods* | Medium | Lower-middle | 222 | 121 | Moderate |
| Ahmed et al. (2015)^24^ | Children | Pakistan | Routine parasitological methods* | Medium | Lower-middle | 150 | 27 | High |
| Bhattachan et al. (2015)^25^ | Children | Nepal | Routine parasitological methods* | Medium | Lower-middle | 296 | 33 | Moderate |
| Hosseini et al. (2015)^26^ | Population based | Iran | Routine parasitological methods* | High | Lower-middle | 1025 | 0 | Low |
| Korzeniewski et al. (2015)^27^ | Children | Afghanistan | Routine parasitological methods* | Low | Low | 1369 | 295 | Low |
| Pandey et al. (2015)^28^ | Children | Nepal | Routine parasitological methods* | Medium | Lower-middle | 300 | 3 | Moderate |
| Sadeghi et al. (2015)^29^ | Population based | Iran | Routine parasitological methods* | High | Lower-middle | 5739 | 0 | Low |
| Supram et al. (2015)^30^ | Population based | Nepal | Routine parasitological methods* | Medium | Lower-middle | 9470 | 22 | Low |
| Uppal et al. (2015)^31^ | Population based | India | Routine parasitological methods* | Medium | Lower-middle | 6527 | 75 | Low |
| Benjamin et al. (2015)^32^ | Population based | Bangladesh | Other methods¶ | Medium | Lower-middle | 1630 | 214 | Low |
| Attaullah et al. (2016)^33^ | Children | Pakistan | Routine parasitological methods* | Medium | Lower-middle | 253 | 23 | Moderate |
| Barkhori et al. (2016)^34^ | Population based | Iran | Routine parasitological methods* | High | Lower-middle | 1060 | 2 | Low |
| Bilakshan et al. (2016)^35^ | Children | Nepal | Routine parasitological methods* | Medium | Lower-middle | 200 | 11 | Moderate |
| Khan et al. (2019)^36^ | Children | Pakistan | Routine parasitological methods* | Medium | Lower-middle | 300 | 125 | Moderate |
| Korzeniewski et al. (2016)^37^ | Children | Afghanistan | Routine parasitological methods* | Low | Low | 500 | 101 | Low |
| Wani et al. (2016)^38^ | Children | India | Routine parasitological methods* | Medium | Lower-middle | 352 | 253 | Moderate |
| Dahal et al. (2017)^39^ | Children | Nepal | Routine parasitological methods* | Medium | Lower-middle | 588 | 1 | Low |
| Korzeniewski et al. (2017)^40^ | Population based | Afghanistan | Routine parasitological methods* | Low | Low | 548 | 107 | Low |
| Mubarak et al. (2017)^41^ | Population based | Afghanistan | Routine parasitological methods* | Low | Low | 520 | 82 | Moderate |
| Praharaj et al. (2017)^42^ | Population based | India | Routine parasitological methods* | Medium | Lower-middle | 257,588 | 2565 | Low |
| Saki et al. (2017)^43^ | Population based | Iran | Routine parasitological methods* | High | Lower-middle | 13,698 | 0 | Low |
| Turki et al. (2017)^44^ | Children | Iran | Routine parasitological methods* | High | Lower-middle | 1465 | 0 | Moderate |
| Yadav et al. (2017)^45^ | Children | Nepal | Routine parasitological methods* | Medium | Lower-middle | 3000 | 1273 | Low |
| Galgamuwa et al. (2016)^46^ | Children | Sri Lanka | Kato-Katz | High | Lower-middle | 258 | 98 | Moderate |
| Ganguly et al. (2017)^47^ | Children | India | Kato-Katz | Medium | Lower-middle | 154 | 136 | Low |
| Ganguly et al. (2017)^47^ | Children | India | Kato-Katz | Medium | Lower-middle | 290 | 244 | Low |
| Ganguly et al. (2017)^47^ | Children | India | Kato-Katz | Medium | Lower-middle | 299 | 65 | Low |
| Ganguly et al. (2017)^47^ | Children | India | Kato-Katz | Medium | Lower-middle | 526 | 27 | Low |
| Ganguly et al. (2017)^47^ | Children | India | Kato-Katz | Medium | Lower-middle | 542 | 10 | Low |
| Ganguly et al. (2017)^47^ | Children | India | Kato-Katz | Medium | Lower-middle | 752 | 546 | Low |
| Ganguly et al. (2017)^47^ | Children | India | Kato-Katz | Medium | Lower-middle | 1031 | 860 | Low |
| Ganguly et al. (2017)^47^ | Children | India | Kato-Katz | Medium | Lower-middle | 1167 | 989 | Low |
| Ganguly et al. (2017)^47^ | Children | India | Kato-Katz | Medium | Lower-middle | 1660 | 1257 | Low |
| Galgamuwa et al. (2018)^48^ | Children | Sri Lanka | Routine parasitological methods* | High | Lower-middle | 489 | 188 | Low |
| Mareeswaran et al. (2018)^49^ | Population based | India | Routine parasitological methods* | Medium | Lower-middle | 390 | 10 | Moderate |
| Norbu et al. (2018)^50^ | Population based | Bhutan | Routine parasitological methods* | Medium | Lower-middle | 5919 | 8 | Moderate |
| Lepper et al. (2018)^51^ | Population based | Sri Lanka | Kato-Katz | High | Lower-middle | 477 | 251 | Moderate |
| Arshad et al. (2019)^52^ | Population based | Pakistan | Routine parasitological methods* | Medium | Lower-middle | 2212 | 15 | Moderate |
| Barki et al. (2019)^53^ | Children | Pakistan | Routine parasitological methods* | Medium | Lower-middle | 360 | 101 | Moderate |
| Langbang et al. (2019)^54^ | Population based | India | Routine parasitological methods* | Medium | Lower-middle | 1006 | 5 | Moderate |
| Shrestha et al. (2019)^55^ | Children | Nepal | Routine parasitological methods* | Medium | Lower-middle | 284 | 62 | Moderate |
| Valiram et al. (2019)^56^ | Children | Pakistan | Routine parasitological methods* | Medium | Lower-middle | 90 | 45 | High |
| Safi et al. (2019)^57^ | Children | Afghanistan | Kato-Katz | Low | Low | 2263 | 582 | Low |
| Afshar et al. (2020)^58^ | Population based | Iran | Routine parasitological methods* | High | Lower-middle | 861 | 3 | Moderate |
| Gupta et al. (2020)^59^ | Children | Nepal | Routine parasitological methods* | Medium | Lower-middle | 285 | 5 | Low |
| Khadka et al. (2020)^60^ | Population based | Nepal | Routine parasitological methods* | Medium | Lower-middle | 205 | 32 | Low |
| Mahmoudvand et al. (2020)^61^ | Children | Iran | Routine parasitological methods* | High | Lower-middle | 366 | 2 | Moderate |
| Subba et al. (2020)^62^ | Children | India | Routine parasitological methods* | Medium | Lower-middle | 300 | 9 | Moderate |
| Benjamin-Chung et al. (2020)^63^ | Children | Bangladesh | Kato-Katz | Medium | Lower-middle | 2799 | 1035 | Low |
| Davlin et al. (2020)^64^ | Population based | Bangladesh | Kato-Katz | Medium | Lower-middle | 7164 | 352 | Moderate |
| Dukpa et al. (2020)^65^ | Children | Bhutan | Other methods¶ | Medium | Lower-middle | 474 | 0 | Moderate |
| Dukpa et al. (2020)^65^ | Children | Bhutan | Other methods¶ | Medium | Lower-middle | 484 | 9 | Moderate |
| Afshar  et al. (2020)^58^ | Population based | Iran | Routine parasitological methods* | High | Lower-middle | 861 | 3 | Low |
| Dukpa et al. (2020)^65^ | Children | Bhutan | Other methods¶ | Medium | Lower-middle | 498 | 2 | Moderate |
| Afridi et al. (2021)^66^ | Children | Pakistan | Routine parasitological methods* | Medium | Lower-middle | 300 | 67 | Moderate |
| Irum et al. (2021)^67^ | Population based | Pakistan | Routine parasitological methods* | Medium | Lower-middle | 318 | 125 | Moderate |
| Khan et al. (2021)^68^ | Children | Pakistan | Routine parasitological methods* | Medium | Lower-middle | 184 | 82 | Moderate |
| Rahimi et al. (2021)^69^ | children | Afghanistan | Routine parasitological methods* | Low | Low | 1426 | 267 | Moderate |
| Rahman et al. (2021)^70^ | Children | Pakistan | Routine parasitological methods* | Medium | Lower-middle | 400 | 95 | Low |
| Shrestha et al. (2021)^71^ | Children | Nepal | Routine parasitological methods* | Medium | Lower-middle | 400 | 22 | Low |
| Thapa et al. (2021)^72^ | Population based | Nepal | Routine parasitological methods* | Medium | Lower-middle | 498 | 53 | Moderate |
| Ulaganeethi et al. (2021)^73^ | Population based | India | Routine parasitological methods* | Medium | Lower-middle | 3267 | 53 | Low |
| Ulhaq et al. (2021)^74^ | Children | Pakistan | Routine parasitological methods* | Medium | Lower-middle | 324 | 187 | Low |
| Ajjampur et al. (2021)^75^ | Population based | India | Kato-Katz | Medium | Lower-middle | 6089 | 6 | Low |
| Eastern and South-Eastern Asia |  |  |  |  |  |  |  |  |
| Kim et al. (2010)^76^ | Population based | Korea | Routine parasitological methods* | Very high | High | 695 | 0 | Low |
| Kim et al. (2010)^76^ | Population based | Korea | Routine parasitological methods* | Very high | High | 836 | 0 | Low |
| Kim et al. (2010)^76^ | Population based | Korea | Routine parasitological methods* | Very high | High | 868 | 0 | Low |
| Kim et al. (2010)^76^ | Population based | Korea | Routine parasitological methods* | Very high | High | 880 | 0 | Low |
| Kim et al. (2010)^76^ | Population based | Korea | Routine parasitological methods* | Very high | High | 914 | 0 | Low |
| Kim et al. (2010)^76^ | Population based | Korea | Routine parasitological methods* | Very high | High | 922 | 0 | Low |
| Kim et al. (2010)^76^ | Population based | Korea | Routine parasitological methods* | Very high | High | 936 | 0 | Low |
| Kim et al. (2010)^76^ | Population based | Korea | Routine parasitological methods* | Very high | High | 953 | 0 | Low |
| Kim et al. (2010)^76^ | Population based | Korea | Routine parasitological methods* | Very high | High | 960 | 2 | Low |
| Kim et al. (2010)^76^ | Population based | Korea | Routine parasitological methods* | Very high | High | 988 | 1 | Low |
| Kim et al. (2010)^76^ | Population based | Korea | Routine parasitological methods* | Very high | High | 1001 | 0 | Low |
| Kim et al. (2010)^76^ | Population based | Korea | Routine parasitological methods* | Very high | High | 1002 | 0 | Low |
| Kim et al. (2010)^76^ | Population based | Korea | Routine parasitological methods* | Very high | High | 1009 | 0 | Low |
| Kim et al. (2010)^76^ | Population based | Korea | Routine parasitological methods* | Very high | High | 1029 | 0 | Low |
| Kim et al. (2010)^76^ | Population based | Korea | Routine parasitological methods* | Very high | High | 1038 | 0 | Low |
| Kim et al. (2010)^76^ | Population based | Korea | Routine parasitological methods* | Very high | High | 1049 | 0 | Low |
| Kim et al. (2010)^76^ | Population based | Korea | Routine parasitological methods* | Very high | High | 1056 | 0 | Low |
| Kim et al. (2010)^76^ | Population based | Korea | Routine parasitological methods* | Very high | High | 1072 | 2 | Low |
| Kim et al. (2010)^76^ | Population based | Korea | Routine parasitological methods* | Very high | High | 1076 | 1 | Low |
| Kim et al. (2010)^76^ | Population based | Korea | Routine parasitological methods* | Very high | High | 1098 | 0 | Low |
| Kim et al. (2010)^76^ | Population based | Korea | Routine parasitological methods* | Very high | High | 1120 | 0 | Low |
| Kim et al. (2010)^76^ | Population based | Korea | Routine parasitological methods* | Very high | High | 1163 | 0 | Low |
| Kim et al. (2010)^76^ | Population based | Korea | Routine parasitological methods* | Very high | High | 1165 | 0 | Low |
| Kim et al. (2010)^76^ | Population based | Korea | Routine parasitological methods* | Very high | High | 1172 | 4 | Low |
| Kim et al. (2010)^76^ | Population based | Korea | Routine parasitological methods* | Very high | High | 1175 | 0 | Low |
| Kim et al. (2010)^76^ | Population based | Korea | Routine parasitological methods* | Very high | High | 1193 | 0 | Low |
| Kim et al. (2010)^76^ | Population based | Korea | Routine parasitological methods* | Very high | High | 1213 | 0 | Low |
| Kim et al. (2010)^76^ | Population based | Korea | Routine parasitological methods* | Very high | High | 1256 | 0 | Low |
| Kim et al. (2010)^76^ | Population based | Korea | Routine parasitological methods* | Very high | High | 2424 | 0 | Low |
| Manabo et al. (2010)^77^ | Children | Philippines | Kato-Katz | High | Lower-middle | 102 | 33 | High |
| Ziegelbauer et al. (2010)^78^ | Children | China | Kato-Katz | High | Upper-middle | 268 | 125 | Moderate |
| Ngiu et al. (2011)^79^ | Population based | Malaysia | Routine parasitological methods* | Very high | Upper-middle | 716 | 276 | Low |
| Balen et al. (2011)^80^ | Population based | China | Kato-Katz | High | Upper-middle | 595 | 13 | Moderate |
| Balen et al. (2011)^80^ | Population based | China | Kato-Katz | High | Upper-middle | 703 | 58 | Moderate |
| Belizario et al. (2011)^81^ | Children | Philippines | Kato-Katz | High | Lower-middle | 572 | 101 | Low |
| Kounnavong et al. (2011)^82^ | Children | Laos PDR | Kato-Katz | Medium | Lower-middle | 570 | 156 | Low |
| Sayasone et al. (2011)^83^ | Population based | Laos PDR | Kato-Katz | Medium | Lower-middle | 212 | 182 | Moderate |
| Sayasone et al. (2011)^83^ | Population based | Laos PDR | Kato-Katz | Medium | Lower-middle | 225 | 16 | Moderate |
| Sayasone et al. (2011)^83^ | Population based | Laos PDR | Kato-Katz | Medium | Lower-middle | 232 | 14 | Moderate |
| Conlan et al. (2012)^84^ | Population based | Laos PDR | Routine parasitological methods* | Medium | Lower-middle | 306 | 22 | Moderate |
| Conlan et al. (2012)^84^ | Population based | Laos PDR | Routine parasitological methods* | Medium | Lower-middle | 316 | 95 | Moderate |
| Conlan et al. (2012)^84^ | Population based | Laos PDR | Routine parasitological methods* | Medium | Lower-middle | 352 | 143 | Moderate |
| Conlan et al. (2012)^84^ | Population based | Laos PDR | Routine parasitological methods* | Medium | Lower-middle | 384 | 94 | Moderate |
| Huat et al. (2012)^85^ | Children | Malaysia | Routine parasitological methods* | Very high | Upper-middle | 79 | 16 | High |
| Sinniah et al. (2012)^86^ | Population based | Malaysia | Routine parasitological methods* | Very high | Upper-middle | 77 | 23 | High |
| Ahmed et al. (2012)^87^ | Children | Malaysia | Kato-Katz | Very high | Upper-middle | 254 | 121 | High |
| Ezeamama et al. (2012)^88^ | Population based | Philippines | Kato-Katz | High | Lower-middle | 253 | 202 | Moderate |
| Jiang et al. (2012)^89^ | Population based | China | Kato-Katz | High | Upper-middle | 1019 | 308 | Low |
| Ngui et al. (2012)^90^ | Children | Malaysia | Kato-Katz | Very high | Upper-middle | 550 | 229 | Low |
| Tian et al. (2012)^91^ | Population based | China | Kato-Katz | High | Upper-middle | 303 | 2 | Low |
| Wang et al. (2012)^92^ | Children | China | Kato-Katz | High | Upper-middle | 821 | 254 | Low |
| Wang et al. (2012)^92^ | Children | China | Kato-Katz | High | Upper-middle | 880 | 31 | Low |
| Wu et al. (2012)^93^ | Population based | China | Kato-Katz | High | Upper-middle | 29,473 | 24 | Low |
| Yap et al. (2012)^94^ | Children | China | Kato-Katz | High | Upper-middle | 69 | 30 | High |
| Yong et al. (2012)^95^ | Population based | Cambodia | Kato-Katz | Medium | Lower-middle | 1799 | 11 | Low |
| Kamel Abd Ghani et al. (2013)^96^ | Children | Malaysia | Routine parasitological methods* | Very high | Upper-middle | 272 | 124 | High |
| Prownebon et al. (2013)^97^ | Children | Thailand | Routine parasitological methods* | High | Upper-middle | 282 | 90 | High |
| Chen et al. (2013)^98^ | Children | China | Kato-Katz | High | Upper-middle | 45,645 | 82 | Low |
| Gan et al. (2013)^99^ | Children | China | Kato-Katz | High | Upper-middle | 407 | 7 | Moderate |
| Gan et al. (2013)^99^ | Population based | China | Kato-Katz | High | Upper-middle | 410 | 21 | Moderate |
| Liu et al. (2013)^100^ | Children | China | Kato-Katz | High | Upper-middle | 2121 | 8 | Low |
| Pham-Duc et al. (2013)^101^ | Population based | Viet Nam | Kato-Katz | High | Lower-middle | 1425 | 342 | Low |
| Phongluxa et al. (2013)^102^ | Population based | Laos PDR | Kato-Katz | Medium | Lower-middle | 574 | 56 | Low |
| Wu et al. (2013)^103^ | Population based | China | Kato-Katz | High | Upper-middle | 2002 | 3 | Low |
| Al-Delaimy et al. (2014)^104^ | Children | Malaysia | Routine parasitological methods* | Very high | Upper-middle | 498 | 238 | Moderate |
| Al-Mekhlafi et al. (2014)^105^ | Children | Malaysia | Routine parasitological methods* | Very high | Upper-middle | 250 | 239 | Moderate |
| Anuar et al. (2014)^106^ | Population based | Malaysia | Routine parasitological methods* | Very high | upper-middle | 500 | 119 | Moderate |
| Sinniah et al. (2014)^107^ | Children | Malaysia | Routine parasitological methods* | Very high | Upper-middle | 342 | 36 | High |
| Du et al. (2014)^108^ | Population based | China | Kato-Katz | High | Upper-middle | 289 | 154 | Moderate |
| Eom et al. (2014)^109^ | Population based | Laos PDR | Kato-Katz | Medium | Lower-middle | 6178 | 227 | Low |
| Khieu et al. (2014)^110^ | Population based | Cambodia | Kato-Katz | Medium | Lower-middle | 2861 | 23 | Low |
| Laymanivong et al. (2014)^111^ | Population based | Laos PDR | Kato-Katz | Medium | Lower-middle | 8610 | 999 | Low |
| Papier et al. (2014)^112^ | Children | Philippines | Kato-Katz | High | Lower-middle | 693 | 363 | Low |
| Ruankham et al. (2014)^113^ | Population based | Thailand | Kato-Katz | High | Upper-middle | 263 | 12 | Moderate |
| Wang et al. (2014)^114^ | Population based | China | Kato-Katz | High | Upper-middle | 6163 | 38 | Low |
| Yang et al. (2014)^115^ | Children | China | Kato-Katz | High | Upper-middle | 1134 | 106 | Low |
| Schär et al. (2014)^116^ | Population based | Cambodia | Other methods¶ | Medium | Lower-middle | 218 | 5 | Moderate |
| Kaewpitoon et al. (2015)^117^ | Children | Laos PDR | Routine parasitological methods* | High | Lower-middle | 1957 | 72 | Low |
| Ngui et al. (2015)^118^ | Population based | Malaysia | Routine parasitological methods* | Very high | Upper-middle | 634 | 170 | Moderate |
| Sungkar et al. (2015)^119^ | Population based | Indonesia | Routine parasitological methods* | High | Lower-middle | 424 | 279 | Moderate |
| Zhao et al. (2015)^120^ | Children | Myanmar | Routine parasitological methods* | Medium | Lower-middle | 138 | 90 | High |
| Chai et al. (2015)^121^ | Population based | Laos PDR | Kato-Katz | Medium | Lower-middle | 108 | 7 | Moderate |
| Chai et al. (2015)^121^ | Population based | Laos PDR | Kato-Katz | Medium | Lower-middle | 172 | 3 | Moderate |
| Chai et al. (2015)^121^ | Population based | Laos PDR | Kato-Katz | Medium | Lower-middle | 172 | 16 | Moderate |
| Chai et al. (2015)^121^ | Population based | Laos PDR | Kato-Katz | Medium | Lower-middle | 191 | 10 | Moderate |
| Liu et al. (2015)^122^ | Children | China | Kato-Katz | High | Upper-middle | 2179 | 7 | Low |
| Ross et al. (2016)^123^ | Population based | Philippines | Kato-Katz | High | Lower-middle | 6976 | 2546 | Low |
| Sayasone et al. (2015)^124^ | Population based | Laos PDR | Kato-Katz | Medium | Lower-middle | 485 | 162 | Moderate |
| Sayasone et al. (2015)^125^ | Children | Laos PDR | Kato-Katz | Medium | Lower-middle | 1313 | 221 | Low |
| Soares Magalhães et al. (2015)^126^ | Population based | Philippines | Kato-Katz | High | Lower-middle | 35,573 | 9859 | Low |
| Vonghachack et al. (2015)^127^ | Population based | Laos PDR | Kato-Katz | Medium | Lower-middle | 729 | 2 | Low |
| Xiao et al. (2015)^128^ | Population based | China | Kato-Katz | High | Upper-middle | 438 | 59 | Low |
| Yong et al. (2014)^129^ | Population based | Cambodia | Kato-Katz | Medium | Lower-middle | 32,201 | 1478 | Low |
| Gordon et al. (2015)^130^ | Population based | Philippines | Other methods¶ | High | Lower-middle | 545 | 317 | Low |
| Chin et al. (2016)^131^ | Population based | Malaysia | Routine parasitological methods* | Very high | Upper-middle | 80 | 17 | High |
| Chin et al. (2016)^131^ | Population based | Malaysia | Routine parasitological methods* | Very high | Upper-middle | 106 | 8 | High |
| Nithyamathi et al. (2016)^132^ | Children | Malaysia | Routine parasitological methods* | Very high | Upper-middle | 1760 | 26 | Low |
| Rajoo et al. (2017)^133^ | Population based | Malaysia | Routine parasitological methods* | Very high | Upper-middle | 341 | 83 | Moderate |
| de Gier et al. (2016)^134^ | Children | Viet Nam | Kato-Katz | High | Lower-middle | 510 | 327 | Low |
| Fang-Wei et al. (2016)^135^ | Population based | China | Kato-Katz | High | Upper-middle | 1002 | 17 | Low |
| Hung et al. (2016)^136^ | Population based | Viet Nam | Kato-Katz | High | Lower-middle | 1206 | 24 | Low |
| Laymanivong et al. (2016)^137^ | Population based | Laos PDR | Kato-Katz | Medium | Lower-middle | 327 | 5 | Moderate |
| Campbell et al. (2016)^138^ | Population based | Timor-Leste | Other methods¶ | Medium | Lower-middle | 2152 | 516 | Low |
| Llewellyn et al. (2016)^139^ | Population based | Timor-Leste | Other methods¶ | Medium | Lower-middle | 680 | 287 | Low |
| He et al. (2017)^140^ | Population based | China | Routine parasitological methods* | High | Upper-middle | 98,804 | 329 | Low |
| Kitvatanachai et al. (2017)^141^ | Population based | Thailand | Routine parasitological methods* | High | Upper-middle | 221 | 3 | Moderate |
| Lee et al. (2017)^142^ | Population based | China | Routine parasitological methods* | High | Upper-middle | 8396 | 68 | Low |
| Liao et al. (2017)^143^ | Children | Cambodia | Routine parasitological methods* | Medium | Lower-middle | 308 | 0 | Moderate |
| Nanthavong et al. (2017)^144^ | Children | Laos PDR | Routine parasitological methods* | Medium | Lower-middle | 74 | 24 | High |
| Punsawad et al. (2017)^145^ | Population based | Thailand | Routine parasitological methods* | High | Upper-middle | 324 | 0 | High |
| Ribas et al. (2017)^146^ | Population based | Laos PDR | Routine parasitological methods* | Medium | Lower-middle | 305 | 13 | Moderate |
| Darlan et al. (2017)^147^ | Children | Indonesia | Kato-Katz | High | Lower-middle | 80 | 20 | High |
| Darlan et al. (2017)^148^ | Children | Indonesia | Kato-Katz | High | Lower-middle | 132 | 4 | High |
| Dunn et al. (2017)^149^ | Population based | Myanmar | Kato-Katz | Medium | Lower-middle | 712 | 38 | Low |
| Liu et al. (2017)^150^ | Children | China | Kato-Katz | High | Upper-middle | 4985 | 1151 | Low |
| Liwanag et al. (2017)^151^ | Population based | Philippines | Kato-Katz | High | Lower-middle | 951 | 292 | Low |
| Vonghachack et al. (2017)^152^ | Population based | Laos PDR | Kato-Katz | Medium | Lower-middle | 377 | 1 | Moderate |
| Vonghachack et al. (2017)^153^ | Population based | Laos PDR | Kato-Katz | Medium | Lower-middle | 994 | 6 | Low |
| Yu et al. (2017)^154^ | Population based | Philippines | Kato-Katz | High | Lower-middle | 10,434 | 1074 | Low |
| Zhang et al. (2017)^155^ | Population based | China | Kato-Katz | High | Upper-middle | 6170 | 21 | Low |
| Mationg et al. (2017)^156^ | Children | Philippines | Other methods¶ | High | Lower-middle | 263 | 178 | High |
| Li et al. (2018)^157^ | Population based | China | Routine parasitological methods* | High | Upper-middle | 22,263 | 267 | Low |
| Yang et al. (2018)^158^ | Children | China | Kato-Katz | High | Upper-middle | 321 | 33 | High |
| Mohd-Shaharuddin et al. (2018)^159^ | Population based | Malaysia | Routine parasitological methods* | Very high | Upper-middle | 411 | 187 | Moderate |
| Suntaravitun et al. (2018)^160^ | Population based | Thailand | Routine parasitological methods* | High | Upper-middle | 224 | 3 | High |
| Xiong et al. (2018)^161^ | Population based | China | Routine parasitological methods* | High | Upper-middle | 236 | 0 | High |
| Yanola et al. (2018)^162^ | Children | Thailand | Routine parasitological methods* | High | Upper-middle | 375 | 49 | Moderate |
| de Gier et al. (2018)^163^ | Children | Cambodia | Kato-Katz | Medium | Lower-middle | 1795 | 5 | Low |
| Jiang et al. (2018)^164^ | Population based | China | Kato-Katz | High | Upper-middle | 1023 | 0 | Low |
| Yang et al. (2018)^158^ | children | China | Kato-Katz | High | Upper-middle | 321 | 32 | High |
| Li et al. (2019)^165^ | Children | China | Routine parasitological methods* | High | Upper-middle | 1473 | 110 | Low |
| Ansari Nasution et al. (2019)^166^ | Children | Indonesia | Kato-Katz | High | Lower-middle | 56 | 33 | High |
| Chard et al. (2019)^167^ | Population based | Laos PDR | Kato-Katz | Medium | Lower-middle | 746 | 61 | Moderate |
| Dai et al. (2019)^168^ | Population based | China | Kato-Katz | High | Upper-middle | 30,153 | 35 | Low |
| Han et al. (2019)^169^ | Population based | Myanmar | Kato-Katz | Medium | Lower-middle | 698 | 269 | Low |
| JingXiao et al. (2019)^170^ | Population based | China | Kato-Katz | High | Upper-middle | 12,859 | 138 | Low |
| Muslim et al. (2019)^171^ | Population based | Malaysia | Kato-Katz | Very high | Upper-middle | 416 | 180 | Moderate |
| Nasution et al. (2019)^166^ | Children | Indonesia | Kato-Katz | High | Lower-middle | 242 | 169 | High |
| Adli et al. (2020)^172^ | Children | Malaysia | Routine parasitological methods* | Very high | Upper-middle | 92 | 35 | High |
| Kurscheid et al. (2020)^173^ | Population based | Indonesia | Routine parasitological methods* | High | Lower-middle | 6444 | 1679 | Low |
| Lim-Leroy et al. (2020)^174^ | Children | Malaysia | Routine parasitological methods* | Very high | upper-middle | 407 | 31 | Moderate |
| Nasr et al. (2020)^175^ | Children | Malaysia | Routine parasitological methods* | Very high | Upper-middle | 1142 | 721 | Low |
| Nur Adli et al. (2020)^172^ | Children | Malaysia | Routine parasitological methods* | Very high | Upper-middle | 92 | 35 | High |
| Adisakwattana et al. (2020)^176^ | Population based | Thailand | Kato-Katz | High | Upper-middle | 567 | 12 | Low |
| Chai et al. (2020)^177^ | Children | Myanmar | Kato-Katz | Medium | Lower-middle | 2227 | 383 | Low |
| Prommi et al. (2020)^177^ | Population based | Thailand | Kato-Katz | High | Upper-middle | 1047 | 9 | Low |
| Sha-Sha et al. (2020)^178^ | Population based | China | Kato-Katz | High | Upper-middle | 202,880 | 670 | Low |
| Subahar et al. (2020)^179^ | Children | Indonesia | Kato-Katz | High | Lower-middle | 219 | 14 | High |
| Wandra et al. (2020)^180^ | Population based | Indonesia | Kato-Katz | High | Lower-middle | 368 | 66 | Low |
| Zeng et al. (2020)^181^ | Children | Myanmar | Kato-Katz | Medium | Lower-middle | 988 | 12 | Low |
| Zhu et al. (2020)^182^ | Population based | China | Kato-Katz | High | Upper-middle | 133,231 | 1032 | Low |
| Zhu et al. (2020)^183^ | Population based | China | Kato-Katz | High | Upper-middle | 305,081 | 2338 | Low |
| Labana et al. (2021)^184^ | Children | Philippines | Routine parasitological methods* | High | Lower-middle | 478 | 98 | Moderate |
| Wattanawong et al. (2021)^185^ | Population based | Thailand | Routine parasitological methods* | High | Upper-middle | 16,187 | 35 | Low |
| Wong et al. (2021)^186^ | Population based | Malaysia | Routine parasitological methods* | Very high | upper-middle | 224 | 1 | Moderate |
| Aung et al. (2021)^187^ | Children | Myanmar | Kato-Katz | Medium | Lower-middle | 363 | 41 | Moderate |
| Bria et al. (2021)^188^ | Children | Indonesia | Kato-Katz | High | Lower-middle | 130 | 50 | High |
| Djuardi et al. (2021)^189^ | Children | Indonesia | Kato-Katz | High | Lower-middle | 393 | 129 | Moderate |
| Feng et al. (2021)^190^ | Population based | China | Kato-Katz | High | Upper-middle | 23,522 | 13 | Low |
| Jin et al. (2021)^191^ | Population based | Laos PDR | Kato-Katz | Medium | Lower-middle | 198 | 3 | High |
| Mationg et al. (2021)^192^ | Children | Philippines | Kato-Katz | High | Lower-middle | 1995 | 330 | Low |
| Mationg et al. (2021)^192^ | Children | Philippines | Kato-Katz | High | Lower-middle | 2086 | 355 | Low |
| Latin America and the Caribbean |  |  |  |  |  |  |  |  |
| Devera et al. (2010)^193^ | Children | Venezuela | Routine parasitological methods* | High | High | 274 | 43 | Moderate |
| dos Santos et al. (2010)^194^ | Population based | Brazil | Routine parasitological methods* | High | Upper-middle | 431 | 6 | Moderate |
| Melo et al. (2010)^195^ | Children | Brazil | Routine parasitological methods* | High | Upper-middle | 54 | 18 | High |
| Vidal et al. (2010)^196^ | Children | Chile | Routine parasitological methods* | Very high | High | 68,142 | 2460 | Low |
| Barreto et al. (2010)^196^ | Children | Brazil | Kato-Katz | High | Upper-middle | 681 | 166 | Low |
| Kurup et al. (2010)^197^ | Children | Saint Lucia | Kato-Katz | High | Upper-middle | 554 | 97 | Low |
| Lins Fonseca et al. (2010)^198^ | Children | Brazil | Kato-Katz | High | Upper-middle | 2523 | 633 | Low |
| Mascarini-Serra et al. (2010)^199^ | Children | Brazil | Kato-Katz | High | Upper-middle | 1619 | 468 | Low |
| Rosewell et al. (2010)^200^ | Children | Nicaragua | Kato-Katz | Medium | Lower-middle | 199 | 68 | Moderate |
| Rosewell et al. (2010)^200^ | Children | Nicaragua | Kato-Katz | Medium | Lower-middle | 211 | 9 | Moderate |
| Rosewell et al. (2010)^200^ | Children | Nicaragua | Kato-Katz | Medium | Lower-middle | 224 | 97 | Moderate |
| Rosewell et al. (2010)^200^ | Children | Nicaragua | Kato-Katz | Medium | Lower-middle | 246 | 8 | Moderate |
| Conceição Silva et al. (2011)^201^ | Children | Brazil | Routine parasitological methods* | High | Upper-middle | 220 | 118 | Moderate |
| Molina et al. (2011)^202^ | Children | Argentina | Routine parasitological methods* | Very high | Upper-middle | 244 | 7 | Moderate |
| Monárrez-Espino et al. (2011)^203^ | Children | Mexico | Routine parasitological methods* | High | Upper-middle | 194 | 50 | High |
| Vasconcelos et al. (2011)^204^ | Children | Brazil | Kato-Katz | High | Upper-middle | 383 | 84 | Moderate |
| Valverde et al. (2011)^205^ | Population based | Brazil | Other methods¶ | High | Upper-middle | 463 | 120 | Moderate |
| Canete et al. (2012)^206^ | Children | Cuba | Routine parasitological methods* | High | Upper-middle | 104 | 7 | High |
| Cardona et al. (2012)^207^ | Population based | Colombia | Routine parasitological methods* | High | Upper-middle | 309 | 33 | Moderate |
| Gamboa et al. (2012)^208^ | Children | Argentina | Routine parasitological methods* | Very high | Upper-middle | 878 | 17 | Moderate |
| Gamboa et al. (2012)^208^ | Children | Argentina | Routine parasitological methods* | Very high | Upper-middle | 1015 | 26 | Low |
| Lander et al. (2012)^209^ | Children | Brazil | Routine parasitological methods* | High | Upper-middle | 376 | 34 | Moderate |
| Zulbey et al. (2012)^210^ | Adults | Venezuela | Routine parasitological methods* | High | High | 95 | 6 | High |
| Alcantara-Neves et al. (2012)^211^ | Children | Brazil | Kato-Katz | High | Upper-middle | 1182 | 190 | Low |
| Branco et al. (2012)^212^ | Population based | Brazil | Kato-Katz | High | Upper-middle | 185 | 27 | High |
| Carvalho et al. (2012)^213^ | Population based | Brazil | Kato-Katz | High | Upper-middle | 270 | 13 | High |
| Machicado et al. (2012)^214^ | Children | Peru | Kato-Katz | High | Upper-middle | 73 | 4 | High |
| Quintero et al. (2012)^215^ | Population based | Venezuela | Kato-Katz | High | High | 3388 | 126 | Low |
| Damazio et al. (2013)^216^ | Population based | Brazil | Routine parasitological methods* | High | Upper-middle | 82 | 1 | High |
| Gutierrez-Jimenez et al. (2013)^217^ | Children | Mexico | Routine parasitological methods* | High | Upper-middle | 250 | 84 | High |
| Nobre et al. (2013)^218^ | Children | Brazil | Routine parasitological methods* | High | Upper-middle | 214 | 20 | Moderate |
| Verhagen et al. (2013)^219^ | Children | Venezuela | Routine parasitological methods* | High | High | 390 | 104 | Moderate |
| Gutierrez et al. (2014)^220^ | Children | Panama | Routine parasitological methods* | Very high | Upper-middle | 100 | 14 | High |
| Munoz-Antoli et al. (2014)^221^ | Children | Nicaragua | Routine parasitological methods* | Medium | Lower-middle | 382 | 80 | Moderate |
| Alcantara-Neves et al. (2014)^222^ | Children | Brazil | Kato-Katz | High | Upper-middle | 1445 | 228 | Low |
| Bragagnoli et al. (2014)^223^ | Children | Brazil | Kato-Katz | High | Upper-middle | 1124 | 260 | Low |
| Capon-Robins et al. (2014)^224^ | Population based | Ecuador | Kato-Katz | High | Upper-middle | 211 | 102 | Moderate |
| Gabrie et al. (2014)^225^ | Children | Honduras | Kato-Katz | Medium | Lower-middle | 320 | 97 | Moderate |
| Torre et al. (2014)^226^ | Children | Honduras | Kato-Katz | Medium | Lower-middle | 2554 | 569 | Low |
| Gamboa et al. (2014)^227^ | Population based | Argentina | Other methods¶ | Very high | Upper-middle | 653 | 7 | Moderate |
| Silva et al. et al. (2014)^228^ | Children | Brazil | Other methods¶ | High | Upper-middle | 1195 | 314 | Low |
| Cimino et al. (2015)^229^ | Population based | Argentina | Routine parasitological methods* | Very high | Upper-middle | 99 | 53 | High |
| de Oliveira Serra et al. (2015)^230^ | Children | Brazil | Routine parasitological methods* | High | Upper-middle | 370 | 49 | Moderate |
| Dib et al. (2015)^231^ | Children | Argentina | Routine parasitological methods* | Very high | Upper-middle | 115 | 45 | High |
| Fernandez-Nino et al. (2015)^232^ | Children | Colombia | Kato-Katz | High | Upper-middle | 154 | 10 | High |
| Periago et al. (2015)^233^ | Children | Brazil | Kato-Katz | High | Upper-middle | 1260 | 252 | Low |
| Rollemberg et al. (2015)^234^ | Population based | Brazil | Kato-Katz | High | Upper-middle | 500 | 246 | Low |
| Echazu et al. (2015)^235^ | Population based | Argentina | Other methods¶ | Very high | Upper-middle | 339 | 63 | Moderate |
| Cabada et al. (2015)^236^ | Children | Peru | Other methods¶ | High | Upper-middle | 240 | 34 | Moderate |
| Barra et al. (2016)^237^ | Children | Chile | Routine parasitological methods* | Very high | High | 103 | 1 | High |
| Bouwmans et al. (2016)^238^ | Children | Colombia | Routine parasitological methods* | High | Upper-middle | 239 | 3 | Moderate |
| Casavechia et al. (2016)^239^ | Population based | Brazil | Routine parasitological methods* | High | Upper-middle | 775 | 0 | Low |
| Dib et al. (2016)^231^ | Children | Argentina | Routine parasitological methods* | Very high | Upper-middle | 90 | 35 | High |
| Gonçalves et al. (2016)^240^ | Population based | Brazil | Routine parasitological methods* | High | Upper-middle | 594 | 317 | Low |
| Macchioni et al. (2016)^241^ | Population based | Bolivia | Routine parasitological methods* | High | Lower-middle | 223 | 3 | Moderate |
| Macchioni et al. (2016)^242^ | Children | Bolivia | Routine parasitological methods* | High | Lower-middle | 268 | 4 | Moderate |
| Da Silva et al. (2016)^243^ | Population based | Brazil | Kato-Katz | High | Upper-middle | 597 | 292 | Low |
| Lopez et al. (2016)^244^ | Children | Peru | Kato-Katz | High | Upper-middle | 1083 | 67 | Low |
| Cabada et al. (2016)^245^ | Children | Peru | Other methods¶ | High | Upper-middle | 1230 | 75 | Low |
| Choi et al. (2017)^246^ | Children | Peru | Routine parasitological methods* | High | Upper-middle | 185 | 69 | High |
| da Fonseca et al. (2017)^247^ | Children | Brazil | Routine parasitological methods* | High | Upper-middle | 233 | 41 | High |
| Incani et al. (2017)^248^ | Population based | Venezuela | Routine parasitological methods* | High | High | 148 | 73 | High |
| Muñoz-Antoli et al. (2017)^249^ | Children | Nicaragua | Routine parasitological methods* | Medium | Lower-middle | 425 | 115 | Low |
| Perez Faria et al. (2017)^250^ | Population based | Brazil | Routine parasitological methods* | High | Upper-middle | 3245 | 12 | Low |
| Segui et al. (2017)^251^ | Children | Brazil | Routine parasitological methods* | High | Upper-middle | 217 | 14 | Moderate |
| Canete et al. (2017)^252^ | Children | Cuba | Kato-Katz | High | Upper-middle | 107 | 21 | High |
| Coronato-Nunes et al. (2017)^253^ | Population based | Brazil | Kato-Katz | High | Upper-middle | 213 | 0 | Moderate |
| Ignacio et al. (2017)^254^ | Population based | Brazil | Kato-Katz | High | Upper-middle | 595 | 11 | Low |
| Cociancic et al. (2017)^255^ | Children | Argentina | Other methods¶ | Very high | Upper-middle | 211 | 10 | High |
| Costa et al. (2018)^256^ | Adults | Brazil | Routine parasitological methods* | High | Upper-middle | 6289 | 16 | Low |
| Cruz-Cruz et al. (2018)^257^ | Children | Mexico | Routine parasitological methods* | High | Upper-middle | 42 | 27 | High |
| Cruz-Cruz et al. (2018)^257^ | Children | Mexico | Routine parasitological methods* | High | Upper-middle | 64 | 0 | High |
| Giraldo-Ospina et al. (2018)^258^ | Children | Colombia | Routine parasitological methods* | High | Upper-middle | 150 | 15 | High |
| Muñoz-Antoli et al. (2018)^259^ | Children | Nicaragua | Routine parasitological methods* | Medium | Lower-middle | 1217 | 63 | Low |
| Periago et al. (2018)^260^ | Children | Argentina | Routine parasitological methods* | Very high | Upper-middle | 470 | 0 | Moderate |
| Spinicci et al. (2018)^261^ | Children | Bolivia | Routine parasitological methods* | High | Lower-middle | 520 | 1 | Low |
| de Gier et al. (2018)^163^ | Children | Cuba | Kato-Katz | High | Upper-middle | 1379 | 72 | Low |
| Moncayo et al. (2018)^262^ | Children | Ecuador | Kato-Katz | High | upper-middle | 920 | 170 | Low |
| Muñoz-Antoli et al. (2018)^263^ | Children | Nicaragua | Kato-Katz | Medium | Lower-middle | 341 | 68 | Moderate |
| Seguí et al. (2018)^264^ | Population based | Brazil | Kato-Katz | High | Upper-middle | 766 | 38 | Low |
| Barbosa et al. (2018)^265^ | Population based | Brazil | Other methods¶ | High | Upper-middle | 294 | 2 | High |
| Anselmi et al. (2019)^266^ | Population based | Ecuador | Routine parasitological methods* | High | upper-middle | 184 | 101 | High |
| Galvan-Ramirez et al. (2019)^267^ | Population based | Mexico | Routine parasitological methods* | High | Upper-middle | 104 | 6 | High |
| Pinzón-Rondon et al. (2019)^268^ | Children | Colombia | Routine parasitological methods* | High | Upper-middle | 144 | 2 | High |
| Errea et al. (2019)^269^ | Children | Peru | Kato-Katz | High | Upper-middle | 124 | 20 | High |
| Guttierrez-Jiminez et al. (2019)^270^ | Children | Mexico | Kato-Katz | High | Upper-middle | 84 | 48 | High |
| Guttierrez-Jiminez et al. (2019)^270^ | Children | Mexico | Kato-Katz | High | Upper-middle | 94 | 0 | High |
| Hernandez et al. (2019)^271^ | Children | Colombia | Kato-Katz | High | Upper-middle | 97 | 5 | High |
| Arana et al. (2020)^272^ | Children | Mexico | Routine parasitological methods* | High | upper-middle | 38 | 6 | Moderate |
| Cocioncic et al. (2020)^273^ | Children | Argentina | Routine parasitological methods* | Very high | Upper-middle | 398 | 10 | Moderate |
| Harvey et al. (2020)^274^ | Children | Brazil | Routine parasitological methods* | High | Upper-middle | 193 | 26 | High |
| Ibiapina et al. (2020)^275^ | Population based | Brazil | Routine parasitological methods* | High | Upper-middle | 39,539 | 3127 | Low |
| Gildner et al. (2020)^276^ | Population based | Ecuador | Kato-Katz | High | upper-middle | 620 | 301 | Moderate |
| Naceanceno et al. (2020)^277^ | Children | Honduras | Kato-Katz | Medium | Lower-middle | 95 | 15 | Moderate |
| Quiroz et al. (2020)^278^ | Children | Colombia | Kato-Katz | High | Upper-middle | 6045 | 683 | Low |
| Falcone et al. (2020)^279^ | Population based | Argentina | Other methods¶ | Very high | Upper-middle | 350 | 2 | Moderate |
| Aboikoni et al. (2021)^280^ | Population based | French Guiana | Routine parasitological methods* | Very high | High | 9555 | 20 | Low |
| Cociancic et al. (2021)^281^ | Children | Argentina | Routine parasitological methods* | Very high | Upper-middle | 114 | 9 | Low |
| Cociancic et al. (2021)^281^ | Children | Argentina | Routine parasitological methods* | Very high | Upper-middle | 268 | 0 | Low |
| Cociancic et al. (2021)^281^ | Children | Argentina | Routine parasitological methods* | Very high | Upper-middle | 377 | 0 | Low |
| Cociancic et al. (2021)^281^ | Children | Argentina | Routine parasitological methods* | Very high | Upper-middle | 752 | 2 | Low |
| Cociancic et al. (2021)^281^ | Children | Argentina | Routine parasitological methods* | Very high | Upper-middle | 1015 | 26 | Low |
| Cociancic et al. (2021)^281^ | Children | Argentina | Routine parasitological methods* | Very high | Upper-middle | 1411 | 34 | Low |
| De Morais et al. (2021)^282^ | Children | Brazil | Routine parasitological methods* | High | Upper-middle | 172 | 7 | Low |
| Incani et al. (2021)^283^ | Population based | Venezuela | Routine parasitological methods* | High | High | 224 | 89 | Moderate |
| Andrade et al. (2021)^284^ | Children | Ecuador | Kato-Katz | High | Upper-middle | 297 | 92 | Moderate |
| Chura et al. (2021)^285^ | Children | Bolivia | Kato-Katz | High | Lower-middle | 275 | 40 | Moderate |
| Bryan et al. (2021)^286^ | Children | Colombia | Other methods¶ | High | Upper-middle | 50 | 25 | Moderate |
| Bryan et al. (2021)^286^ | Children | Colombia | Other methods¶ | High | Upper-middle | 72 | 14 | Moderate |
| Calegar et al. (2021)^287^ | Children | Brazil | Other methods¶ | High | Upper-middle | 349 | 61 | Moderate |
| Northern Africa and Western Asia |  |  |  |  |  |  |  |  |
| AL-Haddad et al. (2010)^288^ | Children | Yemen | Routine parasitological methods* | Low | Low | 352 | 95 | High |
| Bdir et al. (2010)^289^ | Population based | Palestine | Routine parasitological methods* | High | Lower-middle | 32,666 | 53 | Moderate |
| Al-Mohammed et al. (2010)^290^ | Children | Saudi Arabia | Routine parasitological methods* | Very high | High | 1289 | 11 | Low |
| Dash et al. (2010)^291^ | Population based | United Arab Emirates | Routine parasitological methods* | Very high | High | 10,514 | 14 | Low |
| Koksal et al. (2010)^292^ | Population based | Turkey | Routine parasitological methods* | Very high | Upper-middle | 27,664 | 8 | Low |
| Bdiro et al. (2010)^289^ | Population based | Palestine | Routine parasitological methods* | High | Lower-middle | 56,193 | 57 | Low |
| Alyousefi et al. (2011)^293^ | Population based | Yemen | Routine parasitological methods* | Low | Low | 503 | 12 | Low |
| Araj et al. (2011)^294^ | Population based | Lebanon | Routine parasitological methods* | High | upper-middle | 7477 | 14 | Low |
| Calik et al. (2011)^295^ | Children | Turkey | Routine parasitological methods* | Very high | Upper-middle | 1181 | 1 | Low |
| Ekinci et al. (2011)^296^ | Children | Turkey | Routine parasitological methods* | Very high | Upper-middle | 663 | 39 | moderate |
| El Guamri et al. (2011)^297^ | Children | Morocco | Routine parasitological methods* | Medium | Lower-middle | 300 | 38 | Moderate |
| Hussein et al. (2011)^298^ | Children | Iraq | Routine parasitological methods* | Medium | Upper-middle | 730 | 41 | High |
| Hussein et al. (2011)^299^ | Children | Palestine | Routine parasitological methods* | High | Lower-middle | 735 | 12 | Moderate |
| Ibrahium et al. (2011)^300^ | Children | Egypt | Routine parasitological methods* | High | Lower-middle | 264 | 10 | Moderate |
| AL-Kubaisy et al. (2014)^301^ | Children | Iraq | Routine parasitological methods* | Medium | Upper-middle | 2033 | 10 | Low |
| Farghly et al. (2014)^302^ | Children | Egypt | Routine parasitological methods* | High | Lower-middle | 859 | 28 | Moderate |
| Hegazy et al. (2014)^303^ | Children | Egypt | Routine parasitological methods* | High | Lower-middle | 500 | 70 | Low |
| Uysal et al. (2014)^304^ | Population based | Turkey | Routine parasitological methods* | Very high | Upper-middle | 111,889 | 409 | Low |
| Aytar et al. (2015)^305^ | Children | Turkey | Routine parasitological methods* | Very high | Upper-middle | 523 | 1 | Low |
| Doni et al. (2015)^306^ | Children | Turkey | Routine parasitological methods* | Very high | Upper-middle | 333 | 25 | Moderate |
| Al-Mekhlafi et al. (2016)^307^ | Children | Yemen | Routine parasitological methods* | Low | Low | 1218 | 101 | Low |
| Alsubaie et al. (2016)^308^ | Children | Yemen | Routine parasitological methods* | Low | Low | 258 | 37 | Moderate |
| Hassen Amer et al. (2016)^309^ | Population based | Saudi Arabia | Routine parasitological methods* | Very high | High | 130 | 2 | High |
| Jaran et al. (2016)^310^ | Children | Jordan | Routine parasitological methods* | High | Upper-middle | 21,906 | 84 | Low |
| Alwabr et al. (2016)^311^ | Children | Yemen | Kato-Katz | Low | Low | 200 | 20 | Moderate |
| Gashout et al. (2017)^312^ | Population based | Libya | Routine parasitological methods* | High | upper-middle | 18,000 | 90 | High |
| Musa et al. (2017)^313^ | Population based | Iraq | Routine parasitological methods* | Medium | Upper-middle | 130,701 | 44 | Moderate |
| Saheb et al. (2017)^314^ | Population based | Iraq | Routine parasitological methods* | Medium | Upper-middle | 56,419 | 84 | Moderate |
| Alharbi et al. (2019)^315^ | Children | Yemen | Routine parasitological methods* | Low | Low | 320 | 22 | Moderate |
| Taş Cengiz et al. (2019)^316^ | Population based | Turkey | Routine parasitological methods* | Very high | Upper-middle | 69,633 | 1720 | Low |
| Al-Saqur et al. (2020)^317^ | Population based | Iraq | Routine parasitological methods* | Medium | Upper-middle | 1,707,873 | 241 | Low |
| Erdal Polat et al. (2020)^318^ | Population based | Turkey | Routine parasitological methods* | Very high | Upper-middle | 20,948 | 2 | Low |
| Hamarsheh et al. (2020)^319^ | Population based | Palestine | Routine parasitological methods* | High | Lower-middle | 137,106 | 3465 | Moderate |
| Al-Rifai et al. (2020)^320^ | Adults | United Arab Emirates | Other methods¶ | Very high | High | 86 | 5 | Low |
| Al-Hindi et al. (2021)^321^ | Children | Palestine | Routine parasitological methods* | High | Lower-middle | 305 | 1 | High |
| Shehab et al. (2021)^322^ | Population based | Egypt | Routine parasitological methods* | High | Lower-middle | 300 | 1 | High |
| Allam et al. (2021)^323^ | Children | Egypt | Kato-Katz | High | Lower-middle | 90 | 2 | Low |
| Oceania |  |  |  |  |  |  |  |  |
| Shield et al. (2013)^324^ | Children | Papua New Guinea | Other methods¶ | Medium | Lower-middle | 627 | 95 | Low |
| Bradbury et al. (2018)^325^ | Population based | Solomon Islands | Kato-Katz | Medium | Lower-middle | 257 | 163 | Moderate |
| Bradbury et al. (2018)^325^ | Population based | Solomon Islands | Kato-Katz | Medium | Lower-middle | 326 | 148 | Moderate |
| Lee et al. (2020)^326^ | Children | Solomon Islands | Routine parasitological methods* | Medium | Lower-middle | 454 | 19 | Moderate |
| Sub-Saharan Africa |  |  |  |  |  |  |  |  |
| Ahmed et al. (2010)^327^ | Children | Sudan | Routine parasitological methods* | Low | Low | 157 | 51 | Moderate |
| Ezeagwuna et al. (2010)^328^ | Children | Nigeria | Routine parasitological methods* | Low | Lower-middle | 260 | 44 | Moderate |
| Houmsou et al. (2010)^329^ | Children | Nigeria | Routine parasitological methods* | Low | Lower-middle | 1000 | 111 | Low |
| Jombo et al. (2010)^330^ | Population based | Nigeria | Routine parasitological methods* | Low | Lower-middle | 347 | 64 | Moderate |
| Mazigo et al. (2010)^331^ | Children | Tanzania | Routine parasitological methods* | Low | Lower-middle | 400 | 0 | Moderate |
| Clements et al. (2010)^332^ | Children | Burundi | Kato-Katz | Low | Low | 28,213 | 1918 | Low |
| Foday et al. (2010)^333^ | Population based | Sierra Leone | Kato-Katz | Low | Low | 514 | 27 | High |
| Knopp et al. (2010)^334^ | Population based | Zanzibar | Kato-Katz | Low | Lower-middle | 184 | 6 | Moderate |
| Knopp et al. (2010)^334^ | Population based | Zanzibar | Kato-Katz | Low | Lower-middle | 270 | 133 | Moderate |
| Koroma et al. (2010)^335^ | Children | Sierra Leone | Kato-Katz | Low | Low | 5069 | 365 | Low |
| Midzi et al. (2010)^336^ | Children | Zimbabwe | Kato-Katz | Medium | Lower-middle | 575 | 136 | Low |
| Siwila et al. (2010)^337^ | Children | Zambia | Kato-Katz | Medium | Lower-middle | 403 | 39 | Moderate |
| Sowemimo et al. (2010)^338^ | Children | Nigeria | Kato-Katz | Low | Lower-middle | 352 | 117 | Moderate |
| Oninla et al. (2010)^339^ | Children | Nigeria | Other methods¶ | Low | Lower-middle | 749 | 206 | Low |
| Damen et al. (2011)^340^ | Children | Nigeria | Routine parasitological methods* | Low | Lower-middle | 257 | 49 | Moderate |
| Ejima et al. (2011)^341^ | Children | Nigeria | Routine parasitological methods* | Low | Lower-middle | 100 | 22 | High |
| Idowu et al. (2011)^342^ | Children | Nigeria | Routine parasitological methods* | Low | Lower-middle | 133 | 30 | High |
| Kamga et al. (2011)^343^ | Children | Cameroon | Routine parasitological methods* | Medium | Lower-middle | 370 | 78 | Moderate |
| Osazuwa et al. (2011)^344^ | Children | Nigeria | Routine parasitological methods* | Low | Lower-middle | 316 | 168 | Moderate |
| Alemu et al. (2011)^345^ | Children | Ethiopia | Kato-Katz | Low | Low | 319 | 70 | Moderate |
| Furst et al. (2011)^346^ | Children | Côte d'Ivoire | Kato-Katz | Low | Lower-middle | 167 | 2 | High |
| Green et al. (2011)^347^ | Children | Uganda | Kato-Katz | Low | Low | 865 | 27 | Low |
| Halwindi et al. (2011)^348^ | Children | Zambia | Kato-Katz | Medium | Lower-middle | 986 | 162 | Low |
| Hodges et al. (2011)^349^ | Children | Guinea | Kato-Katz | Low | Low | 420 | 34 | Moderate |
| Hodges et al. (2011)^349^ | Children | Sierra Leone | Kato-Katz | Low | Low | 1760 | 26 | Low |
| Kabatereine et al. (2011)^350^ | Children | Uganda | Kato-Katz | Low | Low | 173 | 0 | Low |
| Kabatereine et al. (2011)^350^ | Children | Uganda | Kato-Katz | Low | Low | 212 | 6 | Low |
| Kabatereine et al. (2011)^350^ | Children | Uganda | Kato-Katz | Low | Low | 218 | 30 | Low |
| Kabatereine et al. (2011)^350^ | Children | Uganda | Kato-Katz | Low | Low | 331 | 10 | Low |
| Kabatereine et al. (2011)^350^ | Children | Uganda | Kato-Katz | Low | Low | 356 | 16 | Low |
| Kabatereine et al. (2011)^350^ | Children | Uganda | Kato-Katz | Low | Low | 484 | 23 | Low |
| Kabatereine et al. (2011)^350^ | Children | Uganda | Kato-Katz | Low | Low | 667 | 21 | Low |
| Kabatereine et al. (2011)^350^ | Children | Uganda | Kato-Katz | Low | Low | 962 | 54 | Low |
| Kabatereine et al. (2011)^350^ | Children | Uganda | Kato-Katz | Low | Low | 1137 | 36 | Low |
| Midzi et al. (2011)^351^ | Children | Zimbabwe | Kato-Katz | Medium | Lower-middle | 1249 | 12 | Low |
| Odiere et al. (2011)^352^ | Children | Kenya | Kato-Katz | Medium | Lower-middle | 1308 | 64 | Low |
| Ojurongbe et al. (2011)^353^ | Children | Nigeria | Kato-Katz | Low | Lower-middle | 117 | 40 | High |
| Richardson et al. (2011)^354^ | Population based | Cameroon | Kato-Katz | Medium | Lower-middle | 94 | 31 | Moderate |
| Richardson et al. (2011)^354^ | Population based | Cameroon | Kato-Katz | Medium | Lower-middle | 275 | 42 | Moderate |
| Assob et al. (2012)^355^ | Population based | Cameroon | Routine parasitological methods* | Medium | Lower-middle | 150 | 17 | High |
| Ayalew et al. (2012)^356^ | Children | Ethiopia | Routine parasitological methods* | Low | Low | 704 | 338 | Low |
| Ignatius et al. (2012)^357^ | Children | Rwanda | Routine parasitological methods* | Low | Low | 583 | 185 | Low |
| Mbuh et al. (2012)^358^ | Population based | Cameroon | Routine parasitological methods* | Medium | Lower-middle | 356 | 70 | Moderate |
| Albonico et al. (2012)^359^ | Children | Tanzania | Kato-Katz | Low | Lower-middle | 430 | 292 | Moderate |
| Bechir et al. (2012)^360^ | Adults | Chad | Kato-Katz | Low | Low | 221 | 129 | Moderate |
| Bechir et al. (2012)^360^ | Children | Chad | Kato-Katz | Low | Low | 398 | 212 | Low |
| Dorkeno et al. (2012)^361^ | Children | Togo | Kato-Katz | Low | Low | 16,440 | 66 | Low |
| Friedman et al. (2012)^362^ | Children | Tanzania | Kato-Katz | Low | Lower-middle | 352 | 24 | Moderate |
| Hodges et al. (2012)^363^ | Children | Sierra Leone | Kato-Katz | Low | Low | 515 | 9 | Low |
| Nwaneri et al. (2012)^364^ | Children | Nigeria | Kato-Katz | Low | Lower-middle | 150 | 28 | High |
| Odiere et al. (2012)^365^ | Children | Kenya | Kato-Katz | Medium | Lower-middle | 4065 | 134 | Low |
| Tchuente´ et al. (2012)^366^ | Children | Cameroon | Kato-Katz | Medium | Lower-middle | 12,243 | 1405 | Low |
| Adoubryn et al. (2012)^367^ | Children | Côte d'Ivoire | Other methods¶ | Low | Lower-middle | 386 | 20 | Moderate |
| Edelduok et al. (2013)^368^ | Children | Nigeria | Routine parasitological methods* | Low | Lower-middle | 1296 | 64 | Low |
| Emile et al. (2013)^369^ | Population based | Rwanda | Routine parasitological methods* | Low | Low | 109 | 11 | High |
| Fentie et al. (2013)^370^ | Children | Ethiopia | Routine parasitological methods* | Low | Low | 520 | 96 | Moderate |
| Gelaw et al. (2013)^371^ | Children | Ethiopia | Routine parasitological methods* | Low | Low | 304 | 18 | Low |
| King et al. (2013)^372^ | Children | Ethiopia | Routine parasitological methods* | Low | Low | 2338 | 232 | Low |
| Obala et al. (2013)^373^ | Children | Kenya | Routine parasitological methods* | Medium | Lower-middle | 797 | 38 | Low |
| Wegayehu et al. (2013)^374^ | Population based | Ethiopia | Routine parasitological methods* | Low | Low | 858 | 67 | Low |
| Abanyie et al. (2013)^375^ | Children | Nigeria | Kato-Katz | Low | Lower-middle | 690 | 368 | Low |
| Alio et al. (2013)^376^ | Children | Chad | Kato-Katz | Low | Low | 1002 | 338 | Low |
| Amare et al. (2013)^377^ | Children | Ethiopia | Kato-Katz | Low | Low | 405 | 31 | Low |
| Amollo et al. (2013)^378^ | Children | Kenya | Kato-Katz | Medium | Lower-middle | 474 | 30 | Moderate |
| Bustinduy et al. (2013)^379^ | Children | Kenya | Kato-Katz | Medium | Lower-middle | 2030 | 10 | Low |
| Furst et al. (2013)^380^ | Adults | Côte d'Ivoire | Kato-Katz | Low | Lower-middle | 195 | 0 | High |
| Katungi et al. (2013)^381^ | Children | Uganda | Kato-Katz | Low | Low | 582 | 6 | Low |
| Mahmud et al. (2013)^382^ | Children | Ethiopia | Kato-Katz | Low | Low | 583 | 28 | Low |
| Mwandawiro et al. (2013)^383^ | Children | Kenya | Kato-Katz | Medium | Lower-middle | 21,528 | 3875 | Low |
| Schmidlin et al. (2013)^384^ | Population based | Côte d'Ivoire | Kato-Katz | Low | Lower-middle | 1992 | 15 | Low |
| Tchuenté et al. (2013)^385^ | Population based | Cameroon | Kato-Katz | Medium | Lower-middle | 4130 | 805 | Low |
| Tekeste et al. (2013)^386^ | Children | Ethiopia | Kato-Katz | Low | Low | 326 | 54 | Moderate |
| Abera et al. (2014)^387^ | Children | Ethiopia | Routine parasitological methods* | Low | Low | 385 | 153 | Low |
| Akanni et al. (2014)^388^ | Children | Nigeria | Routine parasitological methods* | Low | Lower-middle | 292 | 146 | Moderate |
| Andereck et al. (2014)^389^ | Adults | Kenya | Routine parasitological methods* | Medium | Lower-middle | 344 | 21 | Moderate |
| Jejaw et al. (2014)^390^ | Population based | Ethiopia | Routine parasitological methods* | Low | Low | 434 | 120 | Moderate |
| Lobo et al. (2014)^391^ | Children | Sao Tome and Principe | Routine parasitological methods* | Medium | Lower-middle | 348 | 59 | Moderate |
| Tulu et al. (2014)^392^ | Children | Ethiopia | Routine parasitological methods* | Low | Low | 340 | 16 | Moderate |
| Yihenew et al. (2014)^393^ | Population based | Ethiopia | Routine parasitological methods* | Low | Low | 392 | 22 | Moderate |
| Bird et al. (2014)^394^ | Children | Zanzibar | Kato-Katz | Low | Lower-middle | 915 | 302 | Low |
| G/hiwot et al. (2014)^395^ | Children | Ethiopia | Kato-Katz | Low | Low | 374 | 17 | Moderate |
| Hurliman et al. (2014)^396^ | Children | Côte d'Ivoire | Kato-Katz | Low | Lower-middle | 4848 | 89 | Low |
| Ibikounlé et al. (2014)^397^ | Children | Benin | Kato-Katz | Low | Lower-middle | 1344 | 60 | Low |
| Matangila et al. (2014)^398^ | Children | DR Congo | Kato-Katz | Medium | Lower-middle | 650 | 95 | Low |
| Mathewos et al. (2014)^399^ | Children | Ethiopia | Kato-Katz | Low | Low | 261 | 104 | Moderate |
| Midzi et al. (2014)^400^ | Children | Zimbabwe | Kato-Katz | Medium | Lower-middle | 12,252 | 306 | Low |
| Mwakitalu et al. (2014)^401^ | Children | Tanzania | Kato-Katz | Low | Lower-middle | 2226 | 14 | Low |
| Sabiti et al. (2014)^402^ | Children | DR Congo | Kato-Katz | Medium | Lower-middle | 438 | 328 | Moderate |
| Schule et al. (2014)^403^ | Population based | Tanzania | Kato-Katz | Low | Lower-middle | 6366 | 433 | Low |
| Abah et al. (2015)^404^ | Children | Nigeria | Routine parasitological methods* | Low | Lower-middle | 3826 | 1989 | Low |
| Ajayi et al. (2015)^405^ | Children | Nigeria | Routine parasitological methods* | Low | Lower-middle | 167 | 60 | High |
| Bugssa et al. (2015)^406^ | Children | Ethiopia | Routine parasitological methods* | Low | Low | 523 | 220 | Moderate |
| Chioma et al. (2015)^407^ | Children | Nigeria | Routine parasitological methods* | Low | Lower-middle | 416 | 234 | Moderate |
| Dada et al. (2015)^408^ | Population based | Nigeria | Routine parasitological methods* | Low | Lower-middle | 200 | 13 | Moderate |
| Dankwa et al. (2015)^409^ | Children | Ghana | Routine parasitological methods* | Medium | Lower-middle | 230 | 7 | Moderate |
| Efunshile et al. (2015)^410^ | Children | Nigeria | Routine parasitological methods* | Low | Lower-middle | 1442 | 347 | Low |
| Gebreslassie et al. (2015)^411^ | Children | Ethiopia | Routine parasitological methods* | Low | Low | 404 | 38 | Moderate |
| Gwetu et al. (2015)^412^ | Children | South-Africa | Routine parasitological methods* | High | Upper-middle | 178 | 10 | High |
| Jejaw et al. (2015)^413^ | Children | Ethiopia | Routine parasitological methods* | Low | Low | 460 | 132 | Moderate |
| Meles et al. (2015)^414^ | Children | Ethiopia | Routine parasitological methods* | Low | Low | 402 | 131 | Moderate |
| Meurs et al. (2015)^415^ | Population based | Mozambique | Routine parasitological methods* | Low | Low | 303 | 169 | Moderate |
| Mohammed et al. (2015)^416^ | Population based | Nigeria | Routine parasitological methods* | Low | Lower-middle | 500 | 25 | Moderate |
| Mwale et al. (2015)^417^ | Children | Zambia | Routine parasitological methods* | Medium | Lower-middle | 148 | 19 | High |
| Njunda et al. (2015)^418^ | Children | Cameroon | Routine parasitological methods* | Medium | Lower-middle | 411 | 36 | Low |
| Nxasana et al. (2013)^419^ | Children | South-Africa | Routine parasitological methods* | High | Upper-middle | 162 | 47 | High |
| Odugbemi et al. (2015)^420^ | Children | Nigeria | Routine parasitological methods* | Low | Lower-middle | 120 | 11 | High |
| Oliveira et al. (2015)^421^ | Children | Angola | Routine parasitological methods* | Medium | Lower-middle | 328 | 72 | Moderate |
| Ugochi et al. (2015)^422^ | Children | Nigeria | Routine parasitological methods* | Low | Lower-middle | 337 | 68 | Moderate |
| Yahaya et al. (2015)^423^ | Population based | Nigeria | Routine parasitological methods* | Low | Lower-middle | 383 | 72 | Moderate |
| Alelign et al. (2015)^424^ | Children | Ethiopia | Kato-Katz | Low | Low | 384 | 54 | High |
| Alemayehu et al. (2015)^425^ | Children | Ethiopia | Kato-Katz | Low | Low | 384 | 37 | Moderate |
| Drabo et al. (2015)^426^ | Children | Burkina Faso | Kato-Katz | Low | Low | 3514 | 4 | Moderate |
| Emana et al. (2015) ^427^ | Children | Ethiopia | Kato-Katz | Low | Low | 302 | 60 | High |
| Freeman et al. (2015)^428^ | Children | Kenya | Kato-Katz | Medium | Lower-middle | 723 | 232 | Low |
| Freeman et al. (2015)^428^ | Children | Kenya | Kato-Katz | Medium | Lower-middle | 1305 | 14 | Low |
| Freeman et al. (2015)^428^ | Children | Kenya | Kato-Katz | Medium | Lower-middle | 1377 | 253 | Low |
| Freeman et al. (2015)^428^ | Children | Kenya | Kato-Katz | Medium | Lower-middle | 1526 | 381 | Low |
| Gashaw et al. (2015)^429^ | Children | Ethiopia | Kato-Katz | Low | Low | 550 | 194 | Moderate |
| Kepha et al. (2015)^430^ | Children | Kenya | Kato-Katz | Medium | Lower-middle | 5471 | 837 | Low |
| Kuete et al. (2015)^431^ | Population based | Cameroon | Kato-Katz | Medium | Lower-middle | 428 | 9 | Moderate |
| Kure et al. (2015)^432^ | Children | Ethiopia | Kato-Katz | Low | Low | 360 | 65 | Moderate |
| Nikolay et al. (2015)^433^ | Children | Kenya | Kato-Katz | Medium | Lower-middle | 16,488 | 3820 | Low |
| Nwalorzie et al. (2015)^434^ | Children | Nigeria | Kato-Katz | Low | Lower-middle | 220 | 90 | High |
| Salawu et al. (2015)^435^ | Children | Nigeria | Kato-Katz | Low | Lower-middle | 395 | 177 | Moderate |
| Salim et al. (2015)^436^ | Children | Tanzania | Kato-Katz | Low | Lower-middle | 992 | 0 | Low |
| Seid et al. (2015)^437^ | Children | Ethiopia | Kato-Katz | Low | Low | 442 | 22 | Moderate |
| Siwila et al. (2015)^438^ | children | Zambia | Kato-Katz | Medium | Lower-middle | 311 | 39 | Moderate |
| Siza et al. (2015)^439^ | Population based | Tanzania | Kato-Katz | Low | Lower-middle | 303 | 0 | Low |
| Siza et al. (2015)^439^ | Population based | Tanzania | Kato-Katz | Low | Lower-middle | 344 | 0 | Low |
| Siza et al. (2015)^439^ | Population based | Tanzania | Kato-Katz | Low | Lower-middle | 471 | 0 | Low |
| Siza et al. (2015)^439^ | Population based | Tanzania | Kato-Katz | Low | Lower-middle | 488 | 133 | Low |
| Siza et al. (2015)^440^ | Children | Tanzania | Kato-Katz | Low | Lower-middle | 1012 | 176 | Low |
| Siza et al. (2015)^440^ | Children | Tanzania | Kato-Katz | Low | Lower-middle | 1203 | 12 | Low |
| Siza et al. (2015)^440^ | Children | Tanzania | Kato-Katz | Low | Lower-middle | 1465 | 0 | Low |
| Siza et al. (2015)^440^ | Children | Tanzania | Kato-Katz | Low | Lower-middle | 2146 | 0 | Low |
| Shumbej et al. (2015)^441^ | Children | Ethiopia | Other methods¶ | Low | Low | 377 | 56 | Moderate |
| Frickmann et al. (2015)^442^ | Children | Madagascar | Other methods¶ | Low | Low | 410 | 72 | Moderate |
| Odinaka et al. (2015)^443^ | Children | Nigeria | Other methods¶ | Low | Lower-middle | 284 | 5 | Moderate |
| Adelakun et al. (2016)^444^ | Children | Nigeria | Routine parasitological methods* | Low | Lower-middle | 128 | 14 | High |
| Amor et al. (2016)^445^ | Children | Ethiopia | Routine parasitological methods* | Low | Low | 396 | 34 | Moderate |
| Aniwada et al. (2016)^446^ | Children | Nigeria | Routine parasitological methods* | Low | Lower-middle | 859 | 111 | Low |
| Bayoumi et al. (2016)^447^ | Children | South Sudan | Routine parasitological methods* | Low | Low | 450 | 3 | High |
| Bitew et al. (2016)^448^ | Population based | Ethiopia | Routine parasitological methods* | Low | Low | 384 | 113 | Moderate |
| Dada et al. (2016)^449^ | Population based | Nigeria | Routine parasitological methods* | Low | Lower-middle | 350 | 88 | Moderate |
| M’bondoukwé et al. (2016)^450^ | Population based | Gabon | Routine parasitological methods* | High | Upper-middle | 101 | 2 | High |
| Mekonnen et al. (2016)^451^ | Population based | Ethiopia | Routine parasitological methods* | Low | Low | 1021 | 105 | Low |
| Tyoalumun et al. (2016)^452^ | Children | Nigeria | Routine parasitological methods* | Low | Lower-middle | 418 | 0 | Moderate |
| Alemu et al. (2016)^453^ | Children | Ethiopia | Kato-Katz | Low | Low | 401 | 77 | Moderate |
| Assare et al. (2016)^454^ | Children | Côte d'Ivoire | Kato-Katz | Low | Lower-middle | 7011 | 66 | Low |
| Boko et al. (2016)^455^ | Children | Benin | Kato-Katz | Low | Lower-middle | 250 | 1 | Low |
| Boko et al. (2016)^455^ | Children | Benin | Kato-Katz | Low | Lower-middle | 250 | 4 | Low |
| Boko et al. (2016)^455^ | Children | Benin | Kato-Katz | Low | Lower-middle | 250 | 6 | Low |
| Boko et al. (2016)^455^ | Children | Benin | Kato-Katz | Low | Lower-middle | 250 | 6 | Low |
| Boko et al. (2016)^455^ | Children | Benin | Kato-Katz | Low | Lower-middle | 250 | 7 | Low |
| Boko et al. (2016)^455^ | Children | Benin | Kato-Katz | Low | Lower-middle | 250 | 8 | Low |
| Boko et al. (2016)^455^ | Children | Benin | Kato-Katz | Low | Lower-middle | 250 | 8 | Low |
| Boko et al. (2016)^455^ | Children | Benin | Kato-Katz | Low | Lower-middle | 250 | 9 | Low |
| Boko et al. (2016)^455^ | Children | Benin | Kato-Katz | Low | Lower-middle | 250 | 11 | Low |
| Boko et al. (2016)^455^ | Children | Benin | Kato-Katz | Low | Lower-middle | 250 | 12 | Low |
| Boko et al. (2016)^455^ | Children | Benin | Kato-Katz | Low | Lower-middle | 250 | 13 | Low |
| Boko et al. (2016)^455^ | Children | Benin | Kato-Katz | Low | Lower-middle | 250 | 13 | Low |
| Boko et al. (2016)^455^ | Children | Benin | Kato-Katz | Low | Lower-middle | 250 | 14 | Low |
| Boko et al. (2016)^455^ | Children | Benin | Kato-Katz | Low | Lower-middle | 250 | 14 | Low |
| Boko et al. (2016)^455^ | Children | Benin | Kato-Katz | Low | Lower-middle | 250 | 15 | Low |
| Boko et al. (2016)^455^ | Children | Benin | Kato-Katz | Low | Lower-middle | 250 | 15 | Low |
| Boko et al. (2016)^455^ | Children | Benin | Kato-Katz | Low | Lower-middle | 250 | 16 | Low |
| Boko et al. (2016)^455^ | Children | Benin | Kato-Katz | Low | Lower-middle | 250 | 17 | Low |
| Boko et al. (2016)^455^ | Children | Benin | Kato-Katz | Low | Lower-middle | 250 | 17 | Low |
| Boko et al. (2016)^455^ | Children | Benin | Kato-Katz | Low | Lower-middle | 250 | 17 | Low |
| Boko et al. (2016)^455^ | Children | Benin | Kato-Katz | Low | Lower-middle | 250 | 21 | Low |
| Boko et al. (2016)^455^ | Children | Benin | Kato-Katz | Low | Lower-middle | 250 | 21 | Low |
| Boko et al. (2016)^455^ | Children | Benin | Kato-Katz | Low | Lower-middle | 250 | 25 | Low |
| Boko et al. (2016)^455^ | Children | Benin | Kato-Katz | Low | Lower-middle | 250 | 32 | Low |
| Boko et al. (2016)^455^ | Children | Benin | Kato-Katz | Low | Lower-middle | 250 | 32 | Low |
| Boko et al. (2016)^455^ | Children | Benin | Kato-Katz | Low | Lower-middle | 250 | 33 | Low |
| Boko et al. (2016)^455^ | Children | Benin | Kato-Katz | Low | Lower-middle | 250 | 39 | Low |
| Boko et al. (2016)^455^ | Children | Benin | Kato-Katz | Low | Lower-middle | 250 | 41 | Low |
| Boko et al. (2016)^455^ | Children | Benin | Kato-Katz | Low | Lower-middle | 250 | 56 | Low |
| Boko et al. (2016)^455^ | Children | Benin | Kato-Katz | Low | Lower-middle | 250 | 58 | Low |
| Bopda et al. (2016)^456^ | Adults | Cameroon | Kato-Katz | Medium | Lower-middle | 334 | 60 | Moderate |
| Easton et al. (2016)^457^ | Population based | Kenya | Kato-Katz | Medium | Lower-middle | 796 | 103 | Low |
| Erismann et al. (2016)^458^ | Children | Burkino Faso | Kato-Katz | Low | Low | 385 | 0 | Moderate |
| Ferreira et al. (2016)^459^ | Children | Sao Tome and Principe | Kato-Katz | Medium | Lower-middle | 444 | 250 | Moderate |
| Fuhrimann et al. (2016)^460^ | Adults | Uganda | Kato-Katz | Low | Low | 915 | 53 | Low |
| Garn et al. (2016)^461^ | Children | Kenya | Kato-Katz | Medium | Lower-middle | 4404 | 749 | Low |
| Grimes et al. (2016)^462^ | Children | Ethiopia | Kato-Katz | Low | Low | 115,052 | 15302 | Low |
| Knoblauch et al. (2016)^463^ | Children | DR Congo | Kato-Katz | Medium | Lower-middle | 400 | 0 | High |
| Mirante et al. (2016)^464^ | Children | Angola | Kato-Katz | Medium | Lower-middle | 610 | 72 | Low |
| Müller et al. (2016)^465^ | Children | South-Africa | Kato-Katz | High | Upper-middle | 934 | 248 | Low |
| Ortu et al. (2016)^466^ | Children | Burundi | Kato-Katz | Low | Low | 650 | 117 | Low |
| Ortu et al. (2016)^466^ | Children | Burundi | Kato-Katz | Low | Low | 4680 | 749 | Low |
| Ortu et al. (2016)^466^ | Children | Burundi | Kato-Katz | Low | Low | 5062 | 861 | Low |
| Rasoamanamihaja et al. (2016)^467^ | Children | Madagascar | Kato-Katz | Low | Low | 1958 | 86 | Low |
| Webb et al. (2016)^468^ | Population based | Uganda | Kato-Katz | Low | Low | 1996 | 27 | low |
| Worrell et al. (2016)^469^ | Children | Kenya | Kato-Katz | Medium | Lower-middle | 676 | 157 | Low |
| Yapi et al. (2016)^470^ | Children | Côte d'Ivoire | Kato-Katz | Low | Lower-middle | 5246 | 97 | Low |
| Abdi et al. (2017)^471^ | Children | Ethiopia | Routine parasitological methods* | Low | Low | 408 | 52 | Low |
| Adeniran et al. (2017)^472^ | Children | Nigeria | Routine parasitological methods* | Low | Lower-middle | 167 | 81 | High |
| Aiemjoy et al. (2017)^473^ | Children | Ethiopia | Routine parasitological methods* | Low | Low | 212 | 23 | High |
| de Alegria et al. (2017)^474^ | Children | Angola | Routine parasitological methods* | Medium | Lower-middle | 230 | 3 | Moderate |
| Haile et al. (2017)^475^ | Children | Ethiopia | Routine parasitological methods* | Low | Low | 463 | 37 | Moderate |
| Manir et al. (2017)^476^ | Children | Nigeria | Routine parasitological methods* | Low | Lower-middle | 252 | 54 | Moderate |
| Mirisho et al. (2017)^477^ | Children | Ghana | Routine parasitological methods* | Medium | Lower-middle | 225 | 16 | Moderate |
| Onyido et al. (2017)^478^ | Children | Nigeria | Routine parasitological methods* | Low | Lower-middle | 167 | 11 | High |
| Oswald et al. (2017)^479^ | Children | Ethiopia | Routine parasitological methods* | Low | Low | 11,099 | 1554 | Low |
| Sharif Siddig et al. (2017)^480^ | children | Sudan | Routine parasitological methods* | Low | Low | 120 | 1 | High |
| Shitta et al. (2017)^481^ | Population based | Nigeria | Routine parasitological methods* | Low | Lower-middle | 229 | 68 | High |
| Tadege et al. (2017)^482^ | Children | Ethiopia | Routine parasitological methods* | Low | Low | 374 | 166 | Moderate |
| Adriko et al. (2017)^483^ | Children | Uganda | Kato-Katz | Low | Low | 4285 | 22 | Low |
| Akinwande et al. (2017)^484^ | Children | Nigeria | Kato-Katz | Low | Lower-middle | 200 | 60 | High |
| Alemayehu et al. (2017)^485^ | Children | Ethiopia | Kato-Katz | Low | Low | 503 | 44 | Low |
| Campbell et al. (2017)^486^ | Population based | Cameroon | Kato-Katz | Medium | Lower-middle | 338 | 0 | Moderate |
| da Luz et al. (2017)^487^ | Children | DR Congo | Kato-Katz | Medium | Lower-middle | 224 | 36 | Moderate |
| da Luz et al. (2017)^487^ | Children | DR Congo | Kato-Katz | Medium | Lower-middle | 253 | 13 | Moderate |
| Grimes et al. (2017)^488^ | Children | Ethiopia | Kato-Katz | Low | Low | 3729 | 179 | Low |
| Halwindi et al. (2017)^489^ | Population based | Zambia | Kato-Katz | Medium | Lower-middle | 2829 | 216 | Low |
| Ito et al. (2017)^490^ | Children | Nigeria | Kato-Katz | Low | Lower-middle | 211 | 97 | Moderate |
| Kabore et al. (2017)^491^ | Children | DR Congo | Kato-Katz | Medium | Lower-middle | 1300 | 205 | Low |
| Moser et al. (2017)^492^ | Children | Lesotho | Kato-Katz | Low | Lower-middle | 301 | 0 | Moderate |
| Munisi et al. (2017)^493^ | Children | Tanzania | Kato-Katz | Low | Lower-middle | 513 | 7 | Low |
| Noche et al. (2017)^494^ | Children | Cameroon | Kato-Katz | Medium | Lower-middle | 253 | 11 | Moderate |
| Sakari et al. (2017)^495^ | Children | Kenya | Kato-Katz | Medium | Lower-middle | 361 | 12 | Moderate |
| Shittu et al. (2017)^496^ | Population based | Nigeria | Kato-Katz | Low | Lower-middle | 696 | 311 | Low |
| Sumbele et al. (2017)^497^ | Population based | Cameroon | Kato-Katz | Medium | Lower-middle | 450 | 54 | Moderate |
| Tefera et al. (2017)^498^ | Children | Ethiopia | Other methods¶ | Low | Low | 715 | 169 | Low |
| Diongue et al. (2017)^499^ | Population based | Senegal | Other methods¶ | Low | Lower-middle | 2578 | 32 | Low |
| Adu-Gyasi et al. (2018)^500^ | Population based | Ghana | Routine parasitological methods* | Medium | Lower-middle | 1569 | 23 | Low |
| Akwa et al. (2018)^501^ | Children | Nigeria | Routine parasitological methods* | Low | Lower-middle | 600 | 56 | Moderate |
| Alemu et al. (2018)^502^ | Children | Ethiopia | Routine parasitological methods* | Low | Low | 391 | 56 | Moderate |
| Forson et al. (2018)^503^ | Children | Ghana | Routine parasitological methods* | Medium | Lower-middle | 300 | 3 | Moderate |
| Hailegebriel et al. (2018)^504^ | Children | Ethiopia | Routine parasitological methods* | Low | Low | 382 | 52 | Moderate |
| Kiki-Barro et al. (2018)^505^ | Children | Côte d'Ivoire | Routine parasitological methods* | Low | Lower-middle | 515 | 4 | Moderate |
| M’bondoukwé et al. (2018)^506^ | Population based | Gabon | Routine parasitological methods* | High | Upper-middle | 270 | 37 | High |
| Nute et al. (2018)^507^ | Children | Ethiopia | Routine parasitological methods* | Low | Low | 15,455 | 2596 | Low |
| Ojja et al. (2018)^508^ | Children | Uganda | Routine parasitological methods* | Low | Low | 562 | 55 | Low |
| Okike-Osisiogu et al. (2018)^509^ | Children | Nigeria | Routine parasitological methods* | Low | Lower-middle | 1092 | 64 | Low |
| Olopade et al. (2018)^510^ | Children | Nigeria | Routine parasitological methods* | Low | Lower-middle | 384 | 85 | Moderate |
| Teklemariam et al. (2018)^511^ | Children | Ethiopia | Routine parasitological methods* | Low | Low | 280 | 100 | Moderate |
| Tekpa et al. (2018)^512^ | Children | Central African Republic | Routine parasitological methods* | Low | Low | 102 | 41 | High |
| Tine et al. (2018)^513^ | Children | Senegal | Routine parasitological methods* | Low | Lower-middle | 1163 | 2 | Low |
| Unachukwu et al. (2018)^514^ | Children | Nigeria | Routine parasitological methods* | Low | Lower-middle | 98 | 16 | High |
| Zemene et al. (2018)^515^ | Children | Ethiopia | Routine parasitological methods* | Low | Low | 247 | 3 | moderate |
| Babamale et al. (2018)^516^ | Population based | Nigeria | Kato-Katz | Low | Lower-middle | 471 | 297 | Moderate |
| Bronzan et al. (2018)^517^ | Children | Togo | Kato-Katz | Low | Low | 17,097 | 63 | Low |
| Coulibaly et al. (2018)^518^ | Population based | Côte d'Ivoire | Kato-Katz | Low | Lower-middle | 812 | 3 | Low |
| Coulibaly et al. (2018)^518^ | Population based | Côte d'Ivoire | Kato-Katz | Low | Lower-middle | 1046 | 1 | Low |
| Coulibaly et al. (2018)^518^ | Population based | Côte d'Ivoire | Kato-Katz | Low | Lower-middle | 2447 | 8 | Low |
| Dejon-Agobe et al. (2018)^519^ | Children | Gabon | Kato-Katz | High | Upper-middle | 739 | 127 | Moderate |
| Fischer et al. (2018)^520^ | Population based | Liberia | Kato-Katz | Low | Low | 225 | 180 | Moderate |
| Fisher et al. (2018)^520^ | Population based | Liberia | Kato-Katz | Low | Low | 353 | 34 | Moderate |
| Hürlimann et al. (2018)^521^ | Population based | Côte d'Ivoire | Kato-Katz | Low | Lower-middle | 810 | 3 | Low |
| Ibikounle et al. (2018)^522^ | Children | Benin | Kato-Katz | Low | Lower-middle | 19,250 | 1030 | Low |
| Ibrahim et al. (2018)^523^ | Children | Ethiopia | Kato-Katz | Low | Low | 340 | 56 | Low |
| Molla et al. (2018)^524^ | Children | Ethiopia | Kato-Katz | Low | Low | 443 | 96 | Moderate |
| Tadesse Leta et al. (2018)^525^ | Children | Ethiopia | Kato-Katz | Low | Low | 2650 | 164 | Low |
| Teshale et al. (2018)^526^ | Children | Ethiopia | Kato-Katz | Low | Low | 410 | 9 | Low |
| Tchakounté et al. (2018)^527^ | Population based | Cameroon | Other methods¶ | Medium | Lower-middle | 182 | 9 | High |
| Alemu et al. (2019)^528^ | Children | Ethiopia | Routine parasitological methods* | Low | Low | 351 | 31 | Moderate |
| Aribodor et al. (2019)^529^ | Children | Nigeria | Routine parasitological methods* | Low | Lower-middle | 236 | 95 | Moderate |
| Dahal et al. (2019)^530^ | Children | Nigeria | Routine parasitological methods* | Low | Lower-middle | 136 | 35 | High |
| Geus et al. (2019)^531^ | Children | Rwanda | Routine parasitological methods* | Low | Low | 878 | 308 | Low |
| Gizaw et al. (2019)^532^ | Children | Ethiopia | Routine parasitological methods* | Low | Low | 225 | 45 | Moderate |
| Orish et al. (2019)^533^ | Children | Ghana | Routine parasitological methods* | Medium | Lower-middle | 550 | 7 | Low |
| Sitotaw et al. (2019)^534^ | Children | Ethiopia | Routine parasitological methods* | Low | Low | 406 | 3 | Low |
| Tekalign et al. (2019)^535^ | Population based | Ethiopia | Routine parasitological methods* | Low | Low | 377 | 58 | Moderate |
| Tongjura et al. (2019)^536^ | Children | Nigeria | Routine parasitological methods* | Low | Lower-middle | 200 | 23 | Moderate |
| von Huth et al. (2019)^537^ | Children | Guinea-Bissau | Other methods¶ | Low | Low | 1274 | 1 | Low |
| Bah et al. (2019)^538^ | Children | Sierra Leone | Kato-Katz | Low | Low | 2777 | 183 | Low |
| Bekana et al. (2019)^539^ | Children | Ethiopia | Kato-Katz | Low | Low | 317 | 45 | Moderate |
| Gichuki et al. (2019)^540^ | Population based | Kenya | Kato-Katz | Medium | Lower-middle | 3292 | 0 | Low |
| Gyang et al. (2019)^541^ | Children | Nigeria | Kato-Katz | Low | Lower-middle | 384 | 238 | Moderate |
| Hakami et al. (2019)^542^ | Population based | Madagascar | Kato-Katz | Low | Low | 571 | 408 | Moderate |
| Halliday et al. (2019)^543^ | Population based | Kenya | Kato-Katz | Medium | Lower-middle | 19,684 | 78 | Low |
| Ihejirika et al. (2019)^544^ | Children | Nigeria | Kato-Katz | Low | Lower-middle | 300 | 12 | Moderate |
| Loukouri et al. (2019)^545^ | Population based | Côte d'Ivoire | Kato-Katz | Low | Lower-middle | 1905 | 10 | Low |
| Mekonnen et al. (2019)^546^ | Children | Ethiopia | Kato-Katz | Low | Low | 310 | 21 | Moderate |
| Nkengni et al. (2019)^547^ | Children | Cameroon | Kato-Katz | Medium | Lower-middle | 245 | 21 | Moderate |
| Osman et al. (2019)^548^ | Children | Ethiopia | Kato-Katz | Low | Low | 387 | 57 | Moderate |
| Sacolo-Gwebu et al. (2019)^549^ | Children | South-Africa | Kato-Katz | High | Upper-middle | 998 | 183 | Low |
| Takeuchi et al. (2019)^550^ | Children | Kenya | Kato-Katz | Medium | Lower-middle | 274 | 1 | Moderate |
| Tuasha et al. (2019)^551^ | Population based | Ethiopia | Kato-Katz | Low | Low | 427 | 145 | Moderate |
| Weldesenbet et al. (2019)^552^ | Children | Ethiopia | Kato-Katz | Low | Low | 600 | 18 | Low |
| Addisu et al. (2020)^553^ | Population based | Kenya | Routine parasitological methods* | Medium | Lower-middle | 8002 | 1353 | Low |
| Aramendia et al. (2020)^554^ | Population based | Ethiopia | Routine parasitological methods* | Low | Low | 792 | 20 | Low |
| Christian et al. (2020)^555^ | Population based | DR Congo | Routine parasitological methods* | Medium | Lower-middle | 2122 | 67 | High |
| Gebereselassie et al. (2020)^556^ | Children | Ethiopia | Routine parasitological methods* | Low | Low | 422 | 47 | Moderate |
| Hassan et al. (2020)^557^ | Children | Sudan | Routine parasitological methods* | Low | Low | 134 | 7 | Moderate |
| Kpene et al. (2020)^558^ | Children | Ghana | Routine parasitological methods* | Medium | Lower-middle | 150 | 2 | Moderate |
| Odoemene et al. (2020)^559^ | Children | Nigeria | Routine parasitological methods* | Low | Lower-middle | 1060 | 411 | Low |
| Osman et al. (2020)^548^ | Children | Ethiopia | Routine parasitological methods* | Low | Low | 387 | 57 | Moderate |
| Rosine Ruth et al. (2020)^560^ | Children | Cameroon | Routine parasitological methods* | Medium | Lower-middle | 493 | 30 | Low |
| Santana Ferreira et al. (2020)^561^ | Children | Mozambique | Routine parasitological methods* | Low | Low | 831 | 13 | Low |
| Sitotaw et al. (2020)^562^ | Children | Ethiopia | Routine parasitological methods* | Low | Low | 383 | 87 | Moderate |
| Tigist Tiruneh et al. (2020)^563^ | Children | Ethiopia | Routine parasitological methods* | Low | Low | 387 | 22 | Moderate |
| Yeshitila et al. (2020)^564^ | Children | Ethiopia | Routine parasitological methods* | Low | Low | 312 | 8 | Moderate |
| Yoseph et al. (2020)^565^ | Children | Ethiopia | Routine parasitological methods* | Low | Low | 622 | 67 | Low |
| Abaka-Yawson et al. (2020)^566^ | Children | Ghana | Kato-Katz | Medium | Lower-middle | 152 | 31 | High |
| Abebaw et al. (2020)^567^ | Children | Ethiopia | Kato-Katz | Low | Low | 217 | 9 | High |
| Abudho et al. (2020)^568^ | Children | Kenya | Kato-Katz | Medium | Lower-middle | 295 | 23 | High |
| Allan et al. (2020)^569^ | Children | Kenya | Kato-Katz | Medium | Lower-middle | 172 | 60 | High |
| Amare et al. (2020)^570^ | Children | Ethiopia | Kato-Katz | Low | Low | 850 | 159 | Low |
| Asfaw et al. (2020)^571^ | Children | Ethiopia | Kato-Katz | Low | Low | 2462 | 457 | Low |
| Chege et al. (2020)^572^ | Children | Kenya | Kato-Katz | Medium | Lower-middle | 248 | 1 | Moderate |
| Dejon-Agobe et al. (2020)^573^ | Children | Gabon | Kato-Katz | High | Upper-middle | 472 | 15 | Moderate |
| Gebreyesus et al. (2020)^574^ | Children | Ethiopia | Kato-Katz | Low | Low | 3162 | 1515 | Low |
| Gitore et al. (2020)^575^ | Children | Ethiopia | Kato-Katz | Low | Low | 1080 | 121 | Low |
| Kabatende et al. (2020)^576^ | Children | Rwanda | Kato-Katz | Low | Low | 4998 | 2495 | Low |
| Kiiti et al. (2020)^577^ | Children | Kenya | Kato-Katz | Medium | Lower-middle | 278 | 32 | Moderate |
| Kiiti et al. (2020)^577^ | Children | Kenya | Kato-Katz | Medium | Lower-middle | 392 | 32 | Moderate |
| Kim et al. (2020)^578^ | Children | Tanzania | Kato-Katz | Low | Lower-middle | 1279 | 461 | Low |
| Leta et al. (2020)^579^ | Children | Ethiopia | Kato-Katz | Low | Low | 153,238 | 19615 | Low |
| Midzi et al. (2020)^580^ | Children | Zimbabwe | Kato-Katz | Medium | Lower-middle | 13,195 | 343 | Low |
| Njambi et al. (2020)^581^ | Children | Kenya | Kato-Katz | Medium | Lower-middle | 180 | 1 | High |
| Ntonifor et al. (2020)^582^ | Population based | Cameroon | Kato-Katz | Medium | Lower-middle | 358 | 23 | Moderate |
| Okoyo et al. (2020)^583^ | Children | Kenya | Kato-Katz | Medium | Lower-middle | 9801 | 950 | Low |
| Okoyo et al. (2020)^583^ | Children | Kenya | Kato-Katz | Medium | Lower-middle | 21,432 | 3879 | Low |
| Palmeirim et al. (2020)^584^ | Children | Tanzania | Kato-Katz | Low | Lower-middle | 424 | 1 | Moderate |
| Pion et al. (2020)^585^ | Population based | DR Congo | Kato-Katz | Medium | Lower-middle | 413 | 58 | Moderate |
| Werunga et al. (2020)^586^ | Children | Kenya | Kato-Katz | Medium | Lower-middle | 130 | 16 | High |
| Workineh et al. (2020)^587^ | Children | Ethiopia | Kato-Katz | Low | Low | 340 | 28 | Moderate |
| Zeleke et al. (2020)^588^ | Children | Ethiopia | Kato-Katz | Low | Low | 504 | 64 | Moderate |
| Zulu et al. (2020)^589^ | Children | South-Africa | Kato-Katz | High | Upper-middle | 853 | 209 | High |
| Abera et al. (2021)^590^ | Children | Ethiopia | Routine parasitological methods* | Low | Low | 526 | 18 | Moderate |
| Alemu Belete et al. (2021)^591^ | Population based | Ethiopia | Routine parasitological methods* | Low | Low | 384 | 22 | Moderate |
| Alula et al. (2021)^592^ | Population based | Ethiopia | Routine parasitological methods* | Low | Low | 351 | 33 | Low |
| Aschale et al. (2021)^593^ | Children | Ethiopia | Routine parasitological methods* | Low | Low | 407 | 12 | Moderate |
| Cho et al. (2021)^594^ | Children | Cameroon | Routine parasitological methods* | Medium | Lower-middle | 375 | 76 | Low |
| Cho et al. (2021)^594^ | Children | Cameroon | Routine parasitological methods* | Medium | Lower-middle | 378 | 94 | Low |
| Cho et al. (2021)^594^ | Children | Cameroon | Routine parasitological methods* | Medium | Lower-middle | 422 | 62 | Low |
| Cho et al. (2021)^594^ | Children | Cameroon | Routine parasitological methods* | Medium | Lower-middle | 843 | 72 | Low |
| Damtie et al. (2021)^595^ | Children | Ethiopia | Routine parasitological methods* | Low | Low | 403 | 28 | Moderate |
| Eyayu et al. (2021)^596^ | Population based | Ethiopia | Routine parasitological methods* | Low | Low | 1240 | 26 | Low |
| Fentahun et al. (2021)^597^ | Population based | Ethiopia | Routine parasitological methods* | Low | Low | 388 | 35 | Low |
| Gasparinho et al. (2021)^598^ | Children | Angola | Routine parasitological methods* | Medium | Lower-middle | 121 | 31 | Moderate |
| Hailu et al. (2021)^599^ | Children | Ethiopia | Routine parasitological methods* | Low | Low | 645 | 146 | Low |
| Korzeniewski et al. (2021)^600^ | Population based | Central African Republic | Routine parasitological methods* | Low | Low | 950 | 640 | Low |
| Makata et al. (2021)^601^ | Children | Tanzania | Routine parasitological methods* | Low | Lower-middle | 3026 | 55 | Low |
| Shiferaw et al. (2021)^602^ | Children | Ethiopia | Routine parasitological methods* | Low | Low | 384 | 122 | Low |
| Tegen et al. (2021)^603^ | Children | Ethiopia | Routine parasitological methods* | Low | Low | 382 | 16 | Low |
| Cedric et al. (2021)^604^ | Population based | Cameroon | Other methods¶ | Medium | Lower-middle | 788 | 17 | Low |
| Andargie et al. (2021)^605^ | Population based | Ethiopia | Kato-Katz | Low | Low | 478 | 56 | Low |
| Aribodor et al. (2021)^606^ | Children | Nigeria | Kato-Katz | Low | Lower-middle | 1677 | 74 | Low |
| Ayele et al. (2021)^607^ | Children | Ethiopia | Kato-Katz | Low | Low | 390 | 35 | Moderate |
| Bosch et al. (2021)^608^ | Children | Tanzania | Kato-Katz | Low | Lower-middle | 92 | 38 | High |
| Ejigu et al. (2021)^609^ | Children | Ethiopia | Kato-Katz | Low | Low | 422 | 89 | Moderate |
| Eltantawy et al. (2021)^610^ | Children | Tanzania | Kato-Katz | Low | Lower-middle | 339 | 14 | Moderate |
| Eneanya et al. (2021)^611^ | Population based | Liberia | Kato-Katz | Low | Low | 1490 | 630 | Low |
| Goshu et al. (2021)^612^ | Population based | Ethiopia | Kato-Katz | Low | Low | 641 | 80 | Moderate |
| Zeleke et al. (2021)^613^ | Children | Ethiopia | Kato-Katz | Low | Low | 786 | 191 | Low |
| Cedric et al. (2021)^604^ | Population based | Cameroon | Other methods¶ | Medium | Lower-middle | 778 | 17 | Low |
| Habib et al. (2021)^614^ | Children | Madagascar | Other methods¶ | Low | Low | 410 | 280 | Low |
| Grau-Pujol et al. (2021)^615^ | Children | Mozambique | Other methods¶ | Low | Low | 405 | 30 | Moderate |
| Fetene et al. (2021)^616^ | Children | Ethiopia | Other methods¶ | Low | Low | 394 | 6 | Low |

**References:**

1. Ragunathan L, Kalivaradhan SK, Ramadass S, Nagaraj M, Ramesh K. Helminthic infections in school children in Puducherry, South India. *Journal of microbiology, Immunology and Infection.* 2010;43(3):228-232.

2. Rayan P, Verghese S, McDonnell PA. Geographical location and age affects the incidence of parasitic infestations in school children. *Indian Journal of Pathology and Microbiology.* 2010;53(3):498.

3. Steinmann P, Usubalieva J, Imanalieva C, et al. Rapid appraisal of human intestinal helminth infections among schoolchildren in Osh oblast, Kyrgyzstan. *Acta tropica.* 2010;116(3):178-184.

4. Khanal L, Choudhury D, Rai S, et al. Prevalence of intestinal worm infestations among school children in Kathmandu, Nepal. *Nepal Med Coll J.* 2011;13(4):272-274.

5. Marothi Y, Singh B. Prevalence of intestinal parasites at Ujjain, Madhya Pradesh, India: Five-year study. *African Journal of Microbiology Research.* 2011;5(18):2711-2714.

6. Taheri F, Namakin K, Zarban A, Sharifzadeh G. Intestinal parasitic infection among school children in South Khorasan Province, Iran. *Journal of Research in Health Sciences* 2011;11(1):45-50.

7. Gunawardena K, Kumarendran B, Ebenezer R, Gunasingha MS, Pathmeswaran A, De Silva N. Soil-transmitted helminth infections among plantation sector schoolchildren in Sri Lanka: prevalence after ten years of preventive chemotherapy. *PLoS Neglected Tropical Diseases.* 2011;5(9):e1341.

8. Matthys B, Bobieva M, Karimova G, et al. Prevalence and risk factors of helminths and intestinal protozoa infections among children from primary schools in western Tajikistan. *Parasites & vectors.* 2011;4(1):1-13.

9. Choubisa S, Jaroli V, Choubisa P, Mogra N. Intestinal parasitic infection in Bhil tribe of Rajasthan, India. *Journal of parasitic diseases.* 2012;36(2):143-148.

10. Parameshwarappa K, Chandrakanth C, Sunil B. The Prevalence of Intestinal Parasitic Infestations and the Evaluation of Different Concentration Techniques of the Stool Examination. *Journal of Clinical & Diagnostic Research.* 2012;6(7).

11. Shrestha A, Narayan K, Sharma R. Prevalence of intestinal parasitosis among school children in Baglung District of Western Nepal. *Kathmandu university medical journal.* 2012;10(1):62-65.

12. Ashok R, Suguneswari G, Satish K, Kesavaram V. Prevalence of intestinal parasitic infection in school going children in Amalapuram, Andhra Pradesh, India. *Shiraz E-Med J.* 2013;14(4):e16652.

13. Kaliappan SP, George S, Francis MR, et al. Prevalence and clustering of soil‐transmitted helminth infections in a tribal area in southern India. *Trop Med Int Health.* 2013;18(12):1452-1462.

14. Shobha M, Bithika D, Bhavesh S. The prevalence of intestinal parasitic infections in the urban slums of a city in Western India. *Journal of infection and public health.* 2013;6(2):142-149.

15. Shrestha R, Maharjan M. Prevalence of intestinal helminth parasites among school-children of Bhaktapur district, Nepal. *Nepalese Journal of Zoology.* 2013;1(1):48-58.

16. Tiwari BR, Chaudhary R, Adhikari N, Jayaswal SK, Poudel TP, Rijal KR. Prevalence of intestinal parasitic infections among school children of Dadeldhura District, Nepal. *Journal of Health and Allied Sciences.* 2013;3(1):14-16.

17. Ranjan S, Passi SJ, Singh SN. Prevalence and risk factors associated with the presence of Soil-Transmitted Helminths in children studying in Municipal Corporation of Delhi Schools of Delhi, India. *Journal of parasitic diseases.* 2015;39(3):377-384.

18. Sherkhonov T, Yap P, Mammadov S, et al. National intestinal helminth survey among schoolchildren in Tajikistan: prevalences, risk factors and perceptions. *Acta tropica.* 2013;126(2):93-98.

19. Dhanabal J, Selvadoss PP, Muthuswamy K. Comparative study of the prevalence of intestinal parasites in low socioeconomic areas from South Chennai, India. *Journal of parasitology research.* 2014;2014.

20. Abdi J, Farhadi M, Aghaee S. Prevalence of intestinal parasites among children attending the daycare centers of Ilam, western Iran. *Journal of Medical Sciences (Faisalabad).* 2014;14(3):143-146.

21. Sah RB, Pokharel PK, Paudel IS, Acharya A, Jha N, Bhattarai S. A Study of Prevalence of Intestinal Parasites and Associated Risk Factors among the School Children of Dharan, Eastern Region of Nepal. *Indian Journal of Public Health Research & Development.* 2014;5(4).

22. Jafari R, Sharifi F, Bagherpour B, Safari M. Prevalence of intestinal parasites in Isfahan city, central Iran, 2014. *Journal of Parasitic Diseases.* 2014;40(3):679-682.

23. Ullah W, Shah A, Jamal Q, Ullah S, Muhammad I, Ullah H. Prevalence of intestinal parasites among school children in District Upper Dir, Khyber Pakhtunkhwa Pakistan. *IJB.* 2014;5(1):1-8.

24. Ahmed W, Ahmad M, Shah F. Pervasiveness of intestinal protozoan and worm incursion in IDPʼs (North Waziristan agency, KPK-Pakistan) children of 6–16 years. *JPMA.* 2015;65:943-945.

25. Bhattachan B, Panta Y, Tiwari S, Sherchand J, Rai S. Intestinal parasitic infection among school children in Chitwan District Of Nepal. *Journal of Institute of Medicine.* 2015;38(2).

26. Hosseini G, Sarkari B, Moshfe A, Motazedian MH, Khabisi SA. Epidemiology of human fascioliasis and intestinal helminthes in rural areas of Boyer-Ahmad Township, Southwest Iran; A population based study. *Iranian Journal of Public Health.* 2015;44(11):1520.

27. Korzeniewski K, Augustynowicz A, Smoleń A, Lass A. Epidemiology of intestinal parasitic infections in school children in Ghazni Province, eastern Afghanistan. *Pakistan journal of medical sciences.* 2015;31(6):1421.

28. Pandey S, Lo AL, Shrestha RB. Intestinal parasitic infections among school children of Northern Kathmandu, Nepal. *Asian Pacific Journal of Tropical Disease.* 2015;5:S89-S92.

29. Sadeghi H, Borji H. A survey of intestinal parasites in a population in Qazvin, north of Iran. *Asian Pacific Journal of Tropical Disease.* 2015;5(3):231-233.

30. Supram HS, Koirala B, Rani A, Gokhale S, Bhatta D. Prevalence of intestinal parasitic infections in a tertiary care center at western Nepal: five years retrospective study. *J Pharm Biomed Sci.* 2015;5:154-159.

31. Uppal B, Perween N, Aggarwal P, Kumar SK. A comparative study of bacterial and parasitic intestinal infections in India. *Journal of Clinical and Diagnostic Research: JCDR.* 2015;9(3):DC01.

32. Benjamin-Chung J, Nazneen A, Halder AK, et al. The interaction of deworming, improved sanitation, and household flooring with soil-transmitted helminth infection in rural Bangladesh. *PLoS neglected tropical diseases.* 2015;9(12):e0004256.

33. Attaullah S, Khan BH. Worm infection among school children of University of Peshawar. *Journal of Postgraduate Medical Institute.* 2016;30(3).

34. Mahni MB, Rezaeian M, Eshrat Beigom K, et al. Prevalence of intestinal parasitic infections in Jiroft, Kerman Province, Iran. *Iranian journal of parasitology.* 2016;11(2):232.

35. Sah RB, Baral R, Shah MU, Jha N. A Study of Prevalence of Intestinal Helminthic Infections and Associated Risk Factors among the School Children of Biratnagar Submetropolitan, Eastern Region of Nepal. *Int J Curr Res Med Sci.* 2016;2(4):8-15.

36. Khan W, Khan J, Khan N, et al. Soil-transmitted helminth infections in school children of three districts of Malakand region, Khyber Pakhtunkhwa, Pakistan. *Pakistan Journal of Pharmaceutical Sciences.* 2019;32(2):799-803.

37. Korzeniewski K, Smoleń A, Augustynowicz A, Lass A. Diagnostics of intestinal parasites in light microscopy among the population of children in eastern Afghanistan. *Annals of Agricultural and Environmental Medicine.* 2016;23(4):666-670.

38. Wani SA, Amin A. Intestinal helminth infections among children of district Shopian of Kashmir Valley, India. *Journal of Parasitic Diseases.* 2016;40(4):1422-1425.

39. Dahal M, Dahal RH, Chaudhary DK. Prevalence of Cyclospora cayetanensis and other enteropathogen among children under the age of 15 years in Biratnagar, Nepal. *Asian Pac J Trop Dis.* 2017;7:75-79.

40. Korzeniewski K, Chin Chung W, Augustynowicz A, Lass A, Jong IK K. Current status of intestinal parasitic infections among inhabitants of the Ghazni and Parwan Provinces, Afghanistan. *Family Medicine & Primary Care Review.* 2017(1):23-28.

41. Mubarak MY, Wagner AL, Carlson BF, Boulton ML. Hygienic behaviors and risks for ascariasis among college students in Kabul, Afghanistan. *The American journal of tropical medicine and hygiene.* 2017;97(2):563.

42. Praharaj I, Sarkar R, Ajjampur SSR, Roy S, Kang G. Temporal trends of intestinal parasites in patients attending a tertiary care hospital in south India: A seven-year retrospective analysis. *The Indian Journal of Medical Research.* 2017;146(1):111.

43. Saki J, Khademvatan S, Foroutan-Rad M, Gharibzadeh M. Prevalence of intestinal parasitic infections in Haftkel County, southwest of Iran. *Int J Infect.* 2017;4(4):e15593.

44. Turki H, Hamedi Y, Heidari-Hengami M, Najafi-Asl M, Rafati S, Sharifi-Sarasiabi K. Prevalence of intestinal parasitic infection among primary school children in southern Iran. *Journal of parasitic Diseases.* 2017;41(3):659-665.

45. Yadav SN, Mahato S. Study on intestinal helminth parasites in school children of Rangeli Municipality of Morang District in Eastern Nepal. *American Journal of Health Research.* 2017;5(2):50-53.

46. Galgamuwa L, Iddawela D, Dharmaratne SD. Factors associated with the prevalence of Ascaris lumbricoides infection among preschool children in a plantation community, Kandy District, Sri Lanka. *Southeast Asian Journal of Tropical Medicine and Public Health.* 2016;47(6):1143-1152.

47. Ganguly S, Barkataki S, Karmakar S, et al. High prevalence of soil-transmitted helminth infections among primary school children, Uttar Pradesh, India, 2015. *Infectious diseases of poverty.* 2017;6(1):1-9.

48. Galgamuwa LS, Iddawela D, Dharmaratne SD. Prevalence and intensity of Ascaris lumbricoides infections in relation to undernutrition among children in a tea plantation community, Sri Lanka: a cross-sectional study. *BMC pediatrics.* 2018;18(1):1-9.

49. Mareeswaran N, Savitha A, Gopalakrishnan S. Prevalence of intestinal parasites among urban and rural population in Kancheepuram district of Tamil Nadu. *Int J Community Med Public Health.* 2018;5(6):2585-2589.

50. Norbu K, Mongar A, Dorji N, Drukpa LD. Prevalence of intestinal parasitic infection in patients attending National Referral Hospital, Thimphu, from 2013 to 2015: a retrospective study. *Bhutan Health Journal.* 2018;4(1):46-49.

51. Lepper HC, Prada JM, Davis EL, Gunawardena SA, Hollingsworth TD. Complex interactions in soil-transmitted helminth co-infections from a cross-sectional study in Sri Lanka. *Transactions of the Royal Society of Tropical Medicine and Hygiene.* 2018;112(8):397-404.

52. Arshad S, Khatoon N, Warind JA, Khan A, Waheed S, Khan W. The prevalence of human intestinal protozoal and helminthic infection in Karachi. *International Journal of Biology and Biotechnology.* 2019;16(2):319-323.

53. Barki MFK, Jabeen F, Dilawar S. Association of anemia with intestinal parasites in children age (5-12) years in district Bannu. *Medical Forum Monthly.* 2019;30:84-87.

54. Langbang D, Dhodapkar R, Parija SC, Premarajan K, Rajkumari N. Prevalence of intestinal parasites among rural and urban population in Puducherry, South India-A community-based study. *Journal of Family Medicine and Primary Care.* 2019;8(5):1607.

55. Shrestha J, Bhattachan B, Rai G, Park EY, Rai SK. Intestinal parasitic infections among public and private schoolchildren of Kathmandu, Nepal: prevalence and associated risk factors. *BMC research notes.* 2019;12(1):1-7.

56. Viliram P, Memon SS, Khawaja S. Prevalence of intestinal parasitic infections in children presenting to a tertiary care hospital in Karachi, Pakistan. *Rawal Medical Journal.* 2019;44(4):690-693.

57. Safi N, Warusavithana S, Alawi SAS, Atta H, Montresor A, Gabrielli AF. Elimination of morbidity due to soil-transmitted helminthiases among Afghan schoolchildren. *Acta tropica.* 2019;197:105035.

58. Abbaszadeh Afshar MJ, Barkhori Mehni M, Rezaeian M, et al. Prevalence and associated risk factors of human intestinal parasitic infections: a population-based study in the southeast of Kerman province, southeastern Iran. *BMC infectious diseases.* 2020;20(1):1-8.

59. Gupta R, Rayamajhee B, Sherchan SP, et al. Prevalence of intestinal parasitosis and associated risk factors among school children of Saptari district, Nepal: a cross-sectional study. *Tropical medicine and health.* 2020;48(1):1-9.

60. Khadka S, Sapkota S, Adhikari S, et al. Intestinal parasitoses among Chepang and Musahar community people of Makwanpur and Nawalparasi Districts of Nepal. *Acta Parasitologica.* 2021;66(1):146-154.

61. Mahmoudvand H, Badparva E, Khalaf AK, Niazi M, Khatami M, Nazer MR. Prevalence and associated risk factors of intestinal helminthic infections in children from Lorestan province, Western Iran. *Parasite epidemiology and control.* 2020;9:e00136.

62. Subba SH, Singh TS. Study on the prevalence of intestinal parasitic infections and the assessment of the efficacy of albendazole in soil-transmitted helminths in school-going children in East Sikkim. *Tropical Parasitology.* 2020;10(1):18.

63. Benjamin-Chung J, Pilotte N, Ercumen A, et al. Comparison of multi-parallel qPCR and double-slide Kato-Katz for detection of soil-transmitted helminth infection among children in rural Bangladesh. *PLoS neglected tropical diseases.* 2020;14(4):e0008087.

64. Davlin SL, Jones AH, Tahmina S, et al. Soil-transmitted helminthiasis in four districts in Bangladesh: household cluster surveys of prevalence and intervention status. *BMC Public Health.* 2020;20(1):1-12.

65. Dukpa T, Dorji N, Thinley S, et al. Soil-transmitted helminth infections reduction in Bhutan: a report of 29 years of deworming. *PLoS One.* 2020;15(1):e0227273.

66. Afridi MF, Farhat K, Ahmed Z, Ahmed H, Ali S, Qaisrani MN. Association between intestinal helminthic infections and anaemia status in preschool children in the district Skardu of Pakistan. *Journal of the Pakistan Medical Association.* 2021;71(10):2309-2312.

67. Irum S, Ahsan A, Ahmed H, et al. A demographic survey on the prevalence of gastrointestinal parasites based on socioeconomic determinants in Pakistan. *The Journal of Infection in Developing Countries.* 2021;15(11):1738-1743.

68. Khan W, Rahman H, Kamal M, et al. Risk factors associated with intestinal pathogenic parasites in schoolchildren. *Saudi Journal of Biological Sciences.* 2022.

69. Rahimi BA, Mahboobi BA, Wafa MH, Sahrai MS, Stanikzai MH, Taylor WR. Prevalence and associated risk factors of soil-transmitted helminth infections in Kandahar, Afghanistan. *BMC Infectious Diseases.* 2022;22(1):1-9.

70. Rahman HU, Khan W, Mehmood S, et al. Prevalence of cestodes infection among school children of urban parts of Lower Dir district, Pakistan. *Brazilian Journal of Biology.* 2021;82.

71. Shrestha BK, Tumbahangphe M, Shakya J, et al. Prevalence and Related Risk Factors of Intestinal Parasitosis among Private School-Going Pupils of Dharan Submetropolitan City, Nepal. *Journal of Parasitology Research.* 2021;2021.

72. Thapa N, Subedi JR, Chhetri B. Prevalence of intestinal parasites among Sarki ethnic group of Pala Rural Municipality, Baglung, Nepal. *Annals of Parasitology.* 2021;67(2):329-336.

73. Ulaganeethi R, Rajkumari N, Gururajan A, Gunalan A, Langbang D, Kumar G. Intestinal parasitic infections and its trends: a 5-year findings from a tertiary care centre, Puducherry, South India. *Journal of Parasitic Diseases.* 2021;45(2):400-405.

74. Ulhaq Z, Khan W, Khan M, et al. Prevalence of intestinal parasitic diseases in school children of rural areas of district Lower Dir, Pakistan. *Brazilian Journal of Biology.* 2021;82.

75. Ajjampur SS, Kaliappan SP, Halliday KE, et al. Epidemiology of soil transmitted helminths and risk analysis of hookworm infections in the community: Results from the DeWorm3 Trial in southern India. *PLoS neglected tropical diseases.* 2021;15(4):e0009338.

76. Kim HK, Cheun HI, Cheun BS, et al. Prevalence of Clonorchis sinensis Infections Along the Five Major Rivers in Republic of Korea, 2007. *Osong Public Health Res Perspect.* 2010;1(1):43-49.

77. Manabo CA, Frias MVG. The antihelminthic efficacy of pineapple fruit mebendazole on soil transmitted helminthiases: a randomized controlled trial. *PIDSP Journal.* 2010;11(1):35-43.

78. Ziegelbauer K, Steinmann P, Zhou H, et al. Self-rated quality of life and school performance in relation to helminth infections: case study from Yunnan, People's Republic of China. *Parasites & vectors.* 2010;3(1):1-11.

79. Ngui R, Ishak S, Chuen CS, Mahmud R, Lim YA. Prevalence and risk factors of intestinal parasitism in rural and remote West Malaysia. *PLoS Neglected Tropical Diseases.* 2011;5(3):e974.

80. Balen J, Raso G, Li Y-S, et al. Risk factors for helminth infections in a rural and a peri-urban setting of the Dongting Lake area, People’s Republic of China. *International journal for parasitology.* 2011;41(11):1165-1173.

81. Belizario Jr VY, Totañes FIG, de Leon WU, Lumampao YF, Ciro RNT. Soil-transmitted helminth and other intestinal parasitic infections among school children in indigenous people communities in Davao del Norte, Philippines. *Acta tropica.* 2011;120:S12-S18.

82. Kounnavong S, Vonglokham M, Houamboun K, Odermatt P, Boupha B. Soil-transmitted helminth infections and risk factors in preschool children in southern rural Lao People's Democratic Republic. *Transactions of the Royal Society of Tropical Medicine and Hygiene.* 2011;105(3):160-166.

83. Sayasone S, Mak TK, Vanmany M, et al. Helminth and intestinal protozoa infections, multiparasitism and risk factors in Champasack province, Lao People's Democratic Republic. *PLoS neglected tropical diseases.* 2011;5(4):e1037.

84. Conlan JV, Khamlome B, Vongxay K, et al. Soil-transmitted helminthiasis in Laos: a community-wide cross-sectional study of humans and dogs in a mass drug administration environment. *The American journal of tropical medicine and hygiene.* 2012;86(4):624.

85. Huat LB, Mitra AK, Jamil NIN, Dam PC, Mohamed HJJ, Muda WAMW. Prevalence and risk factors of intestinal helminth infection among rural Malay children. *Journal of Global Infectious Diseases.* 2012;4(1):10.

86. Sinniah B, Sabaridah I, Soe M, et al. Determining the prevalence of intestinal parasites in three Orang Asli (Aborigines) communities in Perak, Malaysia. *Trop Biomed.* 2012;29(2):200-206.

87. Ahmed A, Al-Mekhlafi HM, Azam MN, et al. Soil-transmitted helminthiasis: a critical but neglected factor influencing school participation of Aboriginal children in rural Malaysia. *Parasitology.* 2012;139(6):802-808.

88. Ezeamama AE, McGarvey ST, Hogan J, et al. Treatment for Schistosoma japonicum, reduction of intestinal parasite load, and cognitive test score improvements in school-aged children. *PLoS neglected tropical diseases.* 2012;6(5):e1634.

89. Jiang W-S, Zeng X-J, Li H-Z, et al. Comparison of fecal examinations and worm collection results in an investigation of Ascaris lumbricoides infection. *Zhongguo xue xi Chong Bing Fang zhi za zhi= Chinese Journal of Schistosomiasis Control.* 2012;24(5):540-543.

90. Ngui R, Lim YAL, Chong Kin L, Sek Chuen C, Jaffar S. Association between anaemia, iron deficiency anaemia, neglected parasitic infections and socioeconomic factors in rural children of West Malaysia. *PLoS neglected tropical diseases.* 2012;6(3):e1550.

91. Tian L-G, Chen J-X, Wang T-P, et al. Co-infection of HIV and intestinal parasites in rural area of China. *Parasites & vectors.* 2012;5(1):1-7.

92. Wang X, Zhang L, Luo R, et al. Soil-Transmitted Helminth Infections and Correlated Risk Factors in Preschool and School-Aged Children in Rural Southwest China. *PLoS One.* 2012;7(9):e45939.

93. Wu R-F, Xiao M. Survey of intestinal nematode infections in Yangzhong City from 2003 to 2011. *Zhongguo xue xi Chong Bing Fang zhi za zhi= Chinese Journal of Schistosomiasis Control.* 2012;24(4):496, 498-496, 498.

94. Yap P, Du Z-W, Chen R, et al. Soil-transmitted helminth infections and physical fitness in school-aged Bulang children in southwest China: results from a cross-sectional survey. *Parasites & vectors.* 2012;5(1):1-9.

95. Yong T-S, Shin E-H, Chai J-Y, et al. High prevalence of Opisthorchis viverrini infection in a riparian population in Takeo Province, Cambodia. *The Korean Journal of Parasitology.* 2012;50(2):173.

96. Ghani MKA, Gopal G. Ascariasis amongst the Orang Asli (aborigine) children at Pos Sinderut, Kuala Lipis, Pahang, Malaysia. *International Medical Journal.* 2013;20(1):64-65.

97. Prownebon J, Charupoonphol P, Saksirisampant P, Limvorapitak T, Seepongpun U, Saksirisampant W. Intestinal parasitic infections: high prevalence of Giardia intestinalis in children living in an orphanage compared with hill-tribe children as detected by microscopy and ELISA. *Asian Biomedicine.* 2013;7(6):855-863.

98. Jian C. Surveillance of intestinal nematode infections in Nanjing City from 2008 to 2012. *Chinese Journal of Schistosomiasis Control.* 2013;25(5):546.

99. Gan C-X, Wang Z-M, Zhao J-H. Investigation on intestinal nematode infections of rural people in Jiangning District, Nanjing City. *Zhongguo xue xi Chong Bing Fang zhi za zhi= Chinese Journal of Schistosomiasis Control.* 2013;25(6):674, 676-674, 676.

100. Liu H-H, Yang L-J, Chen M, Li T-R, Chen Y-Z. Investigation on the current situation of human soil-borne nematode infection in Shapingba district of Chongqing. *Zhongguo ji Sheng Chong xue yu ji Sheng Chong Bing za zhi= Chinese Journal of Parasitology & Parasitic Diseases.* 2013;31(2):108-109.

101. Pham-Duc P, Nguyen-Viet H, Hattendorf J, et al. Ascaris lumbricoides and Trichuris trichiura infections associated with wastewater and human excreta use in agriculture in Vietnam. *Parasitology international.* 2013;62(2):172-180.

102. Phongluxa K, Xayaseng V, Vonghachack Y, Akkhavong K, van Eeuwijk P, Odermatt P. Helminth infection in southern Laos: high prevalence and low awareness. *Parasites & vectors.* 2013;6(1):1-15.

103. Wu W-Y, Xu G-F, Lin C-Z, Chen S-J, Chen J-S. Investigation on current status of infections of soil-borne nematodes in Yunxiao County. *Zhongguo xue xi Chong Bing Fang zhi za zhi= Chinese Journal of Schistosomiasis Control.* 2013;25(1):110-111.

104. Al-Delaimy AK, Al-Mekhlafi HM, Nasr NA, et al. Epidemiology of intestinal polyparasitism among Orang Asli school children in rural Malaysia. *PLoS neglected tropical diseases.* 2014;8(8):e3074.

105. Al-Mekhlafi HM, Anuar TS, Al-Zabedi EM, et al. Does vitamin A supplementation protect schoolchildren from acquiring soil-transmitted helminthiasis? A randomized controlled trial. *Parasites & vectors.* 2014;7(1):1-9.

106. Anuar TS, Salleh FM, Moktar N. Soil-transmitted helminth infections and associated risk factors in three Orang Asli tribes in Peninsular Malaysia. *Scientific reports.* 2014;4(1):1-7.

107. Sinniah B, Hassan A, Sabaridah I, Soe M, Ibrahim Z, Ali O. Prevalence of intestinal parasitic infections among communities living in different habitats and its comparison with one hundred and one studies conducted over the past 42 years (1970 to 2013) in Malaysia. *Tropical biomedicine.* 2014;31(2):190-206.

108. Zun-Wei D, Jin-Yong J, Hong-Bin L, Ran C, Xue-Zhong W, Tian-You D. Investigation of prevalence of soil-transmitted nematode infections among Lahu Ethnic residents in Xiaojie Township, Jinghong City, Yunnan Province. *Chinese Journal of Schistosomiasis Control.* 2014;26(1):75.

109. Eom KS, Yong T-S, Sohn W-M, et al. Prevalence of helminthic infections among inhabitants of Lao PDR. *The Korean Journal of Parasitology.* 2014;52(1):51.

110. Khieu V, Schär F, Marti H, et al. Prevalence and risk factors of Strongyloides stercoralis in Takeo Province, Cambodia. *Parasites & vectors.* 2014;7(1):1-8.

111. Laymanivong S, Hangvanthong B, Keokhamphavanh B, et al. Current status of human hookworm infections, ascariasis, trichuriasis, schistosomiasis mekongi and other trematodiases in Lao People's Democratic Republic. *The American journal of tropical medicine and hygiene.* 2014;90(4):667.

112. Papier K, Williams GM, Luceres-Catubig R, et al. Childhood malnutrition and parasitic helminth interactions. *Clinical Infectious Diseases.* 2014;59(2):234-243.

113. Ruankham W, Bunchu N, Koychusakun P. Prevalence of helminthic infections and risk factors in villagers of Nanglae Sub-District, Chiang Rai Province, Thailand. *Journal of the Medical Association of Thailand= Chotmaihet Thangphaet.* 2014;97:S29-35.

114. Wang Y, Xu Y, Kong X, et al. Survey of intestinal parasitic infections and related knowledge and behavior of residents in Jiaodong area of Shandong Province. *Zhongguo xue xi chong bing fang zhi za zhi= Chinese journal of schistosomiasis control.* 2014;26(4):376-381.

115. Yang Y-H, Song J-C, Li X-H, Li R-X, Zhou H-J. Survey on intestinal nematode infections among school students in Tengchong County. *Zhongguo ji Sheng Chong xue yu ji Sheng Chong Bing za zhi= Chinese Journal of Parasitology & Parasitic Diseases.* 2014;32(2):163-164.

116. Schär F, Inpankaew T, Traub RJ, et al. The prevalence and diversity of intestinal parasitic infections in humans and domestic animals in a rural Cambodian village. *Parasitology international.* 2014;63(4):597-603.

117. Kaewpitoon SJ, Loyd RA, Kaewpitoon N. Home Healthcare Program for Soil-Transmitted Helminthiasis in Schoolchildren along the Mekong River Basin. *Journal of Medical Association of Thailand.* 2015;98(4):1-8.

118. Ngui R, Aziz S, Chua KH, et al. Patterns and risk factors of soil-transmitted Helminthiasis among Orang Asli subgroups in Peninsular Malaysia. *The American journal of tropical medicine and hygiene.* 2015;93(2):361.

119. Sungkar S, Pohan AP, Ramadani A, et al. Heavy burden of intestinal parasite infections in Kalena Rongo village, a rural area in South West Sumba, eastern part of Indonesia: a cross sectional study. *BMC Public Health.* 2015;15(1):1-6.

120. Zhao A, Gao H, Li B, et al. Potential contribution of iron deficiency and multiple factors to anemia among 6-to 72-month-old children in the Kokang area of Myanmar. *The American journal of tropical medicine and hygiene.* 2015;93(4):836.

121. Chai J-Y, Sohn W-M, Jung B-K, et al. Intestinal helminths recovered from humans in Xieng Khouang Province, Lao PDR with a particular note on Haplorchis pumilio infection. *The Korean Journal of Parasitology.* 2015;53(4):439.

122. Liu C, Luo R, Yi H, et al. Soil-transmitted helminths in southwestern China: a cross-sectional study of links to cognitive ability, nutrition, and school performance among children. *PLoS neglected tropical diseases.* 2015;9(6):e0003877.

123. Ross AG, Olveda RM, McManus DP, et al. Risk factors for human helminthiases in rural Philippines. *International Journal of Infectious Diseases.* 2017;54:150-155.

124. Sayasone S, Utzinger J, Akkhavong K, Odermatt P. Repeated stool sampling and use of multiple techniques enhance the sensitivity of helminth diagnosis: a cross-sectional survey in southern Lao People's Democratic Republic. *Acta tropica.* 2015;141:315-321.

125. Sayasone S, Utzinger J, Akkhavong K, Odermatt P. Multiparasitism and intensity of helminth infections in relation to symptoms and nutritional status among children: a cross-sectional study in southern Lao People's Democratic Republic. *Acta tropica.* 2015;141:322-331.

126. Soares Magalhães RJ, Salamat MS, Leonardo L, et al. Mapping the risk of soil-transmitted helminthic infections in the Philippines. *PLoS neglected tropical diseases.* 2015;9(9):e0003915.

127. Vonghachack Y, Sayasone S, Bouakhasith D, Taisayavong K, Akkavong K, Odermatt P. Epidemiology of Strongyloides stercoralis on Mekong islands in southern Laos. *Acta tropica.* 2015;141:289-294.

128. Xiao P-L, Zhou Y-B, Chen Y, et al. Prevalence and risk factors of Ascaris lumbricoides (Linnaeus, 1758), Trichuris trichiura (Linnaeus, 1771) and HBV infections in Southwestern China: a community-based cross sectional study. *Parasites & Vectors.* 2015;8(1):1-11.

129. Yong T-S, Chai J-Y, Sohn W-M, et al. Prevalence of intestinal helminths among inhabitants of Cambodia (2006-2011). *The Korean journal of parasitology.* 2014;52(6):661.

130. Gordon CA, McManus DP, Acosta LP, et al. Multiplex real-time PCR monitoring of intestinal helminths in humans reveals widespread polyparasitism in Northern Samar, the Philippines. *International journal for parasitology.* 2015;45(7):477-483.

131. Chin YT, Lim YAL, Chong CW, et al. Prevalence and risk factors of intestinal parasitism among two indigenous sub-ethnic groups in Peninsular Malaysia. *Infectious diseases of poverty.* 2016;5(1):1-15.

132. Nithyamathi K, Chandramathi S, Kumar S. Predominance of Blastocystis sp. infection among school children in Peninsular Malaysia. *PLoS One.* 2016;11(2):e0136709.

133. Rajoo Y, Ambu S, Lim YAL, et al. Neglected intestinal parasites, malnutrition and associated key factors: a population based cross-sectional study among indigenous communities in Sarawak, Malaysia. *PLoS One.* 2017;12(1):e0170174.

134. De Gier B, Nga TT, Winichagoon P, et al. Species-specific associations between soil-transmitted helminths and micronutrients in Vietnamese schoolchildren. *The American journal of tropical medicine and hygiene.* 2016;95(1):77.

135. Fang-Wei W, Li-Bo W, Ran C, et al. Investigation of soil-transmitted nematode infections in Xiding Township, Menghai County, Yunnan Province. *Zhongguo xue xi Chong Bing Fang zhi za zhi= Chinese Journal of Schistosomiasis Control.* 2016;29(1):93-95.

136. Hung BK, Van De N, Le Van Duyet J-YC. Prevalence of soil-transmitted helminths and molecular clarification of hookworm species in ethnic Ede primary schoolchildren in Dak Lak province, southern Vietnam. *The Korean Journal of Parasitology.* 2016;54(4):471.

137. Laymanivong S, Hangvanthong B, Insisiengmay B, et al. First molecular identification and report of genetic diversity of Strongyloides stercoralis, a current major soil-transmitted helminth in humans from Lao People’s Democratic Republic. *Parasitology research.* 2016;115(8):2973-2980.

138. Campbell SJ, Nery SV, D’Este CA, et al. Water, sanitation and hygiene related risk factors for soil-transmitted helminth and Giardia duodenalis infections in rural communities in Timor-Leste. *International Journal for Parasitology.* 2016;46(12):771-779.

139. Llewellyn S, Inpankaew T, Nery SV, et al. Application of a multiplex quantitative PCR to assess prevalence and intensity of intestinal parasite infections in a controlled clinical trial. *PLoS neglected tropical diseases.* 2016;10(1):e0004380.

140. Yi-Sha H, Yan-Jing L, Chao-Yong X. Analysis of human intestinal nematode infections in Nanjing City from 2006 to 2015. *Chinese Journal of Schistosomiasis Control.* 2017;29(5):637.

141. Kitvatanachai S, Taylor A, Rhongbutsri P, Pongstaporn W. Determine the prevalence of intestinal and soil-transmitted helminths using different copromicroscopic techniques in Krabi Province, Thailand. *Asian Pac J Trop Dis.* 2017;7(12):719-723.

142. Lee M-R, Shin H-E, Chung B-S, et al. Intestinal Parasite Infections among Inhabitants in Yanbian Prefecture, Jilin Province, China. *The Korean Journal of Parasitology.* 2017;55(5):579.

143. Liao C-W, Chiu K-C, Chiang I-C, et al. Prevalence and risk factors for intestinal parasitic infection in schoolchildren in Battambang, Cambodia. *The American Journal of Tropical Medicine and Hygiene.* 2017;96(3):583.

144. Nanthavong N, Black AP, Khattignavong P, et al. High prevalence of intestinal worms in children up to 5 years of age in Huaphan province, Lao People's Democratic Republic (PDR). *Parasite epidemiology and control.* 2017;2(3):114-117.

145. Punsawad C, Phasuk N, Bunratsami S, Thongtup K, Siripakonuaong N, Nongnaul S. Prevalence of intestinal parasitic infection and associated risk factors among village health volunteers in rural communities of southern Thailand. *BMC Public Health.* 2017;17(1):1-9.

146. Ribas A, Jollivet C, Morand S, et al. Intestinal parasitic infections and environmental water contamination in a rural village of northern Lao PDR. *The Korean Journal of Parasitology.* 2017;55(5):523.

147. Darlan DM, Alexandra TS, Tala ZZ. Soil transmitted helminth infections in medan: A cross-sectional study of the correlation between the infection and nutritional status among elementary school children. *Family Medicine & Primary Care Review.* 2017;19(2):98-103.

148. Darlan DM, Tala ZZ, Amanta C, Warli SM, Arrasyid NK. Correlation between soil transmitted helminth infection and eosinophil levels among primary school children in Medan. *Open access Macedonian journal of medical sciences.* 2017;5(2):142.

149. Dunn JC, Bettis AA, Wyine NY, et al. A cross-sectional survey of soil-transmitted helminthiases in two Myanmar villages receiving mass drug administration: epidemiology of infection with a focus on adults. *Parasites & vectors.* 2017;10(1):1-10.

150. Liu C, Lu L, Zhang L, et al. More poop, more precision: Improving epidemiologic surveillance of soil-transmitted helminths with multiple fecal sampling using the Kato–Katz technique. *The American journal of tropical medicine and hygiene.* 2017;97(3):870.

151. Liwanag HJ, Uy J, Bataller R, et al. Soil-transmitted helminthiasis and schistosomiasis in children of poor families in Leyte, Philippines: lessons for disease prevention and control. *Journal of tropical pediatrics.* 2017;63(5):335-345.

152. Vonghachack Y, Sayasone S, Khieu V, et al. Comparison of novel and standard diagnostic tools for the detection of Schistosoma mekongi infection in Lao People's Democratic Republic and Cambodia. *Infectious diseases of poverty.* 2017;6(04):94-106.

153. Vonghachack Y, Odermatt P, Taisayyavong K, Phounsavath S, Akkhavong K, Sayasone S. Transmission of Opisthorchis viverrini, Schistosoma mekongi and soil-transmitted helminthes on the Mekong Islands, Southern Lao PDR. *Infectious Diseases of Poverty.* 2017;6(1):1-15.

154. Yu W, Ross AG, Olveda RM, et al. Risk of human helminthiases: geospatial distribution and targeted control. *International Journal of Infectious Diseases.* 2017;55:131-138.

155. Ya-Lan Z, Yan-Kun Z, Wei-Qi C, et al. Survey and analysis of epidemic status of principal human parasitosis in ecological region of Huaiyang hills of Henan Province in 2015. *Zhongguo xue xi Chong Bing Fang zhi za zhi= Chinese Journal of Schistosomiasis Control.* 2017;29(5):607-611.

156. Mationg MLS, Gordon CA, Tallo VL, et al. Status of soil-transmitted helminth infections in schoolchildren in Laguna Province, the Philippines: Determined by parasitological and molecular diagnostic techniques. *PLoS neglected tropical diseases.* 2017;11(11):e0006022.

157. Shan-Shan L, Fei L, Jun X, Yi Y. Survey and analysis of major human parasitic diseases in Chongqing City. *Chinese Journal of Schistosomiasis Control.* 2018;30(2):194.

158. Yang D, Yang Y, Wang Y, et al. Prevalence and risk factors of Ascaris lumbricoides, Trichuris trichiura and Cryptosporidium infections in elementary school children in southwestern China: a school-based cross-sectional study. *International Journal of Environmental Research and Public Health.* 2018;15(9):1809.

159. Mohd-Shaharuddin N, Lim YAL, Hassan NA, Nathan S, Ngui R. Soil-transmitted helminthiasis among indigenous communities in Malaysia: Is this the endless malady with no solution. *Tropical Biomedicine.* 2018;35(1):168-180.

160. Suntaravitun P, Dokmaikaw A. Prevalence of intestinal parasites and associated risk factors for infection among rural communities of Chachoengsao Province, Thailand. *The Korean journal of parasitology.* 2018;56(1):33.

161. Shou-Jia X, Rong L, Qin-Ping Z, Hong-Ying Z, Wan-Hong Y, Hui-Fen D. Investigation on intestinal nematode infections and their risk factors in Shanpo Sub-district, Jiangxia District, Wuhan City. *Zhongguo xue xi Chong Bing Fang zhi za zhi= Chinese Journal of Schistosomiasis Control.* 2018;30(5):567-570.

162. Yanola J, Nachaiwieng W, Duangmano S, Prasannarong M, Somboon P, Pornprasert S. Current prevalence of intestinal parasitic infections and their impact on hematological and nutritional status among Karen hill tribe children in Omkoi District, Chiang Mai Province, Thailand. *Acta tropica.* 2018;180:1-6.

163. de Gier B, Pita-Rodríguez GM, Campos-Ponce M, et al. Soil-transmitted helminth infections and intestinal and systemic inflammation in schoolchildren. *Acta tropica.* 2018;182:124-127.

164. Long-Zhi J. Epidemic status of human key parasitic diseases in Tongcheng City, Anhui Province. *Zhongguo xue xi Chong Bing Fang zhi za zhi= Chinese Journal of Schistosomiasis Control.* 2017;30(1):68-71.

165. Li T, Chen X, Wang H, et al. High prevalence of taeniasis and Taenia solium cysticercosis in children in western Sichuan, China. *Acta tropica.* 2019;199:105133.

166. Nasution RKA, Nasution BB, Lubis M, Lubis IND. Prevalence and knowledge of soil-transmitted helminth infections in Mandailing Natal, North Sumatera, Indonesia. *Open Access Macedonian Journal of Medical Sciences.* 2019;7(20):3443.

167. Chard AN, Baker KK, Tsai K, et al. Associations between soil-transmitted helminthiasis and viral, bacterial, and protozoal enteroinfections: a cross-sectional study in rural Laos. *Parasites & vectors.* 2019;12(1):1-11.

168. Dai Y, Xu X, Liu J, et al. Prevalence of intestinal helminth infections in Jiangsu Province, eastern China; a cross-sectional survey conducted in 2015. *BMC infectious diseases.* 2019;19(1):1-9.

169. Han KT, Wai KT, Aye KH, Kyaw KW, Maung WP, Oo T. Emerging neglected helminthiasis and determinants of multiple helminth infections in flood-prone township in Myanmar. *Tropical medicine and health.* 2019;47(1):1-10.

170. JingXiao Z, YuFang L, ShiLei C, et al. Endemic status of human common parasite infections in Qinghai Province in 2015. *Chinese Journal of Parasitology and Parasitic Diseases.* 2019;37(2):178-182.

171. Muslim A, Mohd Sofian S, Shaari SA, Hoh B-P, Lim YA-L. Prevalence, intensity and associated risk factors of soil transmitted helminth infections: A comparison between Negritos (indigenous) in inland jungle and those in resettlement at town peripheries. *PLoS neglected tropical diseases.* 2019;13(4):e0007331.

172. Adli MN, Ghani MKA. Soil Transmitted Helminthiases amongst the Orang Asli (aborigine) Children of SKTAR Kuala Kubu Bharu, Selangor, Malaysia. *International Medical Journal.* 2020;27(1):21-23.

173. Kurscheid J, Laksono B, Park M, et al. Epidemiology of soil-transmitted helminth infections in Semarang, Central Java, Indonesia. *PLoS neglected tropical diseases.* 2020;14(12):e0008907.

174. Lim-Leroy A, Chua TH. Prevalence and risk factors of geohelminthiasis among the rural village children in Kota Marudu, Sabah, Malaysia. *PLoS One.* 2020;15(9):e0239680.

175. Nasr NA, Al-Mekhlafi HM, Lim YA, et al. A holistic approach is needed to control the perpetual burden of soil-transmitted helminth infections among indigenous schoolchildren in Malaysia. *Pathogens and Global Health.* 2020;114(3):145-159.

176. Adisakwattana P, Yoonuan T, Phuphisut O, et al. Clinical helminthiases in Thailand border regions show elevated prevalence levels using qPCR diagnostics combined with traditional microscopic methods. *Parasites & vectors.* 2020;13(1):1-10.

177. Chai J-Y, Sohn W-M, Hong S-J, et al. Effect of mass drug administration with a single dose of albendazole on Ascaris lumbricoides and Trichuris trichiura infection among schoolchildren in Yangon Region, Myanmar. *The Korean Journal of Parasitology.* 2020;58(2):195.

178. Li S-S, Wang L, Li A-H. Surveillance of soil-transmitted nematode infections in Zhenjiang City from 2006 to 2018. *Zhongguo xue xi chong bing fang zhi za zhi= Chinese journal of schistosomiasis control.* 2019;32(1):83-86.

179. Subahar R, Susanto L, Astuty H, Winita R, Sari IP. Intestinal Parasitic Infections and Hemoglobin Levels among Schoolchildren Participating in a Deworming Program in Jakarta, Indonesia: A Cross-Sectional Study. *Open Access Macedonian Journal of Medical Sciences.* 2020;8(E):589-594.

180. Wandra T, Darlan DM, Yulfi H, et al. Soil-transmitted helminth infections and taeniasis on Samosir Island, Indonesia. *Acta Tropica.* 2020;202:105250.

181. Zeng W, Malla P, Xu X, et al. Associations among Soil-Transmitted Helminths, G6PD Deficiency and Asymptomatic Malaria Parasitemia, and Anemia in Schoolchildren from a Conflict Zone of Northeast Myanmar. *The American Journal of Tropical Medicine and Hygiene.* 2020;102(4):851.

182. Zhu H-H, Zhou C-H, Zhu T-J, et al. Prevalence of soil-borne nematode infections among residents living in urban/town areas of China in 2015. *Zhongguo xue xi Chong Bing Fang zhi za zhi= Chinese Journal of Schistosomiasis Control.* 2020;32(5):476-482.

183. Zhu H-H, Huang J-L, Zhu T-J, et al. National surveillance on soil-transmitted helminthiasis in the People's Republic of China. *Acta Tropica.* 2020;205:105351.

184. Labana RV, Romero VA, Guinto AM, et al. Prevalence and intensity of soil-transmitted helminth infections among school-age children in the Cagayan Valley, the Philippines. *Asian Pacific Journal of Tropical Medicine.* 2021;14(3):113.

185. Wattanawong O, Iamsirithaworn S, Kophachon T, et al. Current status of helminthiases in Thailand: A cross-sectional, nationwide survey, 2019. *Acta Tropica.* 2021;223:106082.

186. Wong LW, Ong KS, Goh CBS, et al. Extremely low prevalence in soil-transmitted helminth infections among a multi-ethnic community in Segamat, Malaysia. *Journal of Parasitic Diseases.* 2021;45(2):313-318.

187. Aung E, Han KT, Gordon CA, et al. High prevalence of soil-transmitted helminth infections in Myanmar schoolchildren. *Infectious Diseases of Poverty.* 2022;11(1):1-12.

188. Bria M, Arwati H, Tantular IS. Prevalence and risk factors of Ascaris lumbricoides infection in children of Manusak Village, Kupang District, East Nusa Tenggara Province, Indonesia. *Qanun Medika-Medical Journal Faculty of Medicine Muhammadiyah Surabaya.* 2021;5(2).

189. Djuardi Y, Lazarus G, Stefanie D, Fahmida U, Ariawan I, Supali T. Soil-transmitted helminth infection, anemia, and malnutrition among preschool-age children in Nangapanda subdistrict, Indonesia. *PLoS neglected tropical diseases.* 2021;15(6):e0009506.

190. Feng Y, Yu K, Chen H, et al. Soil-transmitted helminths, intestinal protozoa and Clonorchis sinensis infections in southeast China. *BMC infectious diseases.* 2021;21(1):1-11.

191. Jin H, Ryu K, Lee D, et al. Prevalence and Risk Factors of Intestinal Helminthiasis in Remote Mountainous Villages of Northern Lao PDR: A Cross-Sectional Study. *The Korean Journal of Parasitology.* 2021;59(2):131.

192. Mationg MLS, Williams GM, Tallo VL, et al. “The Magic Glasses Philippines”: a cluster randomised controlled trial of a health education package for the prevention of intestinal worm infections in schoolchildren. *The Lancet Regional Health-Western Pacific.* 2022;18:100312.

193. Devera R, Requena I, Blanco Y, Rumhein F, Velasquez V, Tedesco RM. Prevalence of intestinal parasites in school children from Escuela Básica Estadal José Félix Blanco, Bolívar State, Venezuela. *Salus.* 2010;14:43-48.

194. Santos SAd, Merlini LS. Prevalência de enteroparasitoses na população do município de Maria Helena, Paraná. *Ciência & Saúde Coletiva.* 2010;15:899-905.

195. Melo GC, Reyes-Lecca RC, Vitor-Silva S, et al. Concurrent helminthic infection protects schoolchildren with Plasmodium vivax from anemia. *PLoS One.* 2010;5(6):e11206.

196. Vidal S, Toloza L, Cancino B. Evolución de la prevalencia de enteroparasitosis en la ciudad de Talca, Región del Maule, Chile. *Revista chilena de infectología.* 2010;27(4):336-340.

197. Kurup R, Hunjan GS. Epidemiology and control of Schistosomiasis and other intestinal parasitic infections among school children in three rural villages of south Saint Lucia. *Journal of Vector Borne Diseases.* 2010;47(4):228.

198. Fonseca EOL, Teixeira MG, Barreto ML, Carmo EH, Costa MdCN. Prevalence and factors associated with geohelminth infections in children living in municipalities with low HDI in North and Northeast Brazil. *Cadernos de Saúde Pública.* 2010;26:143-152.

199. Mascarini-Serra LM, Telles CA, Prado MS, et al. Reductions in the prevalence and incidence of geohelminth infections following a city-wide sanitation program in a Brazilian Urban Centre. *PLoS Neglected Tropical Diseases.* 2010;4(2):e588.

200. Rosewell A, Robleto G, Rodrí, et al. Soil-transmitted helminth infection and urbanization in 880 primary school children in Nicaragua, 2005. *Tropical doctor.* 2010;40(3):141-143.

201. Silva JC, Furtado LFV, Ferro TC, Bezerra KdC, Borges EP, Melo ACFL. Parasitismo por Ascaris lumbricoides e seus aspectos epidemiológicos em crianças do Estado do Maranhão. *Revista da Sociedade Brasileira de Medicina Tropical.* 2011;44:100-102.

202. Molina N, Pezzani B, Ciarmela M, et al. Intestinal parasites and genotypes of Giardia intestinalis in school children from Berisso, Argentina. *The Journal of Infection in Developing Countries.* 2011;5(07):527-534.

203. Monárrez-Espino J, Pérez-Espejo CR, Vázquez-Mendoza G, Balleza-Carreón A, Caballero-Hoyos R. Intervention to prevent intestinal parasitic reinfections among Tarahumara indigenous schoolchildren in northern Mexico. *Revista Panamericana de Salud Pública.* 2011;30:196-203.

204. Vasconcelos IAB, Oliveira JW, Cabral FRF, Coutinho HDM, Menezes IRA. Prevalência de parasitoses intestinais entre crianças de 4-12 anos no Crato, Estado do Ceará: um problema recorrente de saúde pública. *Acta Scientiarum Health Sciences.* 2011;33(1):35-41.

205. Valverde JG, Gomes-Silva A, De Carvalho Moreira C, et al. Prevalence and epidemiology of intestinal parasitism, as revealed by three distinct techniques in an endemic area in the Brazilian Amazon. *Ann Trop Med Parasitol.* 2011;105(6):413-424.

206. Cañete R, Díaz MM, Avalos García R, Laúd Martinez PM, Manuel Ponce F. Intestinal parasites in children from a day care centre in Matanzas City, Cuba. *PLoS One.* 2012;7(12):e51394.

207. Cardona Arias JA, Bedoya Urrego K. Frecuencia de parásitos intestinales y evaluación de métodos para su diagnóstico en una comunidad marginal de Medellín, Colombia. *Iatreia.* 2013;26(3):257-268.

208. Ines Gamboa M, Lorena Zonta M, Navone GT. The prevalence of geohelminthiasis is related to socio-environmental conditions. *SALUD I CIENCIA.* 2012;19(1):16-21.

209. Lander RL, Lander AG, Houghton L, et al. Factors influencing growth and intestinal parasitic infections in preschoolers attending philanthropic daycare centers in Salvador, Northeast Region of Brazil. *Cadernos de saude publica.* 2012;28:2177-2188.

210. de Rivero R Z, Calchi L, Acurero E, et al. Intestinal Protozoa and Helminths in Asymptomatic Adults in the State of Zulia State, Venezuela. *Kasmera.* 2012;40(2):186-194.

211. Alcantara-Neves NM, Veiga RV, Dattoli VCC, et al. The effect of single and multiple infections on atopy and wheezing in children. *Journal of Allergy and Clinical Immunology.* 2012;129(2):359-367. e353.

212. Branco N, Leal DAG, Franco RMB. A parasitological survey of natural water springs and inhabitants of a tourist city in southeastern Brazil. *Vector-Borne and Zoonotic Diseases.* 2012;12(5):410-417.

213. Carvalho GLXd, Moreira LE, Pena JL, Marinho CC, Bahia MT, Machado-Coelho GLL. A comparative study of the TF-Test®, Kato-Katz, Hoffman-Pons-Janer, Willis and Baermann-Moraes coprologic methods for the detection of human parasitosis. *Memórias do Instituto Oswaldo Cruz.* 2012;107:80-84.

214. Machicado JD, Marcos LA, Tello R, Canales M, Terashima A, Gotuzzo E. Diagnosis of soil-transmitted helminthiasis in an Amazonic community of Peru using multiple diagnostic techniques. *Transactions of the Royal Society of Tropical Medicine and Hygiene.* 2012;106(6):333-339.

215. Quintero K, Durán C, Duri D, et al. Household social determinants of ascariasis and trichuriasis in North Central Venezuela. *International health.* 2012;4(2):103-110.

216. Damazio SM, Lima MdS, Soares AR, Souza MAAd. Intestinal parasites in a quilombola community of the Northern State of Espírito Santo, Brazil. *Revista do Instituto de Medicina Tropical de São Paulo.* 2013;55:179-183.

217. Gutierrez-Jimenez J, Torres-Sanchez MG, Fajardo-Martinez LP, et al. Malnutrition and the presence of intestinal parasites in children from the poorest municipalities of Mexico. *The Journal of Infection in Developing Countries.* 2013;7(10):741-747.

218. Nobre LN, Silva RV, Macedo MS, Teixeira RA, Lamounier JA, Franceschini SC. Risk factors for intestinal parasitic infections in preschoolers in a low socio-economic area, Diamantina, Brazil. *Pathogens and global health.* 2013;107(2):103-106.

219. Verhagen LM, Incani RN, Franco CR, et al. High malnutrition rate in Venezuelan Yanomami compared to Warao Amerindians and Creoles: significant associations with intestinal parasites and anemia. *PLoS One.* 2013;8(10):e77581.

220. Gutiérrez EJ, Pineda V, Calzada JE, et al. Enteric parasites and enteroaggregative Escherichia coli in children from Cañazas County, Veraguas Province, Panama. *The American journal of tropical medicine and hygiene.* 2014;91(2):267.

221. Muñoz-Antoli C, Pavón A, Marcilla A, Toledo R, Esteban J. Prevalence and risk factors related to intestinal parasites among children in Department of Rio San Juan, Nicaragua. *Transactions of the Royal Society of Tropical Medicine and Hygiene.* 2014;108(12):774-782.

222. Alcântara-Neves NM, de SG Britto G, Veiga RV, et al. Effects of helminth co-infections on atopy, asthma and cytokine production in children living in a poor urban area in Latin America. *BMC Research Notes.* 2014;7(1):1-11.

223. Bragagnoli G, Silva MTN. Ascaris lumbricoides infection and parasite load are associated with asthma in children. *The Journal of Infection in Developing Countries.* 2014;8(07):891-897.

224. Cepon-Robins TJ, Liebert MA, Gildner TE, et al. Soil-transmitted helminth prevalence and infection intensity among geographically and economically distinct Shuar communities in the Ecuadorian Amazon. *The Journal of Parasitology.* 2014;100(5):598-607.

225. Gabrie JA, Rueda MM, Canales M, Gyorkos TW, Sanchez AL. School hygiene and deworming are key protective factors for reduced transmission of soil-transmitted helminths among schoolchildren in Honduras. *Parasites & vectors.* 2014;7(1):1-15.

226. Mejia Torres RE, Franco Garcia DN, Fontecha Sandoval GA, et al. Prevalence and intensity of soil-transmitted helminthiasis, prevalence of malaria and nutritional status of school going children in Honduras. *PLoS neglected tropical diseases.* 2014;8(10):e3248.

227. Gamboa MI, Giambelluca LA, Navone GT. Distribución espacial de las parasitosis intestinales en la ciudad de La Plata, Argentina. *MEDICINA (Buenos Aires).* 2014;74(5):363-370.

228. Silva MTN, Santana JV, Bragagnoli G, Marinho AMdN, Malagueño E. Prevalence of Entamoeba histolytica/Entamoeba dispar in the city of Campina Grande, in northeastern Brazil. *Revista do Instituto de Medicina Tropical de São Paulo.* 2014;56:451-454.

229. Cimino RO, Jeun R, Juarez M, et al. Identification of human intestinal parasites affecting an asymptomatic peri-urban Argentinian population using multi-parallel quantitative real-time polymerase chain reaction. *Parasites & vectors.* 2015;8(1):1-7.

230. Alves de Oliveira Serra MA, Chaves CdS, Branco Coêlho ZC, et al. Comparison between two decades of prevalence of intestinal parasitic diseases and risk factors in a Brazilian urban centre. *Interdisciplinary perspectives on infectious diseases.* 2015;2015.

231. Dib JR, Fernandez Zenoff MV, Oquilla JdV, Lazarte S, Gonzalez SN. Prevalence of intestinal parasitic infection among children from a shanty town in Tucuman, Argentina. 2015.

232. Fernández-Niño JA, Ramírez JD, López MC, Moncada LI, Reyes P, Heredia RD. Agreement of the Kato-Katz test established by the WHO with samples fixed with sodium acetate analyzed at 6 months to diagnose intestinal geohelminthes. *Acta tropica.* 2015;146:42-44.

233. Periago MV, Diniz RC, Pinto SA, et al. The right tool for the job: detection of soil-transmitted helminths in areas co-endemic for other helminths. *PLoS neglected tropical diseases.* 2015;9(8):e0003967.

234. Rollemberg CV, Silva MM, Rollemberg KC, et al. Predicting frequency distribution and influence of sociodemographic and behavioral risk factors of Schistosoma mansoni infection and analysis of co-infection with intestinal parasites. *Geospatial health.* 2015;10(1).

235. Echazú A, Bonanno D, Juarez M, et al. Effect of poor access to water and sanitation as risk factors for soil-transmitted helminth infection: selectiveness by the infective route. *PLoS neglected tropical diseases.* 2015;9(9):e0004111.

236. Cabada MM, Goodrich MR, Graham B, et al. Prevalence of intestinal helminths, anemia, and malnutrition in Paucartambo, Peru. *Revista Panamericana de Salud Pública.* 2015;37:69-75.

237. Barra M, Bustos L, Ossa X. Desigualdad en la prevalencia de parasitosis intestinal en escolares de una escuela urbana y dos rurales de la comuna de Puerto Montt. *Revista médica de Chile.* 2016;144(7):886-893.

238. Bouwmans MC, Gaona MA, Chenault MN, Zuluaga C, Pinzón-Rondon ÁM. Prevalence of intestinal parasitic infections in preschool-children from vulnerable neighborhoods in Bogotá. *Revista de la Universidad Industrial de Santander Salud.* 2016;48(2):178-187.

239. Casavechia MTG, Lonardoni MVC, Venazzi EAS, et al. Prevalence and predictors associated with intestinal infections by protozoa and helminths in southern Brazil. *Parasitology research.* 2016;115(6):2321-2329.

240. Gonçalves AQ, Junqueira ACV, Abellana R, et al. Prevalence of intestinal parasites and risk factors forspecific and multiple helminth infections in a remote city of the Brazilian Amazon. *Revista da Sociedade Brasileira de Medicina Tropical.* 2016;49:119-124.

241. Macchioni F, Segundo H, Totino V, et al. Intestinal parasitic infections and associated epidemiological drivers in two rural communities of the Bolivian Chaco. 2016.

242. Macchioni F, Segundo H, Gabrielli S, et al. Dramatic decrease in prevalence of soil-transmitted helminths and new insights into intestinal protozoa in children living in the Chaco region, Bolivia. *The American journal of tropical medicine and hygiene.* 2015;92(4):794.

243. Da Silva JB, Bossolani GDP, Piva C, et al. Spatial distribution of intestinal parasitic infections in a Kaingang indigenous village from Southern Brazil. *International journal of environmental health research.* 2016;26(5-6):578-588.

244. Lopez M, Morales ML, Konana M, et al. Kato-Katz and Lumbreras rapid sedimentation test to evaluate helminth prevalence in the setting of a school-based deworming program. *Pathogens and global health.* 2016;110(3):130-134.

245. Cabada MM, Morales ML, Lopez M, et al. Hymenolepis nana impact among children in the highlands of Cusco, Peru: an emerging neglected parasite infection. *The American journal of tropical medicine and hygiene.* 2016;95(5):1031.

246. Choi B, Kim B. Prevalence and risk factors of intestinal parasite infection among schoolchildren in the peripheral highland regions of Huanuco, Peru. *Osong Public Health and Research Perspectives.* 2017;8(5):302.

247. Fonseca REPd, Barbosa MCR, Ferreira BR. High prevalence of enteroparasites in children from Ribeirão Preto, São Paulo, Brazil. *Revista brasileira de enfermagem.* 2017;70:566-571.

248. Incani RN, Ferrer E, Hoek D, et al. Diagnosis of intestinal parasites in a rural community of Venezuela: Advantages and disadvantages of using microscopy or RT-PCR. *Acta tropica.* 2017;167:64-70.

249. Muñoz-Antoli C, Pavón A, Pérez P, Toledo R, Esteban J. Soil-transmitted helminth infections in schoolchildren of Laguna de Perlas (Nicaragua). *Journal of Tropical Pediatrics.* 2017;63(2):124-134.

250. Faria CP, Zanini GM, Dias GS, et al. Geospatial distribution of intestinal parasitic infections in Rio de Janeiro (Brazil) and its association with social determinants. *PLoS neglected tropical diseases.* 2017;11(3):e0005445.

251. Seguí R, Klisiowicz D, Oishi CY, Toledo R, Esteban JG, Muñoz-Antoli C. Intestinal symptoms and Blastocystis load in schoolchildren of Paranaguá Bay, Paraná, Brazil. *Revista do Instituto de Medicina Tropical de São Paulo.* 2017;59.

252. Cañete R, Campos Y, Valdes R, Rodriguez P. Prevalence and factors associated with intestinal parasitic infection among schoolchildren from Jagüey Grande Municipality in Matanzas Province, Cuba. *West Indian Med J.* 2017;66(2):361-366.

253. Coronato-Nunes B, Calegar DA, Monteiro KJL, et al. Giardia intestinalis infection associated with malnutrition in children living in northeastern Brazil. *The Journal of Infection in Developing Countries.* 2017;11(07):563-570.

254. Ignacio CF, Silva MECd, Handam NB, et al. Socioenvironmental conditions and intestinal parasitic infections in Brazilian urban slums: a cross-sectional study. *Revista do Instituto de Medicina Tropical de São Paulo.* 2017;59.

255. Cociancic P, Zonta ML, Navone GT. A cross‐sectional study of intestinal parasitoses in dogs and children of the periurban area of La Plata (Buenos Aires, Argentina): Zoonotic importance and implications in public health. *Zoonoses and Public Health.* 2018;65(1):e44-e53.

256. Costa JdO, Resende JA, Gil FF, Santos JFG, Gomes MA. Prevalence of Entamoeba histolytica and other enteral parasitic diseases in the metropolitan region of Belo Horizonte, Brazil. A cross-sectional study. *São Paulo Medical Journal.* 2018;136:319-323.

257. Cruz-Cruz C, López-Hernández D, Hernández-Shilón JA, Luna-Cazáres LM, Vidal JE, Gutiérrez-Jiménez J. Stunting and intestinal parasites in school children from high marginalized localities at the Mexican southeast. *The Journal of Infection in Developing Countries.* 2018;12(11):1026-1033.

258. Giraldo-Ospina B, Fontal-Vargas P-A, López-Muñoz D-F, Beltrán-Angarita L, Morales-Jiménez V, Gómez M-N. Prevalence of intestinal parasites in children of an invasion community in a municipality of Colombia. 2018.

259. Muñoz-Antoli C, Gozalbo M, Pavón A, Pérez P, Toledo R, Esteban J-G. Enteroparasites in preschool children on the pacific region of Nicaragua. *The American journal of tropical medicine and hygiene.* 2018;98(2):570.

260. Periago MV, García R, Astudillo OG, Cabrera M, Abril MC. Prevalence of intestinal parasites and the absence of soil-transmitted helminths in Añatuya, Santiago del Estero, Argentina. *Parasites & vectors.* 2018;11(1):1-13.

261. Spinicci M, Macchioni F, Rojo D, et al. Scaling down of a deworming programme among school‐age children after a thirty‐year successful intervention in the Bolivian Chaco. *Trop Med Int Health.* 2018;23(6):616-621.

262. Moncayo AL, Lovato R, Cooper PJ. Soil-transmitted helminth infections and nutritional status in Ecuador: findings from a national survey and implications for control strategies. *BMJ open.* 2018;8(4):e021319.

263. Muñoz-Antoli C, Pérez P, Pavón A, Toledo R, Esteban J-G. Soil-transmitted helminth infections and anemia in schoolchildren from Corn Island Archipelago (RAAS, Nicaragua). *The American journal of tropical medicine and hygiene.* 2018;99(6):1591.

264. Seguí R, Muñoz-Antoli C, Klisiowicz DR, et al. Prevalence of intestinal parasites, with emphasis on the molecular epidemiology of Giardia duodenalis and Blastocystis sp., in the Paranaguá Bay, Brazil: a community survey. *Parasites & vectors.* 2018;11(1):1-19.

265. Barbosa CV, Barreto MM, Andrade RdJ, et al. Intestinal parasite infections in a rural community of Rio de Janeiro (Brazil): prevalence and genetic diversity of Blastocystis subtypes. *PLoS One.* 2018;13(3):e0193860.

266. Anselmi M, Guevara A, Vicuña Y, et al. Community epidemiology approach to parasitic infection screening in a remote community in Ecuador. *The American journal of tropical medicine and hygiene.* 2019;101(3):650.

267. de la Luz Galván-Ramírez M, Madriz-Elisondo AL, Ramírez CGT, Rameño JdJR, de la O Carrasco DA, López MAC. Enteroparasitism and risk factors associated with clinical manifestations in children and adults of Jalisco State in Western Mexico. *Osong Public Health and Research Perspectives.* 2019;10(1):39.

268. Pinzón-Rondon ÁM, Gaona MA, Bouwmans M, et al. Acceso a agua potable, protección ambiental y parasitismo intestinal infantil en El Codito. Bogotá, Colombia. *Revista de Salud Pública.* 2020;21:42-48.

269. Errea RA, Vasquez-Rios G, Calderon ML, et al. Soil-transmitted helminthiasis in children from a rural community taking part in a periodic deworming program in the Peruvian Amazon. *The American journal of tropical medicine and hygiene.* 2019;101(3):636.

270. Gutiérrez-Jiménez J, Luna-Cázares LM, Martínez-de la Cruz L, et al. Children from a rural region in The Chiapas Highlands, Mexico, show an increased risk of stunting and intestinal parasitoses when compared with urban children. *Boletín médico del Hospital Infantil de México.* 2019;76(1):18-26.

271. Hernández PC, Morales L, Chaparro-Olaya J, et al. Intestinal parasitic infections and associated factors in children of three rural schools in Colombia. A cross-sectional study. *PLoS One.* 2019;14(7):e0218681.

272. Mazariego Arana MÁ, Gaspar A, del Rocío M, Ramírez Aguilar FJ, Trujillo Vizuet MG. Prevalencia de parasitosis intestinal en niños de guarderías rurales en Chiapas. *Enfermedades Infecciosas y Microbiología.* 2020;40(2):43.

273. Cociancic P, Torrusio SE, Zonta ML, Navone GT. Risk factors for intestinal parasitoses among children and youth of Buenos Aires, Argentina. *One Health.* 2020;9:100116.

274. Harvey TV, Tang AM, da Paixao Sevá A, et al. Enteric parasitic infections in children and dogs in resource-poor communities in northeastern Brazil: Identifying priority prevention and control areas. *PLoS neglected tropical diseases.* 2020;14(6):e0008378.

275. Ibiapina AB, Leal JS, de Santana PRA, Mesquita MR, da Cunha Lopes TL, Braz DC. Enteroparasitosis in patients attended by the health public service: epidemiology and spatial distribution. *Scientia Medica.* 2020;30(1):e34764-e34764.

276. Gildner TE, Cepon-Robins TJ, Liebert MA, et al. Market integration and soil-transmitted helminth infection among the Shuar of Amazonian Ecuador. *PLoS One.* 2020;15(7):e0236924.

277. Naceanceno KS, Matamoros G, Gabrie JA, Bottazzi ME, Sanchez A, Mejia R. Use of multi-parallel real-time quantitative pcr to determine Blastocystis prevalence and association with other gastrointestinal parasite infection in a rural honduran location. *The American Journal of Tropical Medicine and Hygiene.* 2020;102(6):1373.

278. González Quiroz DJ, Agudelo Lopez SdP, Arango CM, et al. Prevalence of soil transmitted helminths in school-aged children, Colombia, 2012-2013. *PLoS neglected tropical diseases.* 2020;14(7):e0007613.

279. Falcone AC, Zonta ML, Unzaga JM, Navone GT. Parasitic risk factors in migrant horticultural families from Bolivia settled in the rural area of La Plata, Buenos Aires, Argentina. *One Health.* 2020;11:100179.

280. Aboikoni A, Allaire M, Louvel D, et al. Prevalence of intestinal parasite among patients attending two hospitals in French Guiana: A 6-year retrospective study. *PLoS neglected tropical diseases.* 2021;15(2):e0009087.

281. Cociancic P, Torrusio SE, Garraza M, Zonta ML, Navone GT. Intestinal parasites in child and youth populations of Argentina: Environmental factors determining geographic distribution. *Revista Argentina de Microbiología.* 2021;53(3):225-232.

282. de Morais NG, de Lima LFF, Leite IDS, et al. High prevalence of protozoan infections: a permanent cycle in a brazilian semi-arid peripheric area. *Revista de Patologia Tropical/Journal of Tropical Pathology.* 2021;50(3).

283. Incani RN, Grillet ME, Mughini-Gras L. Hotspots and correlates of soil-transmitted helminth infections in a Venezuelan rural community: Which are the “wormy” houses? *Journal of Infection.* 2021;82(1):143-149.

284. Andrade I, Granoble GM, Álava N, Leal BC. Prevalencia de parasitosis intestinal en escolares de 5 a 9 años del barrio Las Penas de la ciudad de Guayaquil 2020. *Boletín de Malariología y Salud Ambiental.* 2021;61(2):185-194.

285. Chura JA, Macchioni F, Furzi F, et al. Cross-sectional study on intestinal parasite infections in different ecological zones of the Department of La Paz, Bolivia. *One Health.* 2021;13:100271.

286. Bryan PE, Romero M, Sánchez M, et al. Urban versus Rural Prevalence of Intestinal Parasites Using Multi-Parallel qPCR in Colombia. *Am J Trop Med Hyg.* 2020;104(3):907-909.

287. Calegar DA, Bacelar PA, Monteiro KJ, et al. A community-based, cross-sectional study to assess interactions between income, nutritional status and enteric parasitism in two Brazilian cities: are we moving positively towards 2030? *Journal of Health, Population and Nutrition.* 2021;40(1):1-10.

288. Al-Haddad A, Baswaid S. Frequency of intestinal parasitic infection among children in Hadhramout governorate (Yemen). *J Egypt Soc Parasitol.* 2010;40(2):479-488.

289. Bdir S, Adwan G. Prevalence of intestinal parasitic infections in Jenin Governorate, Palestine: a 10–year retrospective study. *Asian Pacific Journal of Tropical Medicine.* 2010;3(9):745-747.

290. Al-Mohammed HI, Amin TT, Aboulmagd E, Hablus HR, Zaza BO. Prevalence of intestinal parasitic infections and its relationship with socio–demographics and hygienic habits among male primary schoolchildren in Al–Ahsa, Saudi Arabia. *Asian Pacific Journal of Tropical Medicine.* 2010;3(11):906-912.

291. Dash N, Al-Zarouni M, Anwar K, Panigrahi D. Prevalence of intestinal parasitic infections in Sharjah, United Arab Emirates. *Human Parasitic Diseases.* 2010;2:21.

292. Köksal F, Başlanti I, Samasti M. A retrospective evaluation of the prevalence of intestinal parasites in Istanbul, Turkey. *Turkiye Parazitol Derg.* 2010;34(3):166-171.

293. Alyousefi NA, Mahdy MA, Mahmud R, Lim YA. Factors associated with high prevalence of intestinal protozoan infections among patients in Sana'a City, Yemen. *PLoS One.* 2011;6(7):e22044.

294. Araj GF, Musharrafieh UM, Haydar A, Ghawi A, Itani R, Saliba R. Trends and prevalence of intestinal parasites at a tertiary care center in Lebanon over a decade. *Le Journal Medical libanais The Lebanese Medical Journal.* 2011;59(3):143-148.

295. Calik S, Karaman U, Colak C. Prevalence of microsporidium and other intestinal parasites in children from Malatya, Turkey. *Indian journal of microbiology.* 2011;51(3):345-349.

296. Ekinci B, Karacaoğlan E, Bulucu E, Sül N. Muğla ili merkez ilköğretim okulu öğrencilerinde bağırsak parazitleri araştırılması. *Türkiye Parazitol Derg.* 2011;35(2):92-95.

297. El Guamri Y, Belghyti D, Barkia A, et al. Parasitic infection of the digestive tract in children in a regional hospital center in Gharb (Kenitra, Morroco): some epidemiological features. *East African Journal of Public Health.* 2011;8(4):250-257.

298. Hussein RA, Shaker MJ, Majeed HA. Prevalence of intestinal parasitic infections among children in Baghdad City. *Journal of College of Basic Education.* 2011;71:130-147.

299. Hussein AS. Prevalence of intestinal parasites among school children in northern districts of West Bank‐Palestine. *Trop Med Int Health.* 2011;16(2):240-244.

300. Ibrahium FA. Prevalence and predisposing factors regarding intestinal parasitic infections among rural primary school pupils at Minia Governorate, Egypt. *Journal of Public Health in Africa.* 2011;2(2).

301. Al-Kubaisy W, Al-Talib H, Rajih A, Shanshal M. Intestinal Parasitic Diarrhea among Children in Baghdad – Iraq. *Tropical Biomedicine.* 2014;31:499-506.

302. Farghly AM, Mohamed S, Abdel-Rahman SA, Mohammed FE, El-Bahaie ES, El-Shafey MA. The relation between the prevalence of soil transmitted parasites in the soil and among school children in Zagazig district, Sharkyia Governorate, Egypt. *Journal of Parasitic Diseases.* 2016;40(3):1021-1029.

303. Hegazy AM, Younis NT, Aminou HA, Badr AM. Prevalence of intestinal parasites and its impact on nutritional status among preschool children living in Damanhur City, El-Behera Governorate, Egypt. *Journal of the Egyptian Society of Parasitology.* 2014;44(2):517-524.

304. Uysal HK, Akgül Ö, Purisa S, Öner YA. İstanbul Üniversitesi İstanbul Tıp Fakültesi’nde 25 Yıllık İntestinal Parazit Prevalansı: Retrospektif Bir Çalışma. *Turkiye Parazitol Derg.* 2014;38:97-101.

305. Aytar AA, Öztürk EC, Göçmen Ş, et al. Öğrencilerde bağırsak parazitlerinin ve hijyen bilgi düzeylerinin araştırılması. 2015.

306. Doni NY, Gurses G, Simsek Z, Zeyrek FY. Prevalence and associated risk factors of intestial parasites among children of farm workers in the southeastern Anatolian region of Turkey. *Annals of Agricultural and Environmental Medicine.* 2015;22(3).

307. Al-Mekhlafi AM, Abdul-Ghani R, Al-Eryani SM, Saif-Ali R, Mahdy MA. School-based prevalence of intestinal parasitic infections and associated risk factors in rural communities of Sana'a, Yemen. *Acta tropica.* 2016;163:135-141.

308. Alsubaie ASR, Azazy AA, Omer EO, Al-Shibani LA, Al-Mekhlafi AQ, Al-Khawlani FA. Pattern of parasitic infections as public health problem among school children: A comparative study between rural and urban areas. *Journal of Taibah University Medical Sciences.* 2016;11(1):13-18.

309. Amer OH, Ashankyty IM, Haouas NAS. Prevalence of intestinal parasite infections among patients in local public hospitals of Hail, Northwestern Saudi Arabia. *Asian Pacific journal of tropical medicine.* 2016;9(1):44-48.

310. Jaran A. Prevalence and seasonal variation of human intestinal parasites in patients attending hospital with abdominal symptoms in northern Jordan. *EMHJ-Eastern Mediterranean Health Journal.* 2016;22(10):756-760.

311. Alwabr GM, Al-Moayed EE. Prevalence of intestinal parasitic infections among school children of Al-Mahweet Governorate, Yemen. *European Journal of Biological Research.* 2016;6(2):64-73.

312. Gashout A, Taweni F, Elmabrouk H. Pattern of intestinal parasites among hospital patients at Tripoli Central Hospital, Libya. *Libyan Journal of Medical Sciences.* 2017;1(1):13.

313. Musa IS. Incidence of helminthiasis in humans in Iraq. *Karbala International Journal of Modern Science.* 2017;3(4):267-271.

314. Saheb EJ, Mahdi SG, Mosa IS, Abdul-Karim MI, Khistawi AN. Epidemiology of Some Parasitic Helminthes in Iraq from 2011 until 2015. *Iraqi Journal of Science.* 2017;58(2B):789-796.

315. Alharbi RA, Alwajeeh TS, Assabri AM, Almalki SS, Alruwetei A, Azazy AA. Intestinal parasitoses and schistosome infections among students with special reference to praziquantel efficacy in patients with schistosomosis in Hajjah governorate, Yemen. *Annals of Parasitology.* 2019;65(3).

316. Cengiz ZT, Yılmaz H, Beyhan YE, Çiçek M. A comprehensive retrospective study: intestinal parasites in human in Van Province. *Türkiye Parazitolojii Dergisi.* 2019;43(2):70.

317. Al-Saqur IM, Al-Warid HS, Al-Qaisi AQ, Al-Bahadely HS. Prevalence of gastrointestinal parasites in Iraq during 2015. Paper presented at: AIP Conference Proceedings2020.

318. Polat E, Özdemir S, Sirekbasan S. The Distribution of Intestinal Parasites in Patients Presenting to a University Hospital in Istanbul: A Seven-year Retrospective Analysis. *Turkiye Parazitol Derg.* 2020;44(3):139-142.

319. Hamarsheh O, Amro A. Epidemiology of parasitic infections in the West Bank and Gaza Strip, Palestine. *The American Journal of Tropical Medicine and Hygiene.* 2020;102(2):313.

320. Al-Rifai RH, Loney T, Sheek-Hussein M, et al. Prevalence of, and factors associated with intestinal parasites in multinational expatriate workers in Al Ain City, United Arab Emirates: An occupational cross-sectional study. *Journal of immigrant and minority health.* 2020;22(2):359-374.

321. Al-Hindi A, Redwan AA, El-Egla GO, Qassem RRA, Alshammari A. Prevalence of intestinal parasitic infections among university female students, Gaza, Palestine. *Avicenna journal of medicine.* 2019;9(04):143-147.

322. Shehab AY, Allam AF, Farag HF, Elhadad H, Kotb SFE, El-Taweel HA. Intestinal parasites among humans and their livestock animals in a rural community in Gharbia governorate, Egypt. *Journal of Parasitic Diseases.* 2021;45(1):96-100.

323. Allam AF, Farag HF, Lotfy W, Fawzy HH, Elhadad H, Shehab AY. Comparison among FLOTAC, Kato-Katz and formalin ether concentration techniques for diagnosis of intestinal parasitic infections in school children in an Egyptian rural setting. *Parasitology.* 2021;148(3):289-294.

324. Shield JM, Kow F. A comparative study of intestinal helminths in pre-school-age urban and rural children in Morobe Province, Papua New Guinea. *Papua New Guinea Medical Journal.* 2013;56(1/2):14-31.

325. Bradbury RS, Harrington H, Kekeubata E, et al. High prevalence of ascariasis on two coral atolls in the Solomon Islands. *Transactions of The Royal Society of Tropical Medicine and Hygiene.* 2018;112(4):193-199.

326. Lee J-D, Yen C-M, Wang J-J, Lin R-J, Chung L-Y. A school-based soil-transmitted helminths survey in the Guadalcanal Province, the Solomon Islands. *Tropical Doctor.* 2021;51(2):167-170.

327. Abdel-Aziz MA, Afifi AA, Malik EM, Adam I. Intestinal protozoa and intestinal helminthic infections among schoolchildren in Central Sudan. *Asian Pacific Journal of Tropical Medicine.* 2010;3(4):292-293.

328. Ezeagwuna DA, Okwelogu I, Ekejindu I, Ogbuagu C. The prevalence and socio-economic factors of intestinal helminth infections among primary school pupils in Ozubulu, Anambra State, Nigeria. 2010;9.

329. Houmsou R, Amuta E, Olusi T. Prevalence of intestinal parasites among primary school children in Makurdi, Benue State-Nigeria. *The Internet Journal of Infectious Diseases.* 2010;8(1):80-86.

330. Jombo GT, Damen JG, Safiyanu H, Odey F, Mbaawuaga EM. Human intestinal parasitism, potable water availability and methods of sewage disposal among nomadic Fulanis in Kuraje rural settlement of Zamfara state. *Asian Pacific Journal of Tropical Medicine.* 2010;3(6):491-493.

331. Mazigo HD, Waihenya R, Lwambo NJ, et al. Co-infections with Plasmodium falciparum, Schistosoma mansoni and intestinal helminths among schoolchildren in endemic areas of northwestern Tanzania. *Parasites & vectors.* 2010;3(1):1-7.

332. Clements AC, Deville MA, Ndayishimiye O, Brooker S, Fenwick A. Spatial co‐distribution of neglected tropical diseases in the East African Great Lakes region: revisiting the justification for integrated control. *Trop Med Int Health.* 2010;15(2):198-207.

333. Sahr F, Gevao S, Bockarie A, et al. Prevalence and Intensity of Intestinal Helminth Parasites and Their Response to Treatment with Albendazole in a Rural Community in Sierra Leone. *Sierra Leone Journal of Biomedical Research.* 2010;2(2).

334. Knopp S, Mohammed KA, Stothard JR, et al. Patterns and risk factors of helminthiasis and anemia in a rural and a peri-urban community in Zanzibar, in the context of helminth control programs. *PLoS neglected tropical diseases.* 2010;4(5):e681.

335. Koroma JB, Peterson J, Gbakima AA, et al. Geographical distribution of intestinal schistosomiasis and soil-transmitted helminthiasis and preventive chemotherapy strategies in Sierra Leone. *PLoS neglected tropical diseases.* 2010;4(11):e891.

336. Midzi N, Mtapuri-Zinyowera S, Mapingure M, et al. Consequences of polyparasitism on anaemia among primary school children in Zimbabwe. *Acta tropica.* 2010;115(1-2):103-111.

337. Siwila J, Phiri IG, Enemark HL, Nchito M, Olsen A. Intestinal helminths and protozoa in children in pre-schools in Kafue district, Zambia. *Transactions of the Royal Society of Tropical Medicine and Hygiene.* 2010;104(2):122-128.

338. Sowemimo O, Asaolu S. Current status of soil-transmitted helminthiases among pre-school and school-aged children from Ile-Ife, Osun State, Nigeria. *Journal of helminthology.* 2011;85(3):234-238.

339. Oninla S, Onayade A, Owa J. Impact of intestinal helminthiases on the nutritional status of primary-school children in Osun state, south–western Nigeria. *Ann Trop Med Parasitol.* 2010;104(7):583-594.

340. Damen J, Luka J, Biwan E, Lugos M. Prevalence of intestinal parasites among pupils in rural North Eastern, Nigeria. *Nigerian medical journal: journal of the Nigeria Medical Association.* 2011;52(1):4.

341. Ejima L, Ajogun R. The prevalence and health implications of the ova of human intestinal helminth parasites isolated from faeces collected near students’ hostels, federal polytechnic, Idah, Kogi State, Nigeria. *Int J Trop Med.* 2011;6:15-18.

342. Idowu OA, Babatunde O, Soniran T, Adediran A. Parasitic infections in finger-sucking school age children. *The Pediatric infectious disease journal.* 2011;30(9):791-792.

343. Kamga HLF, Nsagha DS, Atanga MBS, et al. The impact of health education on the prevalence of faecal-orally transmitted parasitic infections among school children in a rural community in Cameroon. *Pan African Medical Journal.* 2011;8(1).

344. Osazuwa F, Ayo OM, Imade P. A significant association between intestinal helminth infection and anaemia burden in children in rural communities of Edo state, Nigeria. *North American journal of medical sciences.* 2011;3(1):30.

345. Alemu A, Atnafu A, Addis Z, et al. Soil transmitted helminths and Schistosoma mansoni infections among school children in Zarima town, northwest Ethiopia. *BMC infectious diseases.* 2011;11(1):1-7.

346. Fürst T, Müller I, Coulibaly JT, Yao AK, Utzinger J, N'Goran EK. Questionnaire-based approach to assess schoolchildren's physical fitness and its potential role in exploring the putative impact of helminth and Plasmodium spp. infections in Côte d'Ivoire. *Parasites & vectors.* 2011;4(1):1-10.

347. Green HK, Sousa-Figueiredo JC, Basanez M-G, et al. Anaemia in Ugandan preschool-aged children: the relative contribution of intestinal parasites and malaria. *Parasitology.* 2011;138(12):1534-1545.

348. Halwindi H, Magnussen P, Siziya S, Handema R, Meyrowitsch DW, Olsen A. Impact of community-directed treatment on soil transmitted helminth infections in children aged 12 to 59 months in Mazabuka District, Zambia. *Parasitology.* 2011;138(12):1578-1585.

349. Hodges M, Koroma MM, Baldé MS, et al. Current status of schistosomiasis and soil-transmitted helminthiasis in Beyla and Macenta Prefectures, Forest Guinea. *Transactions of the Royal Society of Tropical Medicine and Hygiene.* 2011;105(11):672-674.

350. Kabatereine NB, Standley CJ, Sousa-Figueiredo JC, et al. Integrated prevalence mapping of schistosomiasis, soil-transmitted helminthiasis and malaria in lakeside and island communities in Lake Victoria, Uganda. *Parasites & vectors.* 2011;4(1):1-14.

351. Midzi N, Mtapuri-Zinyowera S, Sangweme D, et al. Efficacy of integrated school based de-worming and prompt malaria treatment on helminths-Plasmodium falciparum co-infections: A 33 months follow up study. *BMC international health and human rights.* 2011;11(1):1-14.

352. Odiere MR, Opisa S, Odhiambo G, et al. Geographical distribution of schistosomiasis and soil-transmitted helminths among school children in informal settlements in Kisumu City, Western Kenya. *Parasitology.* 2011;138(12):1569-1577.

353. Ojurongbe O, Adegbayi AM, Bolaji OS, Akindele AA, Adefioye OA, Adeyeba OA. Asymptomatic falciparum malaria and intestinal helminths co-infection among school children in Osogbo, Nigeria. *Journal of research in medical sciences: the official journal of Isfahan University of Medical Sciences.* 2011;16(5):680.

354. Richardson DJ, Richardson KR, Callahan KD, et al. Geohelminth infection in rural Cameroonian villages. *Comparative Parasitology.* 2011;78(1):161-179.

355. Assob J, Nde P, Nsagha D, et al. The incidence of feco-oral parasites in street-food vendors in Buea, south-west region Cameroon. *African health sciences.* 2012;12(3):376-380.

356. Ayalew A, Debebe T, Worku A. Prevalence and risk factors of intestinal parasites among Delgi school children, North Gondar, Ethiopia. *Journal of Parasitology and Vector Biology.* 2011;3(5):75-81.

357. Ignatius R, Gahutu JB, Klotz C, et al. High prevalence of Giardia duodenalis Assemblage B infection and association with underweight in Rwandan children. *PLoS neglected tropical diseases.* 2012;6(6):e1677.

358. Mbuh J, Ntonifor N, Ojong J. The epidemiology of soil-transmitted helminth and protozoan infections in south-west Cameroon. *Journal of helminthology.* 2012;86(1):30-37.

359. Albonico M, Ame S, Vercruysse J, Levecke B. Comparison of the Kato-Katz thick smear and McMaster egg counting techniques for monitoring drug efficacy against soil-transmitted helminths in schoolchildren on Pemba Island, Tanzania. *Transactions of the Royal Society of Tropical Medicine and Hygiene.* 2012;106(3):199-201.

360. Bechir M, Schelling E, Hamit M, Tanner M, Zinsstag J. Parasitic infections, anemia and malnutrition among rural settled and mobile pastoralist mothers and their children in Chad. *EcoHealth.* 2012;9(2):122-131.

361. Dorkenoo A, Bronzan R, Ayena K, et al. Nationwide integrated mapping of three neglected tropical diseases in Togo: countrywide implementation of a novel approach. *Trop Med Int Health.* 2012;17(7):896-903.

362. Friedman AJ, Ali SM, Albonico M. Safety of a new chewable formulation of mebendazole for preventive chemotherapy interventions to treat young children in countries with moderate-to-high prevalence of soil transmitted helminth infections. *Journal of tropical medicine.* 2012;2012.

363. Hodges MH, Dada N, Warmsley A, et al. Mass drug administration significantly reduces infection of Schistosoma mansoni and hookworm in school children in the national control program in Sierra Leone. *BMC infectious diseases.* 2012;12(1):1-8.

364. Nwaneri D, Omuemu V. Prevalence and intensity of intestinal helminthiasis in children living in orphanages in Benin City, Nigeria. *J prev med hyg.* 2013;53(3):146-151.

365. Odiere MR, Rawago FO, Ombok M, et al. High prevalence of schistosomiasis in Mbita and its adjacent islands of Lake Victoria, western Kenya. *Parasites & vectors.* 2012;5(1):1-8.

366. Tchuem Tchuenté L-A, Kamwa Ngassam RI, Sumo L, et al. Mapping of schistosomiasis and soil-transmitted helminthiasis in the regions of centre, east and west Cameroon. *PLoS Neglected Tropical Diseases.* 2012;6(3):e1553.

367. Adoubryn K, Kouadio-Yapo C, Ouhon J, Aka N, Bintto F, Assoumou A. Parasitoses intestinales infantiles à Biankouma, région des 18 Montagnes (ouest de la Côte d’Ivoire): étude de l’efficacité et de la tolérance du praziquantel et de l’albendazole. *Médecine et Santé Tropicales.* 2012;22(2):170-176.

368. Edelduok EG, Eke FN, Evelyn NE, Atama CI, Eyo JE. Efficacy of a single dose albendazole chemotherapy on human intestinal helminthiasis among school children in selected rural tropical communities. *Annals of Tropical Medicine and Public Health.* 2013;6(4):413.

369. Emile N, Bosco NJ, Karine B. Prevalence of intestinal parasitic infections and associated risk factors among Kigali Institute of Education students in Kigali, Rwanda. *Tropical biomedicine.* 2013;30(4):718-726.

370. Fentie T, Erqou S, Gedefaw M, Desta A. Epidemiology of human fascioliasis and intestinal parasitosis among schoolchildren in Lake Tana Basin, northwest Ethiopia. *Transactions of the Royal Society of Tropical Medicine and Hygiene.* 2013;107(8):480-486.

371. Gelaw A, Anagaw B, Nigussie B, et al. Prevalence of intestinal parasitic infections and risk factors among schoolchildren at the University of Gondar Community School, Northwest Ethiopia: a cross-sectional study. *BMC Public Health.* 2013;13(1):1-7.

372. King JD, Endeshaw T, Escher E, et al. Intestinal parasite prevalence in an area of Ethiopia after implementing the SAFE strategy, enhanced outreach services, and health extension program. *PLoS neglected tropical diseases.* 2013;7(6):e2223.

373. Obala A, Simiyu C, Odhiambo D, et al. Webuye health and demographic surveillance systems baseline survey of soil-transmitted helminths and intestinal protozoa among children up to five years. *Journal of tropical medicine.* 2013;2013.

374. Wegayehu T, Tsalla T, Seifu B, Teklu T. Prevalence of intestinal parasitic infections among highland and lowland dwellers in Gamo area, South Ethiopia. *BMC Public Health.* 2013;13(1):1-7.

375. Abanyie FA, McCracken C, Kirwan P, et al. Ascaris co-infection does not alter malaria-induced anaemia in a cohort of Nigerian preschool children. *Malaria Journal.* 2013;12(1):1-8.

376. Alio HM, Tidjani A, Otchom BB, Tidjani MT, Felix BBC. An epidemiological assessment of the infectious forms of intestinal helminths in school children from Chad. *Journal of Biology and Life Sciences.* 2013;4(2):341.

377. Amare B, Ali J, Moges B, et al. Nutritional status, intestinal parasite infection and allergy among school children in Northwest Ethiopia. *BMC pediatrics.* 2013;13(1):1-9.

378. Amollo D, Kihara J, Kombe Y, Karanja S. Prevalence and intensity of single and mixed schistosoma mansoni and schistosoma haematobium infections in primary school children in Rachuonyo North District, Homabay County, Western Kenya. *East African medical journal.* 2013;90(2):36-44.

379. Bustinduy AL, Parraga IM, Thomas CL, et al. Impact of polyparasitic infections on anemia and undernutrition among Kenyan children living in a Schistosoma haematobium-endemic area. *The American journal of tropical medicine and hygiene.* 2013;88(3):433.

380. Fürst T, Ouattara M, Silué KD, et al. Scope and Limits of an Anamnestic Questionnaire in a Control-Induced Low-Endemicity Helminthiasis Setting in South-Central Côte d’Ivoire. *PLoS One.* 2013;8(6):e64380.

381. Katungi A, Redeker S, Kiiza P, et al. Reassessment of Helminth Infections in Gulu Municipality Nothern Uganda after the Twenty Years of Insurgency: Using Three Diagonostic Methods to Compare their Sensitivity. *East African medical journal.* 2013;90(3):95-103.

382. Mahmud MA, Spigt M, Mulugeta Bezabih A, López Pavon I, Dinant GJ, Blanco Velasco R. Risk factors for intestinal parasitosis, anaemia, and malnutrition among school children in Ethiopia. *Pathog Glob Health.* 2013;107(2):58-65.

383. Mwandawiro CS, Nikolay B, Kihara JH, et al. Monitoring and evaluating the impact of national school-based deworming in Kenya: study design and baseline results. *Parasites & vectors.* 2013;6(1):1-14.

384. Schmidlin T, Hürlimann E, Silué KD, et al. Effects of hygiene and defecation behavior on helminths and intestinal protozoa infections in Taabo, Côte d’Ivoire. *PLoS One.* 2013;8(6):e65722.

385. Tchuem Tchuenté L-A, Dongmo Noumedem C, Ngassam P, et al. Mapping of schistosomiasis and soil-transmitted helminthiasis in the regions of Littoral, North-West, South and South-West Cameroon and recommendations for treatment. *BMC Infectious Diseases.* 2013;13(1):1-12.

386. Tekeste Z, Belyhun Y, Gebrehiwot A, et al. Epidemiology of intestinal schistosomiasis and soil transmitted helminthiasis among primary school children in Gorgora, Northwest Ethiopia. *Asian Pacific Journal of Tropical Disease.* 2013;3(1):61-64.

387. Abera A, Nibret E. Prevalence of gastrointestinal helminthic infections and associated risk factors among schoolchildren in Tilili town, northwest Ethiopia. *Asian Pacific journal of tropical medicine.* 2014;7(7):525-530.

388. Akanni EO, Adefioye OA, Akanni RA, Taiwo SS. Iron deficiency anaemia associated with helminths and asymptomatic malaria infections among rural school children in Southwestern Nigeria. *Asian Pacific Journal of Tropical Disease.* 2014;4:S590-S594.

389. Andereck JW, Kipp AM, Ondiek M, Vermund SH. Helminth prevalence among adults in rural Kenya: a stool survey for soil-transmitted helminths and schistosomiasis in Nyanza province. *Transactions of the Royal Society of Tropical Medicine and Hygiene.* 2014;108(12):804-809.

390. Jejaw A, Zeynudin A, Zemene E, Belay T. Status of intestinal parasitic infections among residents of Jimma Town, Ethiopia. *BMC research notes.* 2014;7(1):1-6.

391. Lobo ML, Augusto J, Antunes F, et al. Cryptosporidium spp., Giardia duodenalis, Enterocytozoon bieneusi and other intestinal parasites in young children in Lobata province, Democratic Republic of São Tomé and Principe. *PLoS One.* 2014;9(5):e97708.

392. Tulu B, Taye S, Amsalu E. Prevalence and its associated risk factors of intestinal parasitic infections among Yadot primary school children of South Eastern Ethiopia: a cross-sectional study. *BMC research notes.* 2014;7(1):1-7.

393. Yihenew G, Adamu H, Petros B. The impact of cooperative social organization on reducing the prevalence of malaria and intestinal parasite infections in Awramba, a rural community in South Gondar, Ethiopia. *Interdisciplinary perspectives on infectious diseases.* 2014;2014.

394. Bird C, Ame S, Albonico M, Bickle Q. Do shoes reduce hookworm infection in school-aged children on Pemba Island, Zanzibar? A pragmatic trial. *Transactions of The Royal Society of Tropical Medicine and Hygiene.* 2014;108(5):297-304.

395. G/hiwot Y, Degarege A, Erko B. Prevalence of intestinal parasitic infections among children under five years of age with emphasis on Schistosoma mansoni in Wonji Shoa Sugar Estate, Ethiopia. *PLoS One.* 2014;9(10):e109793.

396. Hürlimann E, Houngbedji CA, Yapi RB, et al. Health-related quality of life among school children with parasitic infections: findings from a national cross-sectional survey in Côte d'Ivoire. *PLoS neglected tropical diseases.* 2014;8(12):e3287.

397. Ibikounlé M, Gbédjissi L, Ogouyèmi-Hounto A, Batcho W, Kindé-Gazard D, Massougbodji A. Schistosomose et géohelminthoses dans le nord-est du Bénin: cas des écoliers des communes de Nikki et de Pèrèrè. *Bulletin de la Société de pathologie exotique.* 2014;107(3):171-176.

398. Matangila JR, Doua JY, Linsuke S, et al. Malaria, schistosomiasis and soil transmitted helminth burden and their correlation with anemia in children attending primary schools in Kinshasa, Democratic Republic of Congo. *PLoS One.* 2014;9(11):e110789.

399. Mathewos B, Alemu A, Woldeyohannes D, et al. Current status of soil transmitted helminths and Schistosoma mansoni infection among children in two primary schools in North Gondar, Northwest Ethiopia: a cross sectional study. *BMC Research Notes.* 2014;7(1):1-7.

400. Midzi N, Mduluza T, Chimbari MJ, et al. Distribution of schistosomiasis and soil transmitted helminthiasis in Zimbabwe: towards a national plan of action for control and elimination. *PLoS neglected tropical diseases.* 2014;8(8):e3014.

401. Mwakitalu ME, Malecela MN, Mosha FW, Simonsen PE. Urban schistosomiasis and soil transmitted helminthiases in young school children in Dar es Salaam and Tanga, Tanzania, after a decade of anthelminthic intervention. *Acta tropica.* 2014;133:35-41.

402. Nundu Sabiti S, Aloni MN, Linsuke SW, et al. [Prevalence of geohelminth infections in children living in Kinshasa]. *Arch Pediatr.* 2014;21(6):579-583.

403. Schüle SA, Clowes P, Kroidl I, et al. Ascaris lumbricoides infection and its relation to environmental factors in the Mbeya region of Tanzania, a cross-sectional, population-based study. *PLoS One.* 2014;9(3):e92032.

404. Abah A, Arene F. Status of intestinal parasitic infections among primary school children in Rivers State, Nigeria. *Journal of parasitology research.* 2015;2015.

405. Ajayi IO, Afonne C, Dada-Adegbola H, Falade CO. Prevalence of asymptomatic malaria and intestinal helminthiasis co-infection among children living in selected rural communities in Ibadan Nigeria. *American Journal of Epidemiology and Infectious Disease.* 2015;3(1):15-20.

406. Bugssa G, Dessalegn B, Alemu M, Desta H, Kahsay T. A survey of intestinal parasitic infections among Dega Ochollo primary school children Ochollo South Ethiopia. *Science Journal of Public Health.* 2015;3(1):56-60.

407. Chioma U, Mbanugo J, Nwachukwu E. Prevalence of intestinal helminthes parasite in stools of nursery and primary schools pupils in Uga, Anambra State, Nigeria. *Sky Journal of Microbiology Research.* 2015;3(1):006-010.

408. Dada EO. Prevalence of human intestinal helminth parasites among undergraduate students at the off campus (North gate area), Federal University of Technology, Akure (FUTA), Nigeria. *Open Access Library Journal.* 2015;2(03):1.

409. Dankwa K, Kumi RO, Ephraim RK, et al. Intestinal parasitosis among primary school pupils in coastal areas of the Cape Coast Metropolis, Ghana. *Children.* 2015;4:5.

410. Efunshile AM, Olawale T, Stensvold CR, Kurtzhals JA, König B. Epidemiological study of the association between malaria and helminth infections in Nigeria. *The American Journal of Tropical Medicine and Hygiene.* 2015;92(3):578.

411. Gebreslassie M, Dejenie T, Tomass Z. Prevalence of Intestinal Parasites and Associated Risk Factors in Schoolchildren of Aksum Town, Northern Ethiopia. *Acta Parasitologica Globalis.* 2015;6(1):42-48.

412. Gwetu TP, Chhagan M, Craib M, Taylor M, Kauchali S. Persistent and new-onset anaemia in children aged 6-8 years from KwaZulu-Natal Province, South Africa. *South African Journal of Child Health.* 2015;9(4):127-129.

413. Jejaw A, Zemene E, Alemu Y, Mengistie Z. High prevalence of Schistosoma mansoni and other intestinal parasites among elementary school children in Southwest Ethiopia: a cross-sectional study. *BMC Public Health.* 2015;15(1):1-7.

414. Meles W, Merid I, Asfaw M. A study of the incidence of intestinal helminthic diseases and their risk factors among school Children in Lumame town, Northwest, Ethiopia. *Afr J Parasitol Res.* 2015;2(10):152-160.

415. Meurs L, Polderman AM, Vinkeles Melchers NV, et al. Diagnosing polyparasitism in a high-prevalence setting in Beira, Mozambique: detection of intestinal parasites in fecal samples by microscopy and real-time PCR. *PLoS neglected tropical diseases.* 2017;11(1):e0005310.

416. Mohammed K, Abdullah MR, Omar J, Eugene II, Ismail A. Intestinal parasitic infection and assessment of risk factors in North-Western, Nigeria: A community based study. *International Journal of Pharma Medicine and Biological Sciences.* 2015;4(2):141.

417. Mwale K, Siziya S. Intestinal infestations in under-five children in Zambia. *International Journal of MCH and AIDS.* 2015;4(2):40.

418. Njunda AL, Fon SG, Assob JCN, Nsagha DS, Kwenti TDB, Kwenti TE. Coinfection with malaria and intestinal parasites, and its association with anaemia in children in Cameroon. *Infectious diseases of poverty.* 2015;4(1):1-7.

419. Nxasana N, Baba K, Bhat V, Vasaikar S. Prevalence of intestinal parasites in primary school children of Mthatha, Eastern Cape Province, South Africa. *Annals of medical and health sciences Research.* 2013;3(4):511-516.

420. Odugbemi T, Akinrujomu V, Onajole A. Prevalence of intestinal helminth infections among primary school children in Alimosho Local Government Area, Lagos, Nigeria. *Journal of Community Medicine and Primary Health Care.* 2015;27(1):64-78.

421. Oliveira D, Ferreira FS, Atouguia J, Fortes F, Guerra A, Centeno-Lima S. Infection by intestinal parasites, stunting and anemia in school-aged children from southern Angola. *PLoS One.* 2015;10(9):e0137327.

422. Ugochi UJ, Ifenyinwa M, Ijeoma E-N, Godson UM, Nwaku AI. Prevalence of intestinal parasites among primary school children in three geopolitical zones of imo state, Nigeria. *Science Journal of public health.* 2015;3(5-1):25-28.

423. Yahaya A, Tyav YB, Idris A. Prevalence of intestinal parasitic helminths from fingernails of “almajiris” in Birnin Kudu local government area, Jigawa state, Nigeria. *Int J Trop Dis Health.* 2015;8:66-74.

424. Alelign T, Degarege A, Erko B. Soil-transmitted helminth infections and associated risk factors among schoolchildren in Durbete town, northwestern Ethiopia. *Journal of parasitology research.* 2015;2015.

425. Alemayehu B, Tomass Z. Schistosoma mansoni infection prevalence and associated risk factors among schoolchildren in Demba Girara, Damot Woide District of Wolaita Zone, Southern Ethiopia. *Asian Pacific journal of tropical medicine.* 2015;8(6):457-463.

426. Drabo F, Ouedraogo H, Bougma R, et al. Successful control of soil-transmitted helminthiasis in school age children in Burkina Faso and an example of community-based assessment via lymphatic filariasis transmission assessment survey. *PLoS neglected tropical diseases.* 2016;10(5):e0004707.

427. Emana D, Jemal K, Bajiro M, Mekonnen Z. Prevalence and intensity of soil-transmitted helminths among school-aged children in Sigmo primary school, Jimma Zone, South-Western Ethiopia. *Clinical Medicine Research.* 2015;4(4):98-103.

428. Freeman M, Chard A, Nikolay B, et al. Associations between school-and household-level water, sanitation and hygiene conditions and soil-transmitted helminth infection among Kenyan school children. *Parasites & vectors.* 2015;8(1):1-13.

429. Gashaw F, Aemero M, Legesse M, et al. Prevalence of intestinal helminth infection among school children in Maksegnit and Enfranz Towns, northwestern Ethiopia, with emphasis on Schistosoma mansoni infection. *Parasites & vectors.* 2015;8(1):1-8.

430. Kepha S, Nuwaha F, Nikolay B, et al. Epidemiology of coinfection with soil transmitted helminths and Plasmodium falciparum among school children in Bumula District in western Kenya. *Parasites & vectors.* 2015;8(1):1-10.

431. Kuete T, Yemeli FLS, Mvoa EE, Nkoa T, Somo RM, Ekobo AS. Prevalence and risk factors of intestinal helminth and protozoa infections in an urban setting of Cameroon: the case of Douala. *Am J Epidemiol Infect Dis.* 2015;3(2):36-44.

432. Kure A, Mekonnen Z, Dana D, et al. Comparison of individual and pooled stool samples for the assessment of intensity of Schistosoma mansoni and soil-transmitted helminth infections using the Kato-Katz technique. *Parasites & vectors.* 2015;8(1):1-9.

433. Nikolay B, Mwandawiro CS, Kihara JH, et al. Understanding heterogeneity in the impact of national neglected tropical disease control programmes: evidence from school-based deworming in Kenya. *PLoS neglected tropical diseases.* 2015;9(9):e0004108.

434. Nwalorzie C, Onyenakazi S, Ogwu S, Okafor A. Predictors of intestinal helminthic infections among school children in Gwagwalada, Abuja, Nigeria. *Nigerian Journal of Medicine: Journal of the National Association of Resident Doctors of Nigeria.* 2015;24(3):233-241.

435. Salawu S, Ughele V. Prevalence of soil-transmitted helminths among school-age children in Ife East Local Government Area, Osun State, Nigeria. *FUTA Journal of Research in Sciences.* 2015;11(1):139-151.

436. Salim N, Knopp S, Lweno O, et al. Distribution and risk factors for Plasmodium and helminth co-infections: a cross-sectional survey among children in Bagamoyo district, coastal region of Tanzania. *PLoS neglected tropical diseases.* 2015;9(4):e0003660.

437. Seid M, Dejenie T, Tomass Z. Prevalence of intestinal helminths and associated risk factors in rural schoolchildren in Were-Abaye sub-district, Tigray region, northern Ethiopia. *Acta Parasitologica Globalis.* 2015;6(1):29.

438. Siwila J, Olsen A. Risk factors for infection with soil transmitted helminths, Cryptosporidium spp., and Giardia duodenalis in children enrolled in preschools in Kafue District, Zambia. *Epidemiology Research International.* 2015;2015.

439. Siza JE, Kaatano GM, Chai J-Y, et al. Prevalence of schistosomes and soil-transmitted helminths and morbidity associated with schistosomiasis among adult population in Lake Victoria Basin, Tanzania. *The Korean journal of parasitology.* 2015;53(5):525.

440. Siza JE, Kaatano GM, Chai J-Y, et al. Prevalence of schistosomes and soil-transmitted helminths among schoolchildren in Lake Victoria Basin, Tanzania. *The Korean Journal of Parasitology.* 2015;53(5):515.

441. Shumbej T, Belay T, Mekonnen Z, Tefera T, Zemene E. Soil-transmitted helminths and associated factors among pre-school children in Butajira Town, South-Central Ethiopia: a community-based cross-sectional study. *PLoS One.* 2015;10(8):e0136342.

442. Frickmann H, Schwarz NG, Rakotozandrindrainy R, May J, Hagen RM. PCR for enteric pathogens in high-prevalence settings. What does a positive signal tell us? *Infectious Diseases.* 2015;47(7):491-498.

443. Odinaka KK, Nwolisa EC, Mbanefo F, Iheakaram AC, Okolo S. Prevalence and pattern of soil-transmitted helminthic infection among primary school children in a rural community in Imo State, Nigeria. *Journal of Tropical Medicine.* 2015;2015.

444. Adelakun AA, Arinola OG. Prevalence of food sensitization and helminth infection among primary school children in Ibadan, southwest Nigeria. *Egyptian Journal of Pediatric Allergy and Immunology (the).* 2016;14(1):23-29.

445. Amor A, Rodriguez E, Saugar JM, et al. High prevalence of Strongyloides stercoralis in school-aged children in a rural highland of north-western Ethiopia: the role of intensive diagnostic work-up. *Parasites & vectors.* 2016;9(1):1-8.

446. Aniwada EC, Uleanya ND, Igbokwe LN, Onwasigwe C. Soil transmitted helminths; prevalence, perception and determinants among primary school children in rural Enugu State, Nigeria. *International Journal of Tropical Disease and Health.* 2016;15(1):1-12.

447. Bayoumi M, Nykwac O, Kardaman M, et al. Intestinal parasitic infections in school students in Malakal city, Upper Nile State, South Sudan. *SOJ Microbiol Inf Dis.* 2016;4(1):1-5.

448. Afework Bitew A, Abera B, Seyoum W, et al. Soil-transmitted helminths and Schistosoma mansoni infections in Ethiopian Orthodox Church students around Lake Tana, northwest Ethiopia. *PLoS One.* 2016;11(5):e0155915.

449. Dada E. PREVALENCE OF HUMAN INTESTINAL HELMINTH PARASITES AMONG PRYMARY SCHOOL CHILDREN IN IPOGUN, IFEDORE LOCAL GOVERNMENT AREA, NIGERIA. *Journal of Global Biosciences.* 2016;5(1):3401-3407.

450. M’bondoukwé N, Mawili-Mboumba D, Mondouo Manga F, Kombila M, Bouyou-Akotet M. Prevalence of soil-transmitted Helminths and intestinal Protozoa in shanty towns of Libreville, Gabon. *Int J Trop Dis Heal.* 2016;20:1-9.

451. Mekonnen Z, Suleman S, Biruksew A, Tefera T, Chelkeba L. Intestinal polyparasitism with special emphasis to soil-transmitted helminths among residents around Gilgel Gibe Dam, Southwest Ethiopia: a community based survey. *BMC Public Health.* 2016;16(1):1-7.

452. Tyoalumun K, Abubakar S, Christopher N. Prevalence of intestinal parasitic infections and their association with nutritional status of rural and urban pre-school children in Benue State, Nigeria. *International Journal of MCH and AIDS.* 2016;5(2):146.

453. Alemu A, Tegegne Y, Damte D, Melku M. Schistosoma mansoni and soil-transmitted helminths among preschool-aged children in Chuahit, Dembia district, Northwest Ethiopia: prevalence, intensity of infection and associated risk factors. *BMC Public Health.* 2016;16(1):1-9.

454. Assaré RK, Tian-Bi Y-NT, Yao PK, et al. Sustaining control of schistosomiasis mansoni in western Côte d’Ivoire: results from a SCORE study, one year after initial praziquantel administration. *PLoS neglected tropical diseases.* 2016;10(1):e0004329.

455. Boko PM, Ibikounle M, Onzo-Aboki A, et al. Schistosomiasis and soil transmitted helminths distribution in Benin: a baseline prevalence survey in 30 districts. *PLoS One.* 2016;11(9):e0162798.

456. Bopda J, Nana-Djeunga H, Tenaguem J, et al. Prevalence and intensity of human soil transmitted helminth infections in the Akonolinga health district (Centre Region, Cameroon): Are adult hosts contributing in the persistence of the transmission? *Parasite epidemiology and control.* 2016;1(2):199-204.

457. Easton AV, Oliveira RG, O’Connell EM, et al. Multi-parallel qPCR provides increased sensitivity and diagnostic breadth for gastrointestinal parasites of humans: field-based inferences on the impact of mass deworming. *Parasites & vectors.* 2016;9(1):1-12.

458. Erismann S, Diagbouga S, Odermatt P, et al. Prevalence of intestinal parasitic infections and associated risk factors among schoolchildren in the Plateau Central and Centre-Ouest regions of Burkina Faso. *Parasites & vectors.* 2016;9(1):1-14.

459. Ferreira FS, Baptista-Fernandes T, Oliveira D, et al. Giardia duodenalis and soil-transmitted helminths infections in children in São Tomé and Príncipe: do we think Giardia when addressing parasite control? *Journal of tropical pediatrics.* 2015;61(2):106-112.

460. Fuhrimann S, Winkler MS, Kabatereine NB, et al. Risk of intestinal parasitic infections in people with different exposures to wastewater and fecal sludge in Kampala, Uganda: a cross-sectional study. *PLoS neglected tropical diseases.* 2016;10(3):e0004469.

461. Garn JV, Mwandawiro CS, Nikolay B, et al. Ascaris lumbricoides infection following school-based deworming in western Kenya: assessing the role of pupils' school and home water, sanitation, and hygiene exposures. *The American journal of tropical medicine and hygiene.* 2016;94(5):1045.

462. Grimes JE, Tadesse G, Mekete K, et al. School water, sanitation, and hygiene, soil-transmitted helminths, and schistosomes: national mapping in Ethiopia. *PLoS neglected tropical diseases.* 2016;10(3):e0004515.

463. Knoblauch AM, Archer C, Owuor M, et al. Schistosomiasis and soil-transmitted helminth infections in schoolchildren in north-eastern Democratic Republic of the Congo. *Transactions of The Royal Society of Tropical Medicine and Hygiene.* 2016;110(7):424-426.

464. Mirante C, Clemente I, Zambu G, et al. Comparing concentration methods: parasitrap® versus Kato-Katz for studying the prevalence of Helminths in Bengo province, Angola. *African health sciences.* 2016;16(3):698-703.

465. Müller I, Yap P, Steinmann P, et al. Intestinal parasites, growth and physical fitness of schoolchildren in poor neighbourhoods of Port Elizabeth, South Africa: a cross-sectional survey. *Parasites & Vectors.* 2016;9(1):1-13.

466. Ortu G, Assoum M, Wittmann U, et al. The impact of an 8-year mass drug administration programme on prevalence, intensity and co-infections of soil-transmitted helminthiases in Burundi. *Parasites & vectors.* 2016;9(1):1-17.

467. Rasoamanamihaja CF, Rahetilahy AM, Ranjatoarivony B, et al. Baseline prevalence and intensity of schistosomiasis at sentinel sites in Madagascar: informing a national control strategy. *Parasites & vectors.* 2016;9(1):1-10.

468. Webb E, Nampijja M, Kaweesa J, et al. Helminths are positively associated with atopy and wheeze in Ugandan fishing communities: results from a cross‐sectional survey. *Allergy.* 2016;71(8):1156-1169.

469. Worrell CM, Wiegand RE, Davis SM, et al. A cross-sectional study of water, sanitation, and hygiene-related risk factors for soil-transmitted helminth infection in urban school-and preschool-aged children in Kibera, Nairobi. *PLoS One.* 2016;11(3):e0150744.

470. Yapi RB, Chammartin F, Hürlimann E, et al. Bayesian risk profiling of soil-transmitted helminth infections and estimates of preventive chemotherapy for school-aged children in Côte d'Ivoire. *Parasites & vectors.* 2016;9(1):1-9.

471. Abdi M, Nibret E, Munshea A. Prevalence of intestinal helminthic infections and malnutrition among schoolchildren of the Zegie Peninsula, northwestern Ethiopia. *Journal of infection and public health.* 2017;10(1):84-92.

472. Adeniran AA, Mogaji HO, Aladesida AA, et al. Schistosomiasis, intestinal helminthiasis and nutritional status among preschool-aged children in sub-urban communities of Abeokuta, Southwest, Nigeria. *BMC research notes.* 2017;10(1):1-7.

473. Aiemjoy K, Gebresillasie S, Stoller NE, et al. Epidemiology of soil-transmitted helminth and intestinal protozoan infections in preschool-aged children in the Amhara region of Ethiopia. *The American journal of tropical medicine and hygiene.* 2017;96(4):866.

474. de Alegría MLAR, Colmenares K, Espasa M, et al. Prevalence of Strongyloides stercoralis and other intestinal parasite infections in school children in a rural area of Angola: a cross-sectional study. *The American Journal of Tropical Medicine and Hygiene.* 2017;97(4):1226.

475. Haile A, Abera T, Dana D, Wolkite E. The prevalence of intestinal parasitic infection and associated factors among primary school children in Gurage Zone, South Ethiopia. *Prevalence.* 2017;15.

476. Manir N, Umar L, Abduhadi B. Survey on prevalence of intestinal parasites associated with some primary school aged children in Dutsinma Area, Katsina State, Nigeria. *MOJ Biol Med.* 2017;2(2):00044.

477. Mirisho R, Neizer ML, Sarfo B. Prevalence of intestinal helminths infestation in children attending Princess Marie Louise Children’s Hospital in Accra, Ghana. *Journal of parasitology research.* 2017;2017.

478. Onyido A, JU A, Ezechukwu G, Ugha C, Umeanaeto P, Iwueze M. Intestinal helminth infections among primary school pupils in Ekwulumili community, Nnewi South local government area, Anambra state. *Nigerian Journal of Parasitology.* 2017;38(2):185-192.

479. Oswald WE, Stewart AE, Kramer MR, et al. Association of community sanitation usage with soil-transmitted helminth infections among school-aged children in Amhara Region, Ethiopia. *Parasites & vectors.* 2017;10(1):1-13.

480. Siddig HS, Mohammed IA, Mohammed MN, Bashir AM. Prevalence of intestinal parasites among selected group of primary school children in Alhag Yousif Area, Khartoum, Sudan. *International Journal of Medical Research & Health Sciences.* 2017;6(8):125-131.

481. Shitta K, Akogun O. Intestinal helminth infections among the nomadic Fulanis in two localities of Adamawa State, North-East Nigeria. *Nigerian Journal of Parasitology.* 2017;38(1):69-73.

482. Tadege B, Shimelis T. Infections with Schistosoma mansoni and geohelminths among school children dwelling along the shore of the Lake Hawassa, southern Ethiopia. *PLoS One.* 2017;12(7):e0181547.

483. Adriko M, Tinkitina B, Arinaitwe M, Kabatereine NB, Nanyunja M, M. Tukahebwa E. Impact of a national deworming campaign on the prevalence of soil-transmitted helminthiasis in Uganda (2004-2016): implications for national control programs. *PLOS Neglected Tropical Diseases.* 2018;12(7):e0006520.

484. Akinwande K, Morenikeji O, Arinola OG. Anthropometric indices and serum micronutrient status of helminth–infected school children from semi-urban communities in Southwestern Nigeria. *Nigerian Journal of Physiological Sciences.* 2017;32(2):195-200.

485. Alemayehu B, Tomass Z, Wadilo F, Leja D, Liang S, Erko B. Epidemiology of intestinal helminthiasis among school children with emphasis on Schistosoma mansoni infection in Wolaita zone, Southern Ethiopia. *BMC Public Health.* 2017;17(1):1-10.

486. Campbell SJ, Stothard JR, O’Halloran F, et al. Urogenital schistosomiasis and soil-transmitted helminthiasis (STH) in Cameroon: an epidemiological update at Barombi Mbo and Barombi Kotto crater lakes assessing prospects for intensified control interventions. *Infectious diseases of poverty.* 2017;6(01):36-48.

487. Inocencio da Luz R, Linsuke S, Lutumba P, Hasker E, Boelaert M. Assessment of schistosomiasis and soil‐transmitted helminths prevalence in school‐aged children and opportunities for integration of control in local health services in Kwilu Province, the Democratic Republic of the Congo. *Trop Med Int Health.* 2017;22(11):1442-1450.

488. Grimes JE, Tadesse G, Gardiner IA, et al. Sanitation, hookworm, anemia, stunting, and wasting in primary school children in southern Ethiopia: Baseline results from a study in 30 schools. *PLoS neglected tropical diseases.* 2017;11(10):e0005948.

489. Halwindi H, Magnussen P, Olsen A, Lisulo M. Potential contribution of adult populations to the maintenance of schistosomiasis and soil-transmitted helminth infections in the Siavonga and Mazabuka districts of Zambia. *Journal of biosocial science.* 2017;49(2):265-275.

490. Ito E, Egwunyenga A. Soil-transmitted helminthiasis in Aviara Community: an observation from primary school children in Nigeria. *International Medical Journal.* 2017;24(2):205-208.

491. Kabore A, Ibikounle M, Tougoue JJ, et al. Initiating NTD programs targeting schistosomiasis and soil-transmitted helminthiasis in two provinces of the Democratic Republic of the Congo: Establishment of baseline prevalence for mass drug administration. *Acta tropica.* 2017;166:177-185.

492. Moser W, Labhardt ND, Cheleboi M, Muhairwe J, Keiser J. Unexpected low soil-transmitted helminth prevalence in the Butha-Buthe district in Lesotho, results from a cross-sectional survey. *Parasites & Vectors.* 2017;10(1):1-5.

493. Munisi DZ, Buza J, Mpolya EA, Kinung’hi SM. Schistosoma mansoni infections, undernutrition and anaemia among primary schoolchildren in two onshore villages in Rorya District, North-Western Tanzania. *PLoS One.* 2016;11(12):e0167122.

494. Noche CD, Kwetche PRF, Tumameu T, Tambo E, Moyou R, Bella AL. Relationship between tropical endemic limbo-conjunctivitis and intestinal helminths in a population of Cameroonian children. *Cogent Medicine.* 2017;4(1).

495. Sakari SSW, Mbugua AK, Mkoji GM. Prevalence of soil-transmitted helminthiases and schistosomiasis in preschool age children in Mwea division, Kirinyaga south district, Kirinyaga county, and their potential effect on physical growth. *Journal of Tropical Medicine.* 2017;2017.

496. Shittu O, Shittu DS, Opeyemi OA, et al. Overlapping distribution of Plasmodium falciparum and soil transmitted helminths in a malaria hyper-endemic region, North-Central Nigeria. *Asian Paci J Tropi Dis.* 2017;7(12):930-935.

497. Sumbele IUN, Nkemnji GB, Kimbi HK. Soil-transmitted helminths and plasmodium falciparum malaria among individuals living in different agroecosystems in two rural communities in the mount Cameroon area: a cross-sectional study. *Infectious diseases of poverty.* 2017;6(1):1-15.

498. Tefera E, Belay T, Mekonnen SK, Zeynudin A, Belachew T. Prevalence and intensity of soil transmitted helminths among school children of Mendera Elementary School, Jimma, Southwest Ethiopia. *The Pan African Medical Journal.* 2017;27.

499. Diongue K, Ndiaye M, Seck MC, Ndiaye YD, Badiane AS, Ndiaye D. Distribution of parasites detected in stool samples of patients in Le Dantec University Hospital of Dakar, Senegal, from 2011 to 2015. *Journal of tropical medicine.* 2017;2017.

500. Adu-Gyasi D, Asante KP, Frempong MT, et al. Epidemiology of soil transmitted Helminth infections in the middle-belt of Ghana, Africa. *Parasite epidemiology and control.* 2018;3(3):e00071.

501. Akwa Y, Jafaaru A, Elkanah O, et al. Prevalence of gastrointestinal helminths infections among school-aged children in Kurmi Local Government Area, Taraba State, Nigeria. *Nigerian Journal of Parasitology.* 2018;39(2):167-172.

502. Alemu G, Aschalew Z, Zerihun E. Burden of intestinal helminths and associated factors three years after initiation of mass drug administration in Arbaminch Zuria district, southern Ethiopia. *BMC infectious diseases.* 2018;18(1):1-8.

503. Forson AO, Arthur I, Ayeh-Kumi PF. The role of family size, employment and education of parents in the prevalence of intestinal parasitic infections in school children in Accra. *PLoS One.* 2018;13(2):e0192303.

504. Hailegebriel T. Undernutrition, intestinal parasitic infection and associated risk factors among selected primary school children in Bahir Dar, Ethiopia. *BMC infectious diseases.* 2018;18(1):1-11.

505. Kiki-Barro P, Kassi F, Vanga-Bosson H, et al. Low prevalence of intestinal helminth infections among primary school children in Tengrela, northern Cô te d’Ivoire. *Nigerian Journal of Parasitology.* 2018;39(1):67-73.

506. M’bondoukwé NP, Kendjo E, Mawili-Mboumba DP, et al. Prevalence of and risk factors for malaria, filariasis, and intestinal parasites as single infections or co-infections in different settlements of Gabon, Central Africa. *Infectious diseases of poverty.* 2018;7(1):1-17.

507. Nute AW, Endeshaw T, Stewart AE, et al. Prevalence of soil-transmitted helminths and Schistosoma mansoni among a population-based sample of school-age children in Amhara region, Ethiopia. *Parasites & vectors.* 2018;11(1):1-9.

508. Ojja S, Kisaka S, Ediau M, et al. Prevalence, intensity and factors associated with soil-transmitted helminths infections among preschool-age children in Hoima district, rural western Uganda. *BMC infectious diseases.* 2018;18(1):1-12.

509. Okike-Osisiogu F, Nwoke B, Ukaga C, Amaechi A, Ezeigbo O, Ike-Amadi C. Prevalence of intestinal parasites and bacteria among school pupils in Aba, Abia State. *Nigerian Journal of Parasitology.* 2018;39(1):74-78.

510. Olopade BO, Idowu CO, Oyelese AO, Aboderin AO. Intestinal parasites, nutritional status and cognitive function among primary school pupils in Ile-Ife, Osun State, Nigeria. *African Journal of Infectious Diseases.* 2018;12(2):21-28.

511. Teklemariam D, Legesse M, Degarege A, Liang S, Erko B. Schistosoma mansoni and other intestinal parasitic infections in schoolchildren and vervet monkeys in Lake Ziway area, Ethiopia. *BMC research notes.* 2018;11(1):1-6.

512. Tékpa G, Fikouma V, Gbangba-Ngaï E, Mejiozem BB, Nazita SN, Koffi B. Epidemiological and clinical profile of intestinal parasitosis of children in rural areas in Central African Republic. *Archives de Pédiatrie.* 2019;26(1):34-37.

513. Tine RC, Sylla K, Sow D, et al. Low prevalence of soil transmitted helminths among children in rural areas in Senegal: A cross sectional survey. *Journal of Parasitology and Vector Biology.* 2018;10(1):19-25.

514. Unachukwu M, Nwakanma C. Prevalence of intestinal parasitic infection and malnutrition in Enugu Urban and Suburban Area. *International Journal of Medical Research and Review.* 2018;2(6):565-572.

515. Zemene T, Shiferaw MB. Prevalence of intestinal parasitic infections in children under the age of 5 years attending the Debre Birhan referral hospital, North Shoa, Ethiopia. *BMC research notes.* 2018;11(1):1-6.

516. Babamale OA, Ugbomoiko US, Heukelbach J. High prevalence of Plasmodium falciparum and soil-transmitted helminth co-infections in a periurban community in Kwara State, Nigeria. *Journal of infection and public health.* 2018;11(1):48-53.

517. Bronzan RN, Dorkenoo AM, Agbo YM, et al. Impact of community-based integrated mass drug administration on schistosomiasis and soil-transmitted helminth prevalence in Togo. *PLoS neglected tropical diseases.* 2018;12(8):e0006551.

518. Coulibaly G, Ouattara M, Dongo K, et al. Epidemiology of intestinal parasite infections in three departments of south-central Côte d’Ivoire before the implementation of a cluster-randomised trial. *Parasite Epidemiology and Control.* 2018;3(2):63-76.

519. Dejon-Agobé JC, Zinsou JF, Honkpehedji YJ, et al. Schistosoma haematobium effects on Plasmodium falciparum infection modified by soil-transmitted helminths in school-age children living in rural areas of Gabon. *PLoS neglected tropical diseases.* 2018;12(8):e0006663.

520. Fischer K, Gankpala A, Gankpala L, et al. Capillaria ova and diagnosis of Trichuris trichiura infection in humans by Kato-Katz smear, Liberia. *Emerging Infectious Diseases.* 2018;24(8):1551.

521. Hürlimann E, Silué KD, Zouzou F, et al. Effect of an integrated intervention package of preventive chemotherapy, community-led total sanitation and health education on the prevalence of helminth and intestinal protozoa infections in Côte d’Ivoire. *Parasites & vectors.* 2018;11(1):1-20.

522. Ibikounlé M, Onzo-Aboki A, Doritchamou J, et al. Results of the first mapping of soil-transmitted helminths in Benin: Evidence of countrywide hookworm predominance. *PLoS neglected tropical diseases.* 2018;12(3):e0006241.

523. Ibrahim T, Zemene E, Asres Y, et al. Epidemiology of soil-transmitted helminths and Schistosoma mansoni: a base-line survey among school children, Ejaji, Ethiopia. *The Journal of Infection in Developing Countries.* 2018;12(12):1134-1141.

524. Molla E, Mamo H. Soil-transmitted helminth infections, anemia and undernutrition among schoolchildren in Yirgacheffee, South Ethiopia. *BMC research notes.* 2018;11(1):1-7.

525. Leta GT, French M, Dorny P, Vercruysse J, Levecke B. Comparison of individual and pooled diagnostic examination strategies during the national mapping of soil-transmitted helminths and Schistosoma mansoni in Ethiopia. *PLoS neglected tropical diseases.* 2018;12(9):e0006723.

526. Teshale T, Belay S, Tadesse D, Awala A, Teklay G. Prevalence of intestinal helminths and associated factors among school children of Medebay Zana wereda; North Western Tigray, Ethiopia 2017. *BMC research notes.* 2018;11(1):1-6.

527. Tchakounté BN, Nkouayep VR, Poné JW. Soil contamination rate, prevalence, intensity of infection of geohelminths and associated risk factors among residents in Bazou (West Cameroon). *Ethiopian Journal of Health Sciences.* 2018;28(1):63-72.

528. Alemu G, Abossie A, Yohannes Z. Current status of intestinal parasitic infections and associated factors among primary school children in Birbir town, Southern Ethiopia. *BMC infectious diseases.* 2019;19(1):1-8.

529. Aribodor DN, Bassey SA, Yoonuan T, Sam-Wobo SO, Aribodor OB, Ugwuanyi IK. Analysis of Schistosomiasis and soil-transmitted helminths mixed infections among pupils in Enugu State, Nigeria: Implications for control. *Infection, Disease & Health.* 2019;24(2):98-106.

530. Dahal AS, Francis EO, Francis JE, Wamtas FI. Soil-transmitted helminths and associated risk factors among elementary school pupils in Dadin Kowa, Jos. *Nigerian Medical Journal: Journal of the Nigeria Medical Association.* 2019;60(4):181.

531. Geus D, Sifft KC, Habarugira F, et al. Co‐infections with Plasmodium, Ascaris and Giardia among Rwandan schoolchildren. *Trop Med Int Health.* 2019;24(4):409-420.

532. Gizaw Z, Addisu A, Dagne H. Effects of water, sanitation and hygiene (WASH) education on childhood intestinal parasitic infections in rural Dembiya, northwest Ethiopia: an uncontrolled before-and-after intervention study. *Environmental health and preventive medicine.* 2019;24(1):1-8.

533. Orish VN, Ofori-Amoah J, Amegan-Aho KH, et al. Prevalence of polyparasitic infection among primary school children in the Volta Region of Ghana. Paper presented at: Open Forum Infectious Diseases2019.

534. Sitotaw B, Mekuriaw H, Damtie D. Prevalence of intestinal parasitic infections and associated risk factors among Jawi primary school children, Jawi town, north-west Ethiopia. *BMC infectious diseases.* 2019;19(1):1-10.

535. Tekalign E, Bajiro M, Ayana M, Tiruneh A, Belay T. Prevalence and intensity of soil-transmitted helminth infection among rural community of southwest Ethiopia: a community-based study. *BioMed Research International.* 2019;2019.

536. Tongjura J, Ombugadu J, Abdullahi M, Blessing M, Amuga G, Mafuyai H. Intestinal parasites amongst primary school children attending Ta’al Model Primary School in Lafia Local Government Area of Nasarawa State, Nigeria. *Nigerian Journal of Parasitology.* 2019;40(1):92-96.

537. von Huth S, Kofoed P-E, Holmskov U. Prevalence and potential risk factors for gastrointestinal parasitic infections in children in urban Bissau, Guinea-Bissau. *Transactions of The Royal Society of Tropical Medicine and Hygiene.* 2019;113(9):545-554.

538. Bah YM, Bah MS, Paye J, et al. Soil-transmitted helminth infection in school age children in Sierra Leone after a decade of preventive chemotherapy interventions. *Infectious diseases of poverty.* 2019;8(04):31-40.

539. Bekana T, Hu W, Liang S, Erko B. Transmission of Schistosoma mansoni in Yachi areas, southwestern Ethiopia: new foci. *Infectious diseases of poverty.* 2019;8(01):18-25.

540. Gichuki PM, Mbugua G, Kiplelgo EK, Irungu TW, Mwandawiro C. Long Term School Based Deworming against Soil-Transmitted Helminths Also Benefits the Untreated Adult Population: Results from a Community-Wide Cross Sectional Survey. *Journal of Tropical Medicine.* 2019;2019.

541. Gyang VP, Chuang T-W, Liao C-W, et al. Intestinal parasitic infections: current status and associated risk factors among school aged children in an archetypal African urban slum in Nigeria. *Journal of Microbiology, Immunology and Infection.* 2019;52(1):106-113.

542. Hakami L, Castle PM, Kiernan J, et al. Epidemiology of soil transmitted helminth and Strongyloides stercoralis infections in remote rural villages of Ranomafana National Park, Madagascar. *Pathogens and global health.* 2019;113(2):94-100.

543. Halliday KE, Oswald WE, Mcharo C, et al. Community-level epidemiology of soil-transmitted helminths in the context of school-based deworming: Baseline results of a cluster randomised trial on the coast of Kenya. *PLoS neglected tropical diseases.* 2019;13(8):e0007427.

544. Ihejirika OC, Nwaorgu OC, Ebirim CI, Nwokeji CM. Effects of intestinal parasitic infections on nutritional status of primary children in Imo State Nigeria. *The Pan African Medical Journal.* 2019;33.

545. Loukouri A, Méité A, Kouadio OK, et al. Prevalence, intensity of soil-transmitted helminths, and factors associated with infection: importance in control program with ivermectin and albendazole in eastern Côte d’Ivoire. *Journal of Tropical Medicine.* 2019;2019.

546. Mekonnen HS, Ekubagewargies DT. Prevalence and factors associated with intestinal parasites among under-five children attending Woreta Health Center, Northwest Ethiopia. *BMC infectious diseases.* 2019;19(1):1-8.

547. Nkengni SMM, Zoumabo ATC, Soppa NPS, et al. Current decline in schistosome and soil-transmitted helminth infections among school children at Loum, Littoral region, Cameroon. *The Pan African Medical Journal.* 2019;33.

548. Osman KA, Zinsstag J, Tschopp R, et al. Nutritional status and intestinal parasites among young children from pastoralist communities of the Ethiopian Somali region. *Maternal & child nutrition.* 2020;16(3):e12955.

549. Sacolo-Gwebu H, Chimbari M, Kalinda C. Prevalence and risk factors of schistosomiasis and soil-transmitted helminthiases among preschool aged children (1–5 years) in rural KwaZulu-Natal, South Africa: a cross-sectional study. *Infectious diseases of poverty.* 2019;8(1):1-12.

550. Takeuchi R, Njenga SM, Ichinose Y, Kaneko S, Estrada CA, Kobayashi J. Is there a gap between health education content and practice toward schistosomiasis prevention among schoolchildren along the shores of Lake Victoria in Kenya? *PLoS neglected tropical diseases.* 2019;13(8):e0007572.

551. Tuasha N, Hailemeskel E, Erko B, Petros B. Comorbidity of intestinal helminthiases among malaria outpatients of Wondo Genet health centers, southern Ethiopia: implications for integrated control. *BMC infectious diseases.* 2019;19(1):1-8.

552. Weldesenbet H, Worku A, Shumbej T. Prevalence, infection intensity and associated factors of soil transmitted helminths among primary school children in Gurage zone, South Central Ethiopia: a cross-sectional study design. *BMC research notes.* 2019;12(1):1-6.

553. Addisu A, Zeleke AJ, Bayih AG, et al. Trends and seasonal patterns in intestinal parasites diagnosed in primary health facilities in Northwest Ethiopia. *The Journal of Infection in Developing Countries.* 2020;14(06.1):58S-65S.

554. Aramendia AA, Anegagrie M, Zewdie D, et al. Epidemiology of intestinal helminthiases in a rural community of Ethiopia: Is it time to expand control programs to include Strongyloides stercoralis and the entire community? *PLoS neglected tropical diseases.* 2020;14(6):e0008315.

555. Christian MA, Ruphin DD, Colette MA, Benjamin GZ, Gédéon BN, Emmanuel LM. Epidemiological profile of Ascariasis in Provincial General Reference Hospital of Kinshasa, Democratic Republic of the Congo. *Britain International of Exact Sciences (BIoEx) Journal.* 2020;2(2):541-546.

556. Gebereselassie Y, BirhanSelassie M, Menjetta T, Alemu J, Tsegaye A. Magnitude, Severity, and Associated Factors of Anemia among Under-Five Children Attending Hawassa University Teaching and Referral Hospital, Hawassa, Southern Ethiopia, 2016. *Anemia.* 2020;2020.

557. Hassan HA, Abd Alla AB, Elfaki TEM, Saad MBEA. Frequencies of gastrointestinal parasites among students of primary school in Al Kalakla Locality, Khartoum State, Sudan: a cross-sectional study. *F1000Research.* 2019;8.

558. Kpene G, Lokpo S, Deku J, Agboli E, Owiafe P. Asymptomatic Intestinal Parasitic Infestations among Children Under Five Years in Selected Communities in the Ho Municipality, Ghana. *Ethiopian Journal of Health Sciences.* 2020;30(6).

559. Odoemene S, Oluwole A, Mogaji H, et al. Polyparasitism with Malaria and Intestinal Parasite Infections among Infants and Preschool-Aged Children in Egbedore, Osun State, Nigeria. *Journal of Parasitology Research.* 2020;2020.

560. Ruth MMR, Cedric Y, Malla ME, et al. Intestinal Helminth Infections and Associated Risk Factors among School-Aged Children of Bamendjou Community, West Region of Cameroon. *Journal of Parasitology Research.* 2021;2021.

561. Ferreira FS, Pereira FdLM, Martins MdRO. Intestinal parasitic infections in children under five in the Central Hospital of Nampula, Northern Mozambique. *The Journal of Infection in Developing Countries.* 2020;14(05):532-539.

562. Sitotaw B, Shiferaw W. Prevalence of intestinal parasitic infections and associated risk factors among the first-cycle primary schoolchildren in Sasiga District, Southwest Ethiopia. *Journal of parasitology research.* 2020;2020.

563. Tiruneh T, Geshere G, Ketema T. Prevalence and determinants of soil-transmitted helminthic infections among school children at goro primary school, South West Shewa, Ethiopia. *International Journal of Pediatrics.* 2020;2020.

564. Yeshitila YG, Zewde H, Mekene T, Manilal A, Lakew S, Teshome A. Prevalence and associated risk factors of intestinal parasites among schoolchildren from two primary schools in Rama Town, Northern Ethiopia. *Canadian Journal of Infectious Diseases and Medical Microbiology.* 2020;2020.

565. Yoseph A, Beyene H. The high prevalence of intestinal parasitic infections is associated with stunting among children aged 6–59 months in Boricha Woreda, Southern Ethiopia: a cross-sectional study. *BMC Public Health.* 2020;20(1):1-13.

566. Abaka-Yawson A, Senoo D, Aboagye EA, et al. High prevalence of intestinal helminthic infection among children under 5 years in a rural Ghanaian community: an urgent call for attention. *Journal of Parasitic Diseases.* 2020;44(3):625-632.

567. Abebaw A, Alemu G, Ayehu A. Prevalence of intestinal parasites and associated factors among children from child centres in Bahir Dar city, northwest Ethiopia. *Tropical Doctor.* 2020;50(3):194-198.

568. Abudho BO, Guyah B, Ondigo BN, et al. Evaluation of morbidity in Schistosoma mansoni-positive primary and secondary school children after four years of mass drug administration of praziquantel in western Kenya. *Infectious Diseases of Poverty.* 2020;9(1):1-10.

569. Allan L, Mbai FN, Yole DS, Owino M. Intensity of nematode infection in children aged 3 to 5 Years living in mukuru kwa Njenga slum settlement, Nairobi, Kenya. *Journal of Tropical Medicine.* 2020;2020.

570. Hailu Amare H, Lindtjørn B. Helminth infections among rural schoolchildren in Southern Ethiopia: A cross-sectional multilevel and zero-inflated regression model. *PLoS neglected tropical diseases.* 2020;14(12):e0008002.

571. Asfaw MA, Gezmu T, Wegayehu T, et al. Soil-transmitted helminth infections among pre-school aged children in Gamo Gofa zone, Southern Ethiopia: Prevalence, intensity and intervention status. *PLoS One.* 2020;15(12):e0243946.

572. Chege N. The prevalence of intestinal parasites and associated risk factors in school-going children from informal settlements in Nakuru town, Kenya. *Malawi Medical Journal.* 2020;32(2):80-86.

573. Dejon-Agobé JC, Honkpehedji YJ, Zinsou JF, et al. Epidemiology of schistosomiasis and soil-transmitted helminth coinfections among schoolchildren living in Lambaréné, Gabon. *The American Journal of Tropical Medicine and Hygiene.* 2020;103(1):325.

574. Gebreyesus TD, Tadele T, Mekete K, et al. Prevalence, intensity, and correlates of schistosomiasis and soil-transmitted helminth infections after five rounds of preventive chemotherapy among school children in Southern Ethiopia. *Pathogens.* 2020;9(11):920.

575. Gitore WA, Ali MM, Yoseph A, Mangesha AE, Debiso AT. Prevalence of soil-transmitted helminthes and its association with water, sanitation, hygiene among schoolchildren and barriers for schools level prevention in technology villages of Hawassa University: Mixed design. *PLoS One.* 2020;15(9):e0239557.

576. Kabatende J, Mugisha M, Ntirenganya L, et al. Prevalence, intensity, and correlates of soil-transmitted helminth infections among school children after a decade of preventive chemotherapy in Western Rwanda. *Pathogens.* 2020;9(12):1076.

577. Kiiti RW, Omukunda EN, Korir JC. Risk Factors Associated with Helminthic Intestinal Infection in Lurambi Subcounty, Kakamega, Kenya. *Journal of Parasitology Research.* 2020;2020.

578. Kim JY, Sim S, Chung EJ, et al. Effectiveness of Mass Drug Administration on Neglected Tropical Diseases in Schoolchildren in Zanzibar, Tanzania. *The Korean Journal of Parasitology.* 2020;58(2):109.

579. Leta GT, Mekete K, Wuletaw Y, et al. National mapping of soil-transmitted helminth and schistosome infections in Ethiopia. *Parasites & vectors.* 2020;13(1):1-13.

580. Midzi N, Montresor A, Mutsaka-Makuvaza MJ, et al. Elimination of STH morbidity in Zimbabwe: Results of 6 years of deworming intervention for school-age children. *PLoS neglected tropical diseases.* 2020;14(10):e0008739.

581. Njambi E, Magu D, Masaku J, Okoyo C, Njenga SM. Prevalence of Intestinal Parasitic Infections and Associated Water, Sanitation, and Hygiene Risk Factors among School Children in Mwea Irrigation Scheme, Kirinyaga County, Kenya. *Journal of tropical medicine.* 2020;2020.

582. Ntonifor HN, Chewa JS, Oumar M, Mbouobda HD. Intestinal helminths as predictors of some malaria clinical outcomes and IL-1β levels in outpatients attending two public hospitals in Bamenda, North West Cameroon. *PLoS neglected tropical diseases.* 2021;15(3):e0009174.

583. Okoyo C, Campbell SJ, Williams K, Simiyu E, Owaga C, Mwandawiro C. Prevalence, intensity and associated risk factors of soil-transmitted helminth and schistosome infections in Kenya: impact assessment after five rounds of mass drug administration in Kenya. *PLoS neglected tropical diseases.* 2020;14(10):e0008604.

584. Palmeirim MS, Mrimi EC, Minja EG, Samson AJ, Keiser J. A cross-sectional survey on parasitic infections in schoolchildren in a rural Tanzanian community. *Acta Tropica.* 2021;213:105737.

585. Pion SD, Chesnais CB, Awaca-Uvon NP, et al. The impact of four years of semiannual treatments with albendazole alone on lymphatic filariasis and soil-transmitted helminth infections: A community-based study in the Democratic Republic of the Congo. *PLoS neglected tropical diseases.* 2020;14(6):e0008322.

586. Werunga DK, Omukunda EN, Korir JC. Prevalence and Intensity of Intestinal Helminth Infections in Preschool Pupils in Lugari Subcounty, Kakamega County, Kenya. *Journal of Parasitology Research.* 2020;2020.

587. Workineh L, Kiros T, Damtie S, Andualem T, Dessie B. Prevalence of soil-transmitted Helminth and Schistosoma mansoni infection and their associated factors among Hiruy Abaregawi primary school children, rural Debre Tabor, North West Ethiopia: A Cross-Sectional Study. *Journal of Parasitology Research.* 2020;2020.

588. Zeleke AJ, Bayih AG, Afework S, Gilleard JS. Treatment efficacy and re-infection rates of soil-transmitted helminths following mebendazole treatment in schoolchildren, Northwest Ethiopia. *Tropical Medicine and Health.* 2020;48(1):1-6.

589. Kjetland EF, Gundersen SG, Zulu SG, Taylor M. Prevalence and intensity of neglected tropical diseases (schistosomiasis and soil-transmitted helminths) amongst rural female pupils in Ugu district, KwaZulu-Natal, South Africa. *Southern African Journal of Infectious Diseases.* 2020;35(1):1-7.

590. Abera D, Wordofa M, Mesfin A, et al. Intestinal helminthic infection and allergic disorders among school children enrolled in mass deworming program, Sululta, Ethiopia. *Allergy, Asthma & Clinical Immunology.* 2021;17(1):1-11.

591. Belete YA, Kassa TY, Baye MF. Prevalence of intestinal parasite infections and associated risk factors among patients of Jimma health center requested for stool examination, Jimma, Ethiopia. *PLoS One.* 2021;16(2):e0247063.

592. Alula GA, Munshea A, Nibret E. Prevalence of Intestinal Parasitic Infections and Associated Risk Factors among Pregnant Women Attending Prenatal Care in the Northwestern Ethiopia. *BioMed Research International.* 2021;2021.

593. Aschale A, Adane M, Getachew M, et al. Water, sanitation, and hygiene conditions and prevalence of intestinal parasitosis among primary school children in Dessie City, Ethiopia. *PLoS One.* 2021;16(2):e0245463.

594. Cho FN, Ngala HN, Bongazi RT, et al. Effects of Soil-Transmitted Helminths and Intestinal Protozoan Infections on Haemoglobin Levels among School-Aged Children in Belo and Bui, North West Cameroon: A Cross-Sectional Study. *Journal of Parasitology Research.* 2021;2021.

595. Damtie D, Sitotaw B, Menkir S, Kerisew B, Hussien K. Human Intestinal Parasitic Infections: Prevalence and Associated Risk Factors among Elementary School Children in Merawi Town, Northwest Ethiopia. *Journal of Parasitology Research.* 2021;2021.

596. Eyayu T, Kiros T, Workineh L, et al. Prevalence of intestinal parasitic infections and associated factors among patients attending at Sanja Primary Hospital, Northwest Ethiopia: An institutional-based cross-sectional study. *PLoS One.* 2021;16(2):e0247075.

597. Fentahun A, Hailu T, Alemu G. Prevalence of Intestinal Parasites and Schistosoma mansoni and Associated Factors among Fishermen at Lake Tana, Northwest Ethiopia. *BioMed Research International.* 2021;2021.

598. Gasparinho C, Kanjungo A, Zage F, et al. Impact of Annual Albendazole versus Four-Monthly Test-and-Treat Approach of Intestinal Parasites on Children Growth—A Longitudinal Four-Arm Randomized Parallel Trial during Two Years of a Community Follow-Up in Bengo, Angola. *Pathogens.* 2021;10(3):309.

599. Hailu GG, Ayele ET. Assessment of the prevalence of intestinal parasitic infections and associated habit and culture-related risk factors among primary schoolchildren in Debre Berhan town, Northeast Ethiopia. *BMC Public Health.* 2021;21(1):1-12.

600. Pokorna-Kałwak D. Intestinal parasitic infections in a population of BaAka Pygmies inhabiting the Congo Basin in the Central African Republic. *Annals of Agricultural and Environmental Medicine.* 2021;28(1):127-130.

601. Makata K, Ensink J, Ayieko P, et al. Hand hygiene intervention to optimise soil-transmitted helminth infection control among primary school children: the Mikono Safi cluster randomised controlled trial in northwestern Tanzania. *BMC medicine.* 2021;19(1):1-13.

602. Shiferaw K, Tesfay T, Kalayu G, Kiros G. Human Intestinal Parasites: Prevalence and Associated Risk Factors among Grade School Children in Maksegnit, Northwest Ethiopia. *Journal of Tropical Medicine.* 2021;2021.

603. Tegen D, Damtie D. Prevalence and Risk Factors Associated with Intestinal Parasitic Infection among Primary School Children in Dera District, Northwest Ethiopia. *Canadian Journal of Infectious Diseases and Medical Microbiology.* 2021;2021.

604. Cedric Y, Nadia NAC, Payne VK, Sabi Bertrand M, Romeo NG. Gastrointestinal Nematodes among Residents in Melong, Moungo Division, Littoral Region, Cameroon. *BioMed Research International.* 2021;2021.

605. Andargie D, Tegegne Y, Worku L. Evaluation of Intestinal Parasite Infection in Low and High Coverage of Graduated Households, Northwest Ethiopia: A Comparative-Based Crosssectional Study. *Journal of parasitology research.* 2021;2021.

606. Aribodor OB, Ekwunife CA, Sam-Wobo SO, et al. Status of Intestinal Helminth Infection in Schools Implementing the Home-Grown School Feeding Program and the Impact of the Program on Pupils in Anambra State, Nigeria. *Acta Parasitologica.* 2021;66(4):1528-1537.

607. Ayele A, Tegegne Y, Derso A, Eshetu T, Zeleke AJ. Prevalence and associated factors of intestinal helminths among kindergarten children in Gondar town, northwest Ethiopia. *Pediatric Health, Medicine and Therapeutics.* 2021;12:35.

608. Bosch F, Palmeirim MS, Ali SM, Ame SM, Hattendorf J, Keiser J. Diagnosis of soil-transmitted helminths using the Kato-Katz technique: What is the influence of stirring, storage time and storage temperature on stool sample egg counts? *PLoS neglected tropical diseases.* 2021;15(1):e0009032.

609. Ejigu K, Hailu T, Alemu M. Efficacy of Mebendazole and Praziquantel against Soil-Transmitted Helminths and Schistosoma mansoni Infections among Schoolchildren in Northwest Ethiopia. *BioMed Research International.* 2021;2021.

610. Eltantawy M, Orsel K, Schroeder A, et al. Soil transmitted helminth infection in primary school children varies with ecozone in the Ngorongoro Conservation Area, Tanzania. *Tropical Medicine and Health.* 2021;49(1):1-12.

611. Eneanya OA, Gankpala L, Goss CW, Bolay FK, Weil GJ, Fischer PU. Impact of Annual versus Semiannual Mass Drug Administration with Ivermectin and Albendazole on Helminth Infections in Southeastern Liberia. *Am J Trop Med Hyg.* 2021;106(2):700-709.

612. Goshu A, Alemu G, Ayehu A. Prevalence and Intensity of Soil-Transmitted Helminths and Associated Factors among Adolescents and Adults in Bibugn Woreda, Northwest Ethiopia: A Community-Based Cross-Sectional Study. *Journal of Tropical Medicine.* 2021;2021.

613. Zeleke AJ, Derso A, Bayih AG, Gilleard JS, Eshetu T. Prevalence, Infection Intensity and Associated Factors of Soil-Transmitted Helminthiasis Among School-Aged Children from Selected Districts in Northwest Ethiopia. *Research and Reports in Tropical Medicine.* 2021;12:15.

614. Habib A, Andrianonimiadana L, Rakotondrainipiana M, et al. High prevalence of intestinal parasite infestations among stunted and control children aged 2 to 5 years old in two neighborhoods of Antananarivo, Madagascar. *PLoS neglected tropical diseases.* 2021;15(4):e0009333.

615. Grau-Pujol B, Cuamba I, Jairoce C, et al. Molecular Detection of Soil-Transmitted Helminths and Enteric Protozoa Infection in Children and Its Association with Household Water and Sanitation in Manhiça District, Southern Mozambique. *Pathogens.* 2021;10(7):838.

616. Fetene Y, Hailu T, Yimer M, Alemu M. Determinants of Helminthic Infections and Anemia among Schoolchildren in Bahir Dar Zuria District, Northwest Ethiopia. *Journal of Parasitology Research.* 2021;2021.
