# Supplementary material for: Global prevalence of Ascaris infection in humans (2010–2021): a systematic review and meta-analysis
Source: Infect Dis Poverty. 2022 Nov 18;11:113. doi: 10.1186/s40249-022-01038-z (PMC9673379; doi:10.1186/s40249-022-01038-z)
Supplement: Supplementary file 2 — Additional file 2: Table S2. The main characteristics of studies that included intensity of Ascaris infection. [file 40249_2022_1038_MOESM2_ESM.docx]

**Table S2.** The main characteristics of studies that included intensity of *Ascaris* infection

| First Author’s last name/ Pub year (Ref) | Type of population | Country | Sample size | Infected Total | Low intensity | Moderate intensity | High Intensity |
| --- | --- | --- | --- | --- | --- | --- | --- |
| Central and Southern Asia |  |  |  |  |  |  |  |
| Gunawardena et al. (2011)^1^ | Children | Sri Lanka | 1890 | 462 | 254 | 188 | 20 |
| Ranjan et al. (2013)^2^ | Children | India | 347 | 28 | 22 | 6 | 0 |
| Sherkhonov et al. (2013)^3^ | Children | Tajikestan | 1642 | 277 | 277 | 0 | 0 |
| Benjamin-Chung et al. (2015)^4^ | Children & adults | Bangladesh | 1630 | 214 | 193 | 21 | 0 |
| Ganguly et al. (2017)^5^ | Children | India | 6421 | 4134 | 3829 | 293 | 12 |
| Galgamuwa et al. (2018)^6^ | Children | Sri Lanka | 489 | 188 | 96 | 57 | 35 |
| Safi et al. (2019)^7^ | Children | Afghanistan | 2263 | 582 | 582 | 0 | 0 |
| Ajjampur et al. (2021)^8^ | Children & adults | India | 6089 | 6 | 6 | 0 | 0 |
| Eastern and South-Eastern Asia |  |  |  |  |  |  |  |
| Ziegelbauer et al. (2010)^9^ | Children | China | 268 | 106 | 42 | 60 | 4 |
| Manabo et al. (2010)^10^ | Children | Philippines | 102 | 33 | 33 | 0 | 0 |
| Kounnavong et al. (2011)^11^ | Children | Laos PDR | 570 | 156 | 48 | 44 | 64 |
| Ngui et al. (2011)^12^ | Children & adults | Malaysia | 716 | 276 | 117 | 142 | 10 |
| Yap et al. (2012)^13^ | Children | China | 69 | 30 | 19 | 8 | 3 |
| Wang et al. (2012)^14^ | Children | China | 1707 | 285 | 149 | 101 | 35 |
| Ngui et al. (2012)^15^ | Children | Malaysia | 550 | 229 | 92 | 124 | 8 |
| Ezeamama et al. (2012)^16^ | Children & adults | Philippines | 253 | 299 | 62 | 102 | 35 |
| Phongluxa et al. (2013)^17^ | Children & adults | Laos PDR | 574 | 56 | 35 | 15 | 6 |
| Pham-Duc et al. (2013)^18^ | Children & adults | Vietnam | 1425 | 342 | 335 | 7 | 0 |
| Du et al. (2014)^19^ | Children & adults | China | 289 | 154 | 150 | 4 | 0 |
| Eom et al. (2014)^20^ | Chidren & adults | Laos PDR | 6178 | 227 | 191 | 33 | 2 |
| Sayasone et al. (2015)^21^ | Children & adults | Laos PDR | 458 | 162 | 58 | 86 | 18 |
| Vonghachack et al. (2015)^22^ | Children & adults | Laos PDR | 729 | 2 | 2 | 0 | 0 |
| Gordon et al. (2015)^23^ | Children & adults | Philippines | 545 | 317 | 92 | 69 | 156 |
| Fang-Wei et al. (2016)^24^ | Children & adults | China | 1002 | 17 | 15 | 2 | 0 |
| Llewellyn et al. (2016)^25^ | Children & adults | Timor-Leste | 680 | 287 | 287 | 0 | 0 |
| de Gier et al. (2016)^26^ | Children | Vietnam | 510 | 327 | 116 | 198 | 13 |
| Vonghachack et al. (2017)^27^ | Children & adults | Laos PDR | 377 | 1 | 1 | 0 | 0 |
| Vonghachack et al. (2017)^28^ | Children & adults | Laos PDR | 994 | 6 | 5 | 1 | 0 |
| Mationg et al. (2017)^29^ | Children | Philippines | 263 | 52 | 25 | 23 | 4 |
| Liwanag et al. (2017)^30^ | Children & adults | Philippines | 951 | 292 | 134 | 126 | 29 |
| de Gier et al. (2018)^31^ | Children | Cambodia | 1795 | 5 | 5 | 0 | 0 |
| Dai et al. (2019)^32^ | Children & adults | China | 30,153 | 35 | 33 | 2 | 0 |
| Ansari Nasution et al. (2019)^33^ | Children | Indonesia | 298 | 202 | 157 | 45 | 0 |
| Muslim et al. (2019)^34^ | Population | Malaysia | 416 | 180 | 60 | 87 | 33 |
| Zhu et al. (2020)^35^ | Children & adults | China | 305,081 | 2338 | 1915 | 388 | 35 |
| Nasr et al. (2020)^36^ | Children | Malaysia | 1142 | 721 | 384 | 221 | 116 |
| Dukpa et al. (2020)^37^ | Children | Bhutan | 1456 | 11 | 10 | 1 | 0 |
| Jin-rong et al. (2021)^38^ | Children & adults | China | 5067 | 381 | 259 | 111 | 11 |
| Feng et al. (2021)^39^ | Chidren & adults | China | 23,552 | 13 | 12 | 1 | 0 |
| Bria et al. (2021)^40^ | Children | Indonesia | 130 | 50 | 10 | 40 | 0 |
| Djuardi et al. (2021)^41^ | Children | Indonesia | 393 | 129 | 34 | 64 | 31 |
| Aung et al. (2021)^42^ | Children | Myanmar | 363 | 41 | 21 | 16 | 4 |
| Labana et al. (2021)^43^ | Children | Philippines | 478 | 98 | 64 | 20 | 14 |
| Wattanawong et al. (2021)^44^ | Children & adults | Thailand | 16,187 | 33 | 31 | 2 | 0 |
| Latin America and the Caribbean |  |  |  |  |  |  |  |
| Rosewell et al. (2010)^45^ | Children | Nicaragua | 880 | 182 | 157 | 23 | 2 |
| Lander et al. (2012)^46^ | Children | Brazil | 376 | 34 | 17 | 9 | 8 |
| Canete et al. (2012)^47^ | Children | Cuba | 104 | 7 | 7 | 0 | 0 |
| Verhagen et al. (2013)^48^ | Children | Venezuela | 390 | 104 | 52 | 20 | 32 |
| Bragagnoli et al. (2014)^49^ | Children | Brazil | 1004 | 260 | 105 | 32 | 123 |
| Capon-Robins et al. (2014)^50^ | Children & adults | Eucador | 211 | 102 | 46 | 52 | 4 |
| Gabrie et al. (2014)^51^ | Children | Honduras | 320 | 97 | 39 | 52 | 6 |
| Torres et al. (2014)^52^ | Children | Honduras | 2554 | 569 | 312 | 233 | 24 |
| Periago et al. (2015)^53^ | Children | Brazil | 1260 | 252 | 94 | 121 | 37 |
| Da Silva et al. (2016)^54^ | Children & adults | Brazil | 597 | 292 | 128 | 144 | 20 |
| Canete et al. (2017)^55^ | Children | Cuba | 107 | 21 | 21 | 0 | 0 |
| de Gier et al. (2018)^31^ | Children | Cuba | 1389 | 72 | 55 | 15 | 0 |
| Moncayo et al. (2018)^56^ | Children | Ecuador | 920 | 170 | 79 | 66 | 25 |
| Muñoz-Antoli et al. (2018)^57^ | Children | Nicaragua | 341 | 68 | 42 | 23 | 3 |
| Guttierrez-Jimenez et al. (2019)^58^ | Children | Mexico | 84 | 48 | 13 | 18 | 4 |
| Gildner et al. (2020)^59^ | Children & adults | Ecuador | 620 | 301 | 143 | 146 | 12 |
| Calegar et al. (2021)^60^ | Children | Brazil | 349 | 61 | 34 | 23 | 4 |
| Incani et al. (2021)^61^ | Children & adults | Venezuela | 224 | 89 | 33 | 42 | 14 |
| Chura et al. (2021)^62^ | Children | Bolivia | 275 | 40 | 30 | 8 | 2 |
| Oceania |  |  |  |  |  |  |  |
| Bradbury et al. (2018)^63^ | Children & adults | Soloman Islands | 583 | 311 | 150 | 146 | 15 |
| Sub-Saharan Africa |  |  |  |  |  |  |  |
| Kurup et al. (2010)^64^ | Children | St Lucia | 554 | 97 | 79 | 12 | 6 |
| Knopp et al. (2010)^65^ | Children & adults | Zanzibar | 270 | 139 | 120 | 18 | 0 |
| Midzi et al. (2010)^66^ | Children | Zimbawe | 575 | 12 | 8 | 4 | 0 |
| Richardson et al. (2011)^67^ | Children & adults | Cameroon | 94 | 31 | 27 | 4 | 0 |
| Richardson et al. (2011)^67^ | Children & adults | Cameroon | 275 | 42 | 25 | 13 | 4 |
| Furst et al. (2011)^68^ | Children | Côte d'Ivoire | 167 | 2 | 1 | 1 | 0 |
| Alemu et al. (2011)^69^ | Children | Ethiopia | 319 | 70 | 20 | 40 | 10 |
| Hodges et al. (2011)^70^ | Children | Guinea | 420 | 34 | 32 | 2 | 0 |
| Odiere et al. (2011)^71^ | Children | Kenya | 1308 | 64 | 54 | 10 | 0 |
| Odiere et al. (2012)^72^ | Children | Kenya | 4065 | 134 | 108 | 26 | 0 |
| Friedman et al. (2012)^73^ | Children | Tanzania | 352 | 24 | 16 | 8 | 0 |
| Tchuenté et al. (2013)^74^ | Children & adults | Cameroon | 4130 | 805 | 471 | 281 | 53 |
| Schmidlin et al. (2013)^75^ | Children & adults | Côte d'Ivoire | 1992 | 15 | 8 | 1 | 0 |
| Tekeste et al. (2013)^76^ | Children | Ethiopia | 326 | 54 | 54 | 0 | 0 |
| Hurliman et al. (2014)^77^ | Children | Cote d'Ivoire | 4848 | 89 | 75 | 14 | 0 |
| Matangila et al. (2014)^78^ | Children | DR Congo | 650 | 95 | 85 | 10 | 0 |
| G/hiwot et al. (2014)^79^ | Children | Ethiopia | 374 | 13 | 9 | 4 | 0 |
| Schule et al. (2014)^80^ | Children & adults | Tanzania | 6366 | 433 | 317 | 66 | 50 |
| Bird et al. (2014)^81^ | Children | Zanzibar | 915 | 302 | 193 | 109 | 0 |
| Kuete et al. (2015)^82^ | Children & adults | Cameroon | 428 | 9 | 9 | 0 | 0 |
| Emana et al. (2015)^83^ | Children | Ethiopia | 302 | 60 | 59 | 1 | 0 |
| Shumbej et al. (2015)^84^ | Children | Ethiopia | 377 | 56 | 55 | 1 |  |
| Alelign et al. (2015)^85^ | Children | Ethiopia | 384 | 53 | 53 | 0 | 0 |
| Alemayehu et al. (2015)^86^ | Children | Ethiopia | 384 | 21 | 21 | 0 | 0 |
| Jejaw et al. (2015)^87^ | Children | Ethiopia | 460 | 132 | 76 | 49 | 7 |
| Gashaw et al. (2015)^88^ | Children | Ethiopia | 550 | 194 | 149 | 45 | 0 |
| Boko et al. (2016)^89^ | Children | Benin | 7500 | 581 | 544 | 31 | 6 |
| Ortu et al. (2016)^90^ | Children | Burundi | 650 | 117 | 61 | 14 | 0 |
| Ortu et al. (2016)^90^ | Children | Burundi | 4680 | 749 | 452 | 60 | 0 |
| Ortu et al. (2016)^90^ | Children | Burundi | 5062 | 861 | 456 | 57 | 0 |
| Bopda et al. (2016)^91^ | Adults | Cameroon | 334 | 60 | 60 | 0 | 0 |
| Alemu et al. (2016)^92^ | Children | Ethiopia | 401 | 77 | 40 | 31 | 6 |
| Mekonnen et al. (2016)^93^ | Children & adults | Ethiopia | 1021 | 105 | 103 | 0 | 2 |
| Ferreira et al. (2016)^94^ | Children | Sao Tome and Principe | 444 | 250 | 80 | 97 | 31 |
| Müller et al. (2016)^95^ | Children | South Africa | 934 | 248 | 72 | 125 | 51 |
| Fuhrimann et al. (2016)^96^ | Adults | Uganda | 915 | 53 | 41 | 12 | 0 |
| Ngole Sumbele et al. (2017)^97^ | Children & adults | Cameroon | 450 | 54 | 47 | 6 | 1 |
| da Luz et al. (2017)^98^ | Children | DR Congo | 224 | 36 | 36 | 0 | 0 |
| da Luz et al. (2017)^98^ | Children | DR Congo | 253 | 13 | 13 | 0 | 0 |
| Kabore et al. (2017)^99^ | Children | DR Congo | 1300 | 205 | 131 | 73 | 1 |
| Tefera et al. (2017)^100^ | Children | Ethiopia | 715 | 169 | 156 | 10 | 3 |
| Shittu et al. (2017)^101^ | Children & adults | Nigeria | 696 | 311 | 311 | 0 | 0 |
| Adriko et al. (2017)^102^ | Children | Uganda | 4285 | 22 | 19 | 3 | 0 |
| Ibikounle et al. (2018)^103^ | Children | Benin | 19,250 | 1030 | 869 | 147 | 14 |
| Coulibaly et al. (2018)^104^ | Children & adults | Cote d'Ivoire | 812 | 3 | 3 | 0 | 0 |
| Coulibaly et al. (2018)^104^ | Children & adults | Cote d'Ivoire | 1046 | 1 | 1 | 0 | 0 |
| Coulibaly et al. (2018)^104^ | Children & adults | Cote d'Ivoire | 2447 | 8 | 5 | 2 | 0 |
| Ibrahim et al. (2018)^105^ | Children | Ethiopia | 340 | 56 | 56 | 0 | 0 |
| Adu-Gyasi et al. (2018)^106^ | Children & adults | Ghana | 1569 | 23 | 19 | 4 | 0 |
| Olopade et al. (2018)^107^ | Children | Nigeria | 384 | 85 | 79 | 6 | 0 |
| Babamale et al. (2018)^108^ | Children & adults | Nigeria | 471 | 297 | 169 | 90 | 38 |
| Bronzan et al. (2018)^109^ | Children | Togo | 17,097 | 63 | 53 | 10 | 0 |
| Tekalign et al. (2019)^110^ | Population | Ethiopia | 377 | 58 | 41 | 17 | 0 |
| Weldesenbet et al. (2019)^111^ | Children | Ethiopia | 600 | 18 | 18 | 0 | 0 |
| Ihejirika et al. (2019)^112^ | Children | Nigeria | 300 | 12 | 9 | 2 | 1 |
| Bah et al. (2019)^113^ | Children | Sierra Leone | 3632 | 160 | 148 | 9 | 3 |
| Rosine Ruth et al. (2020)^114^ | Children | Cameroon | 493 | 32 | 13 | 8 | 11 |
| Abebaw et al. (2020)^115^ | Children | Ethiopia | 217 | 9 | 9 | 0 | 0 |
| Workineh et al. (2020)^116^ | Children | Ethiopia | 340 | 28 | 24 | 4 | 0 |
| Zeleke et al. (2020)^117^ | Children | Ethiopia | 504 | 64 | 46 | 17 | 1 |
| Amare et al. (2020)^118^ | Children | Ethiopia | 850 | 159 | 158 | 1 | 0 |
| Asfaw et al. (2020)^119^ | Children | Ethiopia | 2462 | 457 | 388 | 68 | 0 |
| Gebreyesus et al. (2020)^120^ | Children | Ethiopia | 3162 | 1515 | 933 | 452 | 130 |
| Dejon-Agobe et al. (2020)^121^ | Children | Gabon | 472 | 15 | 15 | 0 | 0 |
| Werunga et al. (2020)^122^ | Children | Kenya | 130 | 16 | 14 | 2 | 0 |
| Allan et al. (2020)^123^ | Children | Kenya | 172 | 60 | 39 | 13 | 8 |
| Allan et al. (2020)^123^ | Children | Kenya | 172 | 60 | 38 | 22 | 0 |
| Chege et al. (2020)^124^ | Children | Kenya | 248 | 1 | 1 | 0 | 0 |
| Kabatende et al. (2020)^125^ | Children | Rwanda | 4998 | 2499 | 1804 | 640 | 55 |
| Midzi et al. (2020)^126^ | Zimbabwe | Zimbawe | 13,195 | 343 | 327 | 8 | 8 |
| Ntonifor et al. (2021)^127^ | Children & adults | Cameroon | 358 | 23 | 22 | 1 | 0 |
| Sumbele et al. (2021)^128^ | Children | Cameroon | 638 | 26 | 26 | 0 | 0 |
| Cedric et al. (2021)^129^ | Children & adults | Cameroon | 788 | 17 | 8 | 9 | 0 |
| Ayele et al. (2021)^130^ | Children | Ethiopia | 390 | 35 | 34 | 1 | 0 |
| Ejigu et al. (2021)^131^ | Children | Ethiopia | 422 | 89 | 89 | 0 | 0 |
| Andargie et al. (2021)^132^ | Children & adults | Ethiopia | 478 | 56 | 36 | 13 | 7 |
| Abera et al. (2021)^133^ | Children | Ethiopia | 526 | 18 | 18 | 0 | 0 |
| Zeleke et al. (2021)^134^ | Children | Ethiopia | 786 | 191 | 138 | 50 | 3 |
| Aribodor et al. (2021)^135^ | Children | Nigeria | 1677 | 74 | 72 | 2 | 0 |
| Brandt et al. (2021)^136^ | Children | South Africa | 587 | 112 | 69 | 37 | 6 |
| Bosch et al. (2021)^137^ | Children | Tanzania | 92 | 38 | 14 | 24 | 0 |

**References**

1. Gunawardena K, Kumarendran B, Ebenezer R, Gunasingha MS, Pathmeswaran A, De Silva N. Soil-transmitted helminth infections among plantation sector schoolchildren in Sri Lanka: prevalence after ten years of preventive chemotherapy. *PLoS Neglected Tropical Diseases.* 2011;5(9):e1341.

2. Ranjan S, Passi SJ, Singh SN. Prevalence and risk factors associated with the presence of Soil-Transmitted Helminths in children studying in Municipal Corporation of Delhi Schools of Delhi, India. *Journal of parasitic diseases.* 2015;39(3):377-384.

3. Sherkhonov T, Yap P, Mammadov S, et al. National intestinal helminth survey among schoolchildren in Tajikistan: prevalences, risk factors and perceptions. *Acta tropica.* 2013;126(2):93-98.

4. Benjamin-Chung J, Nazneen A, Halder AK, et al. The interaction of deworming, improved sanitation, and household flooring with soil-transmitted helminth infection in rural Bangladesh. *PLoS neglected tropical diseases.* 2015;9(12):e0004256.

5. Ganguly S, Barkataki S, Karmakar S, et al. High prevalence of soil-transmitted helminth infections among primary school children, Uttar Pradesh, India, 2015. *Infectious diseases of poverty.* 2017;6(1):1-9.

6. Galgamuwa LS, Iddawela D, Dharmaratne SD. Prevalence and intensity of Ascaris lumbricoides infections in relation to undernutrition among children in a tea plantation community, Sri Lanka: a cross-sectional study. *BMC pediatrics.* 2018;18(1):1-9.

7. Safi N, Warusavithana S, Alawi SAS, Atta H, Montresor A, Gabrielli AF. Elimination of morbidity due to soil-transmitted helminthiases among Afghan schoolchildren. *Acta tropica.* 2019;197:105035.

8. Ajjampur SS, Kaliappan SP, Halliday KE, et al. Epidemiology of soil transmitted helminths and risk analysis of hookworm infections in the community: Results from the DeWorm3 Trial in southern India. *PLoS neglected tropical diseases.* 2021;15(4):e0009338.

9. Ziegelbauer K, Steinmann P, Zhou H, et al. Self-rated quality of life and school performance in relation to helminth infections: case study from Yunnan, People's Republic of China. *Parasites & vectors.* 2010;3(1):1-11.

10. Manabo CA, Frias MVG. The antihelminthic efficacy of pineapple fruit mebendazole on soil transmitted helminthiases: a randomized controlled trial. *PIDSP Journal.* 2010;11(1):35-43.

11. Kounnavong S, Vonglokham M, Houamboun K, Odermatt P, Boupha B. Soil-transmitted helminth infections and risk factors in preschool children in southern rural Lao People's Democratic Republic. *Transactions of the Royal Society of Tropical Medicine and Hygiene.* 2011;105(3):160-166.

12. Ngui R, Ishak S, Chuen CS, Mahmud R, Lim YA. Prevalence and risk factors of intestinal parasitism in rural and remote West Malaysia. *PLoS Neglected Tropical Diseases.* 2011;5(3):e974.

13. Yap P, Du Z-W, Chen R, et al. Soil-transmitted helminth infections and physical fitness in school-aged Bulang children in southwest China: results from a cross-sectional survey. *Parasites & vectors.* 2012;5(1):1-9.

14. Wang X, Zhang L, Luo R, et al. Soil-Transmitted Helminth Infections and Correlated Risk Factors in Preschool and School-Aged Children in Rural Southwest China. *PLoS One.* 2012;7(9):e45939.

15. Ngui R, Lim YAL, Chong Kin L, Sek Chuen C, Jaffar S. Association between anaemia, iron deficiency anaemia, neglected parasitic infections and socioeconomic factors in rural children of West Malaysia. *PLoS neglected tropical diseases.* 2012;6(3):e1550.

16. Ezeamama AE, McGarvey ST, Hogan J, et al. Treatment for Schistosoma japonicum, reduction of intestinal parasite load, and cognitive test score improvements in school-aged children. *PLoS neglected tropical diseases.* 2012;6(5):e1634.

17. Phongluxa K, Xayaseng V, Vonghachack Y, Akkhavong K, van Eeuwijk P, Odermatt P. Helminth infection in southern Laos: high prevalence and low awareness. *Parasites & vectors.* 2013;6(1):1-15.

18. Pham-Duc P, Nguyen-Viet H, Hattendorf J, et al. Ascaris lumbricoides and Trichuris trichiura infections associated with wastewater and human excreta use in agriculture in Vietnam. *Parasitology international.* 2013;62(2):172-180.

19. Zun-Wei D, Jin-Yong J, Hong-Bin L, Ran C, Xue-Zhong W, Tian-You D. Investigation of prevalence of soil-transmitted nematode infections among Lahu Ethnic residents in Xiaojie Township, Jinghong City, Yunnan Province. *Chinese Journal of Schistosomiasis Control.* 2014;26(1):75.

20. Eom KS, Yong T-S, Sohn W-M, et al. Prevalence of helminthic infections among inhabitants of Lao PDR. *The Korean Journal of Parasitology.* 2014;52(1):51.

21. Sayasone S, Utzinger J, Akkhavong K, Odermatt P. Repeated stool sampling and use of multiple techniques enhance the sensitivity of helminth diagnosis: a cross-sectional survey in southern Lao People's Democratic Republic. *Acta tropica.* 2015;141:315-321.

22. Vonghachack Y, Sayasone S, Bouakhasith D, Taisayavong K, Akkavong K, Odermatt P. Epidemiology of Strongyloides stercoralis on Mekong islands in southern Laos. *Acta tropica.* 2015;141:289-294.

23. Gordon CA, McManus DP, Acosta LP, et al. Multiplex real-time PCR monitoring of intestinal helminths in humans reveals widespread polyparasitism in Northern Samar, the Philippines. *International journal for parasitology.* 2015;45(7):477-483.

24. Fang-Wei W, Li-Bo W, Ran C, et al. Investigation of soil-transmitted nematode infections in Xiding Township, Menghai County, Yunnan Province. *Zhongguo xue xi Chong Bing Fang zhi za zhi= Chinese Journal of Schistosomiasis Control.* 2016;29(1):93-95.

25. Llewellyn S, Inpankaew T, Nery SV, et al. Application of a multiplex quantitative PCR to assess prevalence and intensity of intestinal parasite infections in a controlled clinical trial. *PLoS neglected tropical diseases.* 2016;10(1):e0004380.

26. De Gier B, Nga TT, Winichagoon P, et al. Species-specific associations between soil-transmitted helminths and micronutrients in Vietnamese schoolchildren. *The American journal of tropical medicine and hygiene.* 2016;95(1):77.

27. Vonghachack Y, Sayasone S, Khieu V, et al. Comparison of novel and standard diagnostic tools for the detection of Schistosoma mekongi infection in Lao People's Democratic Republic and Cambodia. *Infectious diseases of poverty.* 2017;6(04):94-106.

28. Vonghachack Y, Odermatt P, Taisayyavong K, Phounsavath S, Akkhavong K, Sayasone S. Transmission of Opisthorchis viverrini, Schistosoma mekongi and soil-transmitted helminthes on the Mekong Islands, Southern Lao PDR. *Infectious Diseases of Poverty.* 2017;6(1):1-15.

29. Mationg MLS, Gordon CA, Tallo VL, et al. Status of soil-transmitted helminth infections in schoolchildren in Laguna Province, the Philippines: Determined by parasitological and molecular diagnostic techniques. *PLoS neglected tropical diseases.* 2017;11(11):e0006022.

30. Liwanag HJ, Uy J, Bataller R, et al. Soil-transmitted helminthiasis and schistosomiasis in children of poor families in Leyte, Philippines: lessons for disease prevention and control. *Journal of tropical pediatrics.* 2017;63(5):335-345.

31. de Gier B, Pita-Rodríguez GM, Campos-Ponce M, et al. Soil-transmitted helminth infections and intestinal and systemic inflammation in schoolchildren. *Acta tropica.* 2018;182:124-127.

32. Dai Y, Xu X, Liu J, et al. Prevalence of intestinal helminth infections in Jiangsu Province, eastern China; a cross-sectional survey conducted in 2015. *BMC infectious diseases.* 2019;19(1):1-9.

33. Nasution RKA, Nasution BB, Lubis M, Lubis IND. Prevalence and knowledge of soil-transmitted helminth infections in Mandailing Natal, North Sumatera, Indonesia. *Open Access Macedonian Journal of Medical Sciences.* 2019;7(20):3443.

34. Muslim A, Mohd Sofian S, Shaari SA, Hoh B-P, Lim YA-L. Prevalence, intensity and associated risk factors of soil transmitted helminth infections: A comparison between Negritos (indigenous) in inland jungle and those in resettlement at town peripheries. *PLoS neglected tropical diseases.* 2019;13(4):e0007331.

35. Zhu H-H, Zhou C-H, Zhu T-J, et al. Prevalence of soil-borne nematode infections among residents living in urban/town areas of China in 2015. *Zhongguo xue xi Chong Bing Fang zhi za zhi= Chinese Journal of Schistosomiasis Control.* 2020;32(5):476-482.

36. Nasr NA, Al-Mekhlafi HM, Lim YA, et al. A holistic approach is needed to control the perpetual burden of soil-transmitted helminth infections among indigenous schoolchildren in Malaysia. *Pathogens and Global Health.* 2020;114(3):145-159.

37. Dukpa T, Dorji N, Thinley S, et al. Soil-transmitted helminth infections reduction in Bhutan: a report of 29 years of deworming. *PLoS One.* 2020;15(1):e0227273.

38. Jin-rong Z, Li-bo W, Ya-ming Y, et al. Current status of Ascaris lumbricoides infection in populations in Yunan Province, 2015. *CHINESE JOURNAL OF PARASITOLOGY AND PARASITIC DISEASES.* 2021;39(2):273.

39. Feng Y, Yu K, Chen H, et al. Soil-transmitted helminths, intestinal protozoa and Clonorchis sinensis infections in southeast China. *BMC infectious diseases.* 2021;21(1):1-11.

40. Bria M, Arwati H, Tantular IS. Prevalence and risk factors of Ascaris lumbricoides infection in children of Manusak Village, Kupang District, East Nusa Tenggara Province, Indonesia. *Qanun Medika-Medical Journal Faculty of Medicine Muhammadiyah Surabaya.* 2021;5(2).

41. Djuardi Y, Lazarus G, Stefanie D, Fahmida U, Ariawan I, Supali T. Soil-transmitted helminth infection, anemia, and malnutrition among preschool-age children in Nangapanda subdistrict, Indonesia. *PLoS neglected tropical diseases.* 2021;15(6):e0009506.

42. Aung E, Han KT, Gordon CA, et al. High prevalence of soil-transmitted helminth infections in Myanmar schoolchildren. *Infectious Diseases of Poverty.* 2022;11(1):1-12.

43. Labana RV, Romero VA, Guinto AM, et al. Prevalence and intensity of soil-transmitted helminth infections among school-age children in the Cagayan Valley, the Philippines. *Asian Pacific Journal of Tropical Medicine.* 2021;14(3):113.

44. Wattanawong O, Iamsirithaworn S, Kophachon T, et al. Current status of helminthiases in Thailand: A cross-sectional, nationwide survey, 2019. *Acta Tropica.* 2021;223:106082.

45. Rosewell A, Robleto G, Rodrí, et al. Soil-transmitted helminth infection and urbanization in 880 primary school children in Nicaragua, 2005. *Tropical doctor.* 2010;40(3):141-143.

46. Lander RL, Lander AG, Houghton L, et al. Factors influencing growth and intestinal parasitic infections in preschoolers attending philanthropic daycare centers in Salvador, Northeast Region of Brazil. *Cadernos de saude publica.* 2012;28:2177-2188.

47. Cañete R, Díaz MM, Avalos García R, Laúd Martinez PM, Manuel Ponce F. Intestinal parasites in children from a day care centre in Matanzas City, Cuba. *PLoS One.* 2012;7(12):e51394.

48. Verhagen LM, Incani RN, Franco CR, et al. High malnutrition rate in Venezuelan Yanomami compared to Warao Amerindians and Creoles: significant associations with intestinal parasites and anemia. *PLoS One.* 2013;8(10):e77581.

49. Bragagnoli G, Silva MTN. Ascaris lumbricoides infection and parasite load are associated with asthma in children. *The Journal of Infection in Developing Countries.* 2014;8(07):891-897.

50. Cepon-Robins TJ, Liebert MA, Gildner TE, et al. Soil-transmitted helminth prevalence and infection intensity among geographically and economically distinct Shuar communities in the Ecuadorian Amazon. *The Journal of Parasitology.* 2014;100(5):598-607.

51. Gabrie JA, Rueda MM, Canales M, Gyorkos TW, Sanchez AL. School hygiene and deworming are key protective factors for reduced transmission of soil-transmitted helminths among schoolchildren in Honduras. *Parasites & vectors.* 2014;7(1):1-15.

52. Mejia Torres RE, Franco Garcia DN, Fontecha Sandoval GA, et al. Prevalence and intensity of soil-transmitted helminthiasis, prevalence of malaria and nutritional status of school going children in Honduras. *PLoS neglected tropical diseases.* 2014;8(10):e3248.

53. Periago MV, Diniz RC, Pinto SA, et al. The right tool for the job: detection of soil-transmitted helminths in areas co-endemic for other helminths. *PLoS neglected tropical diseases.* 2015;9(8):e0003967.

54. Da Silva JB, Bossolani GDP, Piva C, et al. Spatial distribution of intestinal parasitic infections in a Kaingang indigenous village from Southern Brazil. *International journal of environmental health research.* 2016;26(5-6):578-588.

55. Cañete R, Campos Y, Valdes R, Rodriguez P. Prevalence and factors associated with intestinal parasitic infection among schoolchildren from Jagüey Grande Municipality in Matanzas Province, Cuba. *West Indian Med J.* 2017;66(2):361-366.

56. Moncayo AL, Lovato R, Cooper PJ. Soil-transmitted helminth infections and nutritional status in Ecuador: findings from a national survey and implications for control strategies. *BMJ open.* 2018;8(4):e021319.

57. Muñoz-Antoli C, Pérez P, Pavón A, Toledo R, Esteban J-G. Soil-transmitted helminth infections and anemia in schoolchildren from Corn Island Archipelago (RAAS, Nicaragua). *The American journal of tropical medicine and hygiene.* 2018;99(6):1591.

58. Gutiérrez-Jiménez J, Luna-Cázares LM, Martínez-de la Cruz L, et al. Children from a rural region in The Chiapas Highlands, Mexico, show an increased risk of stunting and intestinal parasitoses when compared with urban children. *Boletín médico del Hospital Infantil de México.* 2019;76(1):18-26.

59. Gildner TE, Cepon-Robins TJ, Liebert MA, et al. Market integration and soil-transmitted helminth infection among the Shuar of Amazonian Ecuador. *PLoS One.* 2020;15(7):e0236924.

60. Calegar DA, Bacelar PA, Monteiro KJ, et al. A community-based, cross-sectional study to assess interactions between income, nutritional status and enteric parasitism in two Brazilian cities: are we moving positively towards 2030? *Journal of Health, Population and Nutrition.* 2021;40(1):1-10.

61. Incani RN, Grillet ME, Mughini-Gras L. Hotspots and correlates of soil-transmitted helminth infections in a Venezuelan rural community: Which are the “wormy” houses? *Journal of Infection.* 2021;82(1):143-149.

62. Chura JA, Macchioni F, Furzi F, et al. Cross-sectional study on intestinal parasite infections in different ecological zones of the Department of La Paz, Bolivia. *One Health.* 2021;13:100271.

63. Bradbury RS, Harrington H, Kekeubata E, et al. High prevalence of ascariasis on two coral atolls in the Solomon Islands. *Transactions of The Royal Society of Tropical Medicine and Hygiene.* 2018;112(4):193-199.

64. Kurup R, Hunjan GS. Epidemiology and control of Schistosomiasis and other intestinal parasitic infections among school children in three rural villages of south Saint Lucia. *Journal of Vector Borne Diseases.* 2010;47(4):228.

65. Knopp S, Mohammed KA, Stothard JR, et al. Patterns and risk factors of helminthiasis and anemia in a rural and a peri-urban community in Zanzibar, in the context of helminth control programs. *PLoS neglected tropical diseases.* 2010;4(5):e681.

66. Midzi N, Mtapuri-Zinyowera S, Mapingure M, et al. Consequences of polyparasitism on anaemia among primary school children in Zimbabwe. *Acta tropica.* 2010;115(1-2):103-111.

67. Richardson DJ, Richardson KR, Callahan KD, et al. Geohelminth infection in rural Cameroonian villages. *Comparative Parasitology.* 2011;78(1):161-179.

68. Fürst T, Müller I, Coulibaly JT, Yao AK, Utzinger J, N'Goran EK. Questionnaire-based approach to assess schoolchildren's physical fitness and its potential role in exploring the putative impact of helminth and Plasmodium spp. infections in Côte d'Ivoire. *Parasites & vectors.* 2011;4(1):1-10.

69. Alemu A, Atnafu A, Addis Z, et al. Soil transmitted helminths and Schistosoma mansoni infections among school children in Zarima town, northwest Ethiopia. *BMC infectious diseases.* 2011;11(1):1-7.

70. Hodges M, Koroma MM, Baldé MS, et al. Current status of schistosomiasis and soil-transmitted helminthiasis in Beyla and Macenta Prefectures, Forest Guinea. *Transactions of the Royal Society of Tropical Medicine and Hygiene.* 2011;105(11):672-674.

71. Odiere MR, Opisa S, Odhiambo G, et al. Geographical distribution of schistosomiasis and soil-transmitted helminths among school children in informal settlements in Kisumu City, Western Kenya. *Parasitology.* 2011;138(12):1569-1577.

72. Odiere MR, Rawago FO, Ombok M, et al. High prevalence of schistosomiasis in Mbita and its adjacent islands of Lake Victoria, western Kenya. *Parasites & vectors.* 2012;5(1):1-8.

73. Friedman AJ, Ali SM, Albonico M. Safety of a new chewable formulation of mebendazole for preventive chemotherapy interventions to treat young children in countries with moderate-to-high prevalence of soil transmitted helminth infections. *Journal of tropical medicine.* 2012;2012.

74. Tchuem Tchuenté L-A, Dongmo Noumedem C, Ngassam P, et al. Mapping of schistosomiasis and soil-transmitted helminthiasis in the regions of Littoral, North-West, South and South-West Cameroon and recommendations for treatment. *BMC Infectious Diseases.* 2013;13(1):1-12.

75. Schmidlin T, Hürlimann E, Silué KD, et al. Effects of hygiene and defecation behavior on helminths and intestinal protozoa infections in Taabo, Côte d’Ivoire. *PLoS One.* 2013;8(6):e65722.

76. Tekeste Z, Belyhun Y, Gebrehiwot A, et al. Epidemiology of intestinal schistosomiasis and soil transmitted helminthiasis among primary school children in Gorgora, Northwest Ethiopia. *Asian Pacific Journal of Tropical Disease.* 2013;3(1):61-64.

77. Hürlimann E, Houngbedji CA, Yapi RB, et al. Health-related quality of life among school children with parasitic infections: findings from a national cross-sectional survey in Côte d'Ivoire. *PLoS neglected tropical diseases.* 2014;8(12):e3287.

78. Matangila JR, Doua JY, Linsuke S, et al. Malaria, schistosomiasis and soil transmitted helminth burden and their correlation with anemia in children attending primary schools in Kinshasa, Democratic Republic of Congo. *PLoS One.* 2014;9(11):e110789.

79. G/hiwot Y, Degarege A, Erko B. Prevalence of intestinal parasitic infections among children under five years of age with emphasis on Schistosoma mansoni in Wonji Shoa Sugar Estate, Ethiopia. *PLoS One.* 2014;9(10):e109793.

80. Schüle SA, Clowes P, Kroidl I, et al. Ascaris lumbricoides infection and its relation to environmental factors in the Mbeya region of Tanzania, a cross-sectional, population-based study. *PLoS One.* 2014;9(3):e92032.

81. Bird C, Ame S, Albonico M, Bickle Q. Do shoes reduce hookworm infection in school-aged children on Pemba Island, Zanzibar? A pragmatic trial. *Transactions of The Royal Society of Tropical Medicine and Hygiene.* 2014;108(5):297-304.

82. Kuete T, Yemeli FLS, Mvoa EE, Nkoa T, Somo RM, Ekobo AS. Prevalence and risk factors of intestinal helminth and protozoa infections in an urban setting of Cameroon: the case of Douala. *Am J Epidemiol Infect Dis.* 2015;3(2):36-44.

83. Emana D, Jemal K, Bajiro M, Mekonnen Z. Prevalence and intensity of soil-transmitted helminths among school-aged children in Sigmo primary school, Jimma Zone, South-Western Ethiopia. *Clinical Medicine Research.* 2015;4(4):98-103.

84. Shumbej T, Belay T, Mekonnen Z, Tefera T, Zemene E. Soil-transmitted helminths and associated factors among pre-school children in Butajira Town, South-Central Ethiopia: a community-based cross-sectional study. *PLoS One.* 2015;10(8):e0136342.

85. Alelign T, Degarege A, Erko B. Soil-transmitted helminth infections and associated risk factors among schoolchildren in Durbete town, northwestern Ethiopia. *Journal of parasitology research.* 2015;2015.

86. Alemayehu B, Tomass Z. Schistosoma mansoni infection prevalence and associated risk factors among schoolchildren in Demba Girara, Damot Woide District of Wolaita Zone, Southern Ethiopia. *Asian Pacific journal of tropical medicine.* 2015;8(6):457-463.

87. Jejaw A, Zemene E, Alemu Y, Mengistie Z. High prevalence of Schistosoma mansoni and other intestinal parasites among elementary school children in Southwest Ethiopia: a cross-sectional study. *BMC Public Health.* 2015;15(1):1-7.

88. Gashaw F, Aemero M, Legesse M, et al. Prevalence of intestinal helminth infection among school children in Maksegnit and Enfranz Towns, northwestern Ethiopia, with emphasis on Schistosoma mansoni infection. *Parasites & vectors.* 2015;8(1):1-8.

89. Boko PM, Ibikounle M, Onzo-Aboki A, et al. Schistosomiasis and soil transmitted helminths distribution in Benin: a baseline prevalence survey in 30 districts. *PLoS One.* 2016;11(9):e0162798.

90. Ortu G, Assoum M, Wittmann U, et al. The impact of an 8-year mass drug administration programme on prevalence, intensity and co-infections of soil-transmitted helminthiases in Burundi. *Parasites & vectors.* 2016;9(1):1-17.

91. Bopda J, Nana-Djeunga H, Tenaguem J, et al. Prevalence and intensity of human soil transmitted helminth infections in the Akonolinga health district (Centre Region, Cameroon): Are adult hosts contributing in the persistence of the transmission? *Parasite epidemiology and control.* 2016;1(2):199-204.

92. Alemu A, Tegegne Y, Damte D, Melku M. Schistosoma mansoni and soil-transmitted helminths among preschool-aged children in Chuahit, Dembia district, Northwest Ethiopia: prevalence, intensity of infection and associated risk factors. *BMC Public Health.* 2016;16(1):1-9.

93. Mekonnen Z, Suleman S, Biruksew A, Tefera T, Chelkeba L. Intestinal polyparasitism with special emphasis to soil-transmitted helminths among residents around Gilgel Gibe Dam, Southwest Ethiopia: a community based survey. *BMC Public Health.* 2016;16(1):1-7.

94. Ferreira FS, Baptista-Fernandes T, Oliveira D, et al. Giardia duodenalis and soil-transmitted helminths infections in children in São Tomé and Príncipe: do we think Giardia when addressing parasite control? *Journal of tropical pediatrics.* 2015;61(2):106-112.

95. Müller I, Yap P, Steinmann P, et al. Intestinal parasites, growth and physical fitness of schoolchildren in poor neighbourhoods of Port Elizabeth, South Africa: a cross-sectional survey. *Parasites & Vectors.* 2016;9(1):1-13.

96. Fuhrimann S, Winkler MS, Kabatereine NB, et al. Risk of intestinal parasitic infections in people with different exposures to wastewater and fecal sludge in Kampala, Uganda: a cross-sectional study. *PLoS neglected tropical diseases.* 2016;10(3):e0004469.

97. Sumbele IUN, Nkemnji GB, Kimbi HK. Soil-transmitted helminths and plasmodium falciparum malaria among individuals living in different agroecosystems in two rural communities in the mount Cameroon area: a cross-sectional study. *Infectious diseases of poverty.* 2017;6(1):1-15.

98. Inocencio da Luz R, Linsuke S, Lutumba P, Hasker E, Boelaert M. Assessment of schistosomiasis and soil‐transmitted helminths prevalence in school‐aged children and opportunities for integration of control in local health services in Kwilu Province, the Democratic Republic of the Congo. *Trop Med Int Health.* 2017;22(11):1442-1450.

99. Kabore A, Ibikounle M, Tougoue JJ, et al. Initiating NTD programs targeting schistosomiasis and soil-transmitted helminthiasis in two provinces of the Democratic Republic of the Congo: Establishment of baseline prevalence for mass drug administration. *Acta tropica.* 2017;166:177-185.

100. Tefera E, Belay T, Mekonnen SK, Zeynudin A, Belachew T. Prevalence and intensity of soil transmitted helminths among school children of Mendera Elementary School, Jimma, Southwest Ethiopia. *The Pan African Medical Journal.* 2017;27.

101. Shittu O, Shittu DS, Opeyemi OA, et al. Overlapping distribution of Plasmodium falciparum and soil transmitted helminths in a malaria hyper-endemic region, North-Central Nigeria. *Asian Paci J Tropi Dis.* 2017;7(12):930-935.

102. Adriko M, Tinkitina B, Arinaitwe M, Kabatereine NB, Nanyunja M, M. Tukahebwa E. Impact of a national deworming campaign on the prevalence of soil-transmitted helminthiasis in Uganda (2004-2016): implications for national control programs. *PLOS Neglected Tropical Diseases.* 2018;12(7):e0006520.

103. Ibikounlé M, Onzo-Aboki A, Doritchamou J, et al. Results of the first mapping of soil-transmitted helminths in Benin: Evidence of countrywide hookworm predominance. *PLoS neglected tropical diseases.* 2018;12(3):e0006241.

104. Coulibaly G, Ouattara M, Dongo K, et al. Epidemiology of intestinal parasite infections in three departments of south-central Côte d’Ivoire before the implementation of a cluster-randomised trial. *Parasite Epidemiology and Control.* 2018;3(2):63-76.

105. Ibrahim T, Zemene E, Asres Y, et al. Epidemiology of soil-transmitted helminths and Schistosoma mansoni: a base-line survey among school children, Ejaji, Ethiopia. *The Journal of Infection in Developing Countries.* 2018;12(12):1134-1141.

106. Adu-Gyasi D, Asante KP, Frempong MT, et al. Epidemiology of soil transmitted Helminth infections in the middle-belt of Ghana, Africa. *Parasite epidemiology and control.* 2018;3(3):e00071.

107. Olopade BO, Idowu CO, Oyelese AO, Aboderin AO. Intestinal parasites, nutritional status and cognitive function among primary school pupils in Ile-Ife, Osun State, Nigeria. *African Journal of Infectious Diseases.* 2018;12(2):21-28.

108. Babamale OA, Ugbomoiko US, Heukelbach J. High prevalence of Plasmodium falciparum and soil-transmitted helminth co-infections in a periurban community in Kwara State, Nigeria. *Journal of infection and public health.* 2018;11(1):48-53.

109. Bronzan RN, Dorkenoo AM, Agbo YM, et al. Impact of community-based integrated mass drug administration on schistosomiasis and soil-transmitted helminth prevalence in Togo. *PLoS neglected tropical diseases.* 2018;12(8):e0006551.

110. Tekalign E, Bajiro M, Ayana M, Tiruneh A, Belay T. Prevalence and intensity of soil-transmitted helminth infection among rural community of southwest Ethiopia: a community-based study. *BioMed Research International.* 2019;2019.

111. Weldesenbet H, Worku A, Shumbej T. Prevalence, infection intensity and associated factors of soil transmitted helminths among primary school children in Gurage zone, South Central Ethiopia: a cross-sectional study design. *BMC research notes.* 2019;12(1):1-6.

112. Ihejirika OC, Nwaorgu OC, Ebirim CI, Nwokeji CM. Effects of intestinal parasitic infections on nutritional status of primary children in Imo State Nigeria. *The Pan African Medical Journal.* 2019;33.

113. Bah YM, Bah MS, Paye J, et al. Soil-transmitted helminth infection in school age children in Sierra Leone after a decade of preventive chemotherapy interventions. *Infectious diseases of poverty.* 2019;8(04):31-40.

114. Ruth MMR, Cedric Y, Malla ME, et al. Intestinal Helminth Infections and Associated Risk Factors among School-Aged Children of Bamendjou Community, West Region of Cameroon. *Journal of Parasitology Research.* 2021;2021.

115. Fentahun A, Hailu T, Alemu G. Prevalence of Intestinal Parasites and Schistosoma mansoni and Associated Factors among Fishermen at Lake Tana, Northwest Ethiopia. *BioMed Research International.* 2021;2021.

116. Workineh L, Kiros T, Damtie S, Andualem T, Dessie B. Prevalence of soil-transmitted Helminth and Schistosoma mansoni infection and their associated factors among Hiruy Abaregawi primary school children, rural Debre Tabor, North West Ethiopia: A Cross-Sectional Study. *Journal of Parasitology Research.* 2020;2020.

117. Zeleke AJ, Bayih AG, Afework S, Gilleard JS. Treatment efficacy and re-infection rates of soil-transmitted helminths following mebendazole treatment in schoolchildren, Northwest Ethiopia. *Tropical Medicine and Health.* 2020;48(1):1-6.

118. Hailu Amare H, Lindtjørn B. Helminth infections among rural schoolchildren in Southern Ethiopia: A cross-sectional multilevel and zero-inflated regression model. *PLoS neglected tropical diseases.* 2020;14(12):e0008002.

119. Asfaw MA, Gezmu T, Wegayehu T, et al. Soil-transmitted helminth infections among pre-school aged children in Gamo Gofa zone, Southern Ethiopia: Prevalence, intensity and intervention status. *PLoS One.* 2020;15(12):e0243946.

120. Gebreyesus TD, Tadele T, Mekete K, et al. Prevalence, intensity, and correlates of schistosomiasis and soil-transmitted helminth infections after five rounds of preventive chemotherapy among school children in Southern Ethiopia. *Pathogens.* 2020;9(11):920.

121. Dejon-Agobé JC, Honkpehedji YJ, Zinsou JF, et al. Epidemiology of schistosomiasis and soil-transmitted helminth coinfections among schoolchildren living in Lambaréné, Gabon. *The American Journal of Tropical Medicine and Hygiene.* 2020;103(1):325.

122. Werunga DK, Omukunda EN, Korir JC. Prevalence and Intensity of Intestinal Helminth Infections in Preschool Pupils in Lugari Subcounty, Kakamega County, Kenya. *Journal of Parasitology Research.* 2020;2020.

123. Allan L, Mbai FN, Yole DS, Owino M. Intensity of nematode infection in children aged 3 to 5 Years living in mukuru kwa Njenga slum settlement, Nairobi, Kenya. *Journal of Tropical Medicine.* 2020;2020.

124. Chege N. The prevalence of intestinal parasites and associated risk factors in school-going children from informal settlements in Nakuru town, Kenya. *Malawi Medical Journal.* 2020;32(2):80-86.

125. Kabatende J, Mugisha M, Ntirenganya L, et al. Prevalence, intensity, and correlates of soil-transmitted helminth infections among school children after a decade of preventive chemotherapy in Western Rwanda. *Pathogens.* 2020;9(12):1076.

126. Midzi N, Montresor A, Mutsaka-Makuvaza MJ, et al. Elimination of STH morbidity in Zimbabwe: Results of 6 years of deworming intervention for school-age children. *PLoS neglected tropical diseases.* 2020;14(10):e0008739.

127. Ntonifor HN, Chewa JS, Oumar M, Mbouobda HD. Intestinal helminths as predictors of some malaria clinical outcomes and IL-1β levels in outpatients attending two public hospitals in Bamenda, North West Cameroon. *PLoS neglected tropical diseases.* 2021;15(3):e0009174.

128. Sumbele IUN, Otia OV, Bopda OSM, Ebai CB, Kimbi HK, Nkuo-Akenji T. Polyparasitism with Schistosoma haematobium, Plasmodium and soil-transmitted helminths in school-aged children in Muyuka–Cameroon following implementation of control measures: a cross sectional study. *Infectious diseases of poverty.* 2021;10(1):1-16.

129. Cedric Y, Nadia NAC, Payne VK, Sabi Bertrand M, Romeo NG. Gastrointestinal Nematodes among Residents in Melong, Moungo Division, Littoral Region, Cameroon. *BioMed Research International.* 2021;2021.

130. Ayele A, Tegegne Y, Derso A, Eshetu T, Zeleke AJ. Prevalence and associated factors of intestinal helminths among kindergarten children in Gondar town, northwest Ethiopia. *Pediatric Health, Medicine and Therapeutics.* 2021;12:35.

131. Ejigu K, Hailu T, Alemu M. Efficacy of Mebendazole and Praziquantel against Soil-Transmitted Helminths and Schistosoma mansoni Infections among Schoolchildren in Northwest Ethiopia. *BioMed Research International.* 2021;2021.

132. Andargie D, Tegegne Y, Worku L. Evaluation of Intestinal Parasite Infection in Low and High Coverage of Graduated Households, Northwest Ethiopia: A Comparative-Based Crosssectional Study. *Journal of parasitology research.* 2021;2021.

133. Abera D, Wordofa M, Mesfin A, et al. Intestinal helminthic infection and allergic disorders among school children enrolled in mass deworming program, Sululta, Ethiopia. *Allergy, Asthma & Clinical Immunology.* 2021;17(1):1-11.

134. Zeleke AJ, Derso A, Bayih AG, Gilleard JS, Eshetu T. Prevalence, Infection Intensity and Associated Factors of Soil-Transmitted Helminthiasis Among School-Aged Children from Selected Districts in Northwest Ethiopia. *Research and Reports in Tropical Medicine.* 2021;12:15.

135. Aribodor OB, Ekwunife CA, Sam-Wobo SO, et al. Status of Intestinal Helminth Infection in Schools Implementing the Home-Grown School Feeding Program and the Impact of the Program on Pupils in Anambra State, Nigeria. *Acta Parasitologica.* 2021;66(4):1528-1537.

136. Brandt O, Wegenstein B, Müller I, et al. Association between allergic sensitization and intestinal parasite infection in schoolchildren in Gqeberha, South Africa. *Clinical & Experimental Allergy.* 2022.

137. Bosch F, Palmeirim MS, Ali SM, Ame SM, Hattendorf J, Keiser J. Diagnosis of soil-transmitted helminths using the Kato-Katz technique: What is the influence of stirring, storage time and storage temperature on stool sample egg counts? *PLoS neglected tropical diseases.* 2021;15(1):e0009032.
