# Supplementary material for: Global prevalence of Ascaris infection in humans (2010–2021): a systematic review and meta-analysis
Source: Infect Dis Poverty. 2022 Nov 18;11:113. doi: 10.1186/s40249-022-01038-z (PMC9673379; doi:10.1186/s40249-022-01038-z)
Supplement: Supplementary file 3 — Additional file 3: Table S3. Prevalence estimates for Ascaris infection based on sub-groups according to different geographic and climate parameters, calculated using a random effects model. Table S4. Association between study variables and heterogeneity of Ascariasis prevalence estimates. [file 40249_2022_1038_MOESM3_ESM.docx]

**Table S3.** Prevalence estimates of *Ascaris* infection based on sub-groups according to different geographic and climate parameters, calculated using a random effects model.

| **Parameters/subgroups** | **Number of datasets** | **Number of people screened (total)** | **Number of test positive people** | **Pooled prevalence**  **% (95% *CI*)** | **Prevalence ratio**  **(95% *CI*)** |
| --- | --- | --- | --- | --- | --- |
| **Latitude** |  |  |  |  |  |
| 0–20° | 537 | 1,317,044 | 134,074 | 13.41 (12.34–14.51) | 31.17 (28.69–33.87) |
| 20–40° | 212 | 3,435,624 | 30,246 | 6.31 (5.66–7.01) | 2.69 (2.48–2.93) |
| 40–60° | 9 | 171,208 | 559 | 0.53 (0.17–1.04) | 1 |
| **Longitude** |  |  |  |  |  |
| 0–20° | 166 | 194,379 | 16,581 | 11.98 (9.92–14.21) | 22.04 (21.47–22.62) |
| 20–40° | 214 | 1,024,715 | 81,686 | 9.94 (8.57–11.41) | 20.59 (20.13–21.07) |
| 40–60° | 65 | 2,089,370 | 8,086 | 4.55 (3.54–5.69) | 1 |
| 60–80° | 86 | 407,087 | 13,361 | 16.72 (14.23–19.36) | 8.48 (8.25–8.71) |
| 80–100° | 76 | 61,819 | 10,617 | 15.87 (11.06–21.36) | 44.37 (43.16–45.62) |
| 100–120 | 98 | 1,011,222 | 17,264 | 11.65 (10.27–13.11) | 4.41 (4.29–4.52) |
| >120 | 53 | 135,284 | 17,284 | 6.51 (3.29–10.70) | 33.01 (32.17–33.87) |
| **Relative humidity (%)** |  |  |  |  |  |
| ≤ 40 | 38 | 1,939,743 | 2,825 | 5.61 (4.48–6.85) | 1 |
| 41–60 | 116 | 946,445 | 40,683 | 9.15 (7.64–10.79) | 29.51 (28.41–3065) |
| 60–79 | 464 | 1,905,113 | 93,868 | 10.02 (9.10–10.97) | 33.83 (32.59–35.11) |
| ≥ 80 | 140 | 132,575 | 27,503 | 18.60 (15.54–21.86) | 142.44 (137.08–148.01) |
| **Mean annual precipitation (mm)** |  |  |  |  |  |
| 0–50 | 107 | 2,839,856 | 18,450 | 4.72 (4.02–5.48) | 1 |
| 51–100 | 228 | 1,085,757 | 67,407 | 11.01 (9.62–12.47) | 9.55 (9.40–9.71) |
| 101–200 | 360 | 921,099 | 66,072 | 12.56 (11.14–14.05) | 11.04 (10.86–11.22) |
| >200 | 63 | 77,164 | 12,950 | 15.26 (11.04–20.01) | 25.83 (25.28–26.83) |
| **Mean temperature (°C)** |  |  |  |  |  |
| ≤ 8 | 12 | 30,291 | 456 | 5.19 (3.10–7.77) | 1.30 (1.18–1.43) |
| 9–13 | 21 | 526,731 | 6,080 | 0.92 (0.51–1.43) | 1 |
| 14–18 | 113 | 1,110,595 | 26,711 | 5.04 (4.03–6.15) | 2.08 (2.02–2.14) |
| 19–24 | 242 | 769,777 | 67,459 | 10.67 (9.33–12.10) | 7.59 (7.39–7.79) |
| 25–29 | 357 | 2,464,973 | 63,172 | 14.81 (13.24–16.46) | 2.22 (2.16–2.27) |
| ≥30 | 13 | 21,509 | 1,001 | 11.14 (3.08–23.23) | 4.03 (3.77–4.30) |

**Table S4.** Association between study variables and Ascariasis prevalence estimates

| Study variable | Univariate analyses | | |  | Multivariate analyses with significant predictors | | |
| --- | --- | --- | --- | --- | --- | --- | --- |
|  | Coefficient | 95% *CI* | *P* |  | Coefficient | 95% *CI* | *P* |
| Human Development Index | -12% | -21% to -2% | 0.012 |  | -0.05% | -0.21 to 0.10 | 0.495 |
| Gross national income | -0.00004% | –0.00006 to -0.00002 | <0.001 |  | -0.00004% | –0.00007 to -0.00002 | < 0.001 |
| Latitude | -0.002% | -0.004 to -0.002 | <0.001 |  | -0.001% | -0.002 to 0.0004 | 0.149 |
| Longitude | 0.0002% | -0.00008 to 0.0005 | 0.139 |  | 0.001% | 0.0006 to 0.001 | < 0.001 |
| Temperature | 0.006% | 0.004 to 0.008 | <0.001 |  | 0.004% | 0.001 to 0.006 | 0.001 |
| Humidity | 0.002% | 0.001 to 0.003 | <0.001 |  | 0.001% | 0.0003 to 0.002 | 0.013 |
| Precipitation | 0.0004% | 0.0002 to 0.0006 | <0.001 |  | 0.00004% | -0.0002 to 0.0002 | 0.744 |
| Year of start sampling | 0.001% | -0.001 to 0.004 | 0.280 |  | 0.005% | -0.0005 to 0.011 | 0.075 |
| Year of end sampling | -0.0006% | -0.003 to 0.002 | 0.709 |  | -0.007% | -0.014 to -0.0007 | 0.030 |
