# Supplementary material for: Global prevalence of Ascaris infection in humans (2010–2021): a systematic review and meta-analysis
Source: Infect Dis Poverty. 2022 Nov 18;11:113. doi: 10.1186/s40249-022-01038-z (PMC9673379; doi:10.1186/s40249-022-01038-z)
Supplement: Supplementary file 4 — Additional file 4: Figure S1. Search strategy in databases. Figure S2. Random-effects meta-regression analyses of the prevalence of Ascaris infection in general population according to (A) a country's income level, showing a statistically significant downward trend in prevalence in countries with higher income levels; (B) human development index (HDI), showing a statistically significant downward trend in prevalence in countries with higher HDIs. Figure S3. Random-effects meta-regression analyses of the prevalence of Ascaris infection in the general human population, according to publication year, showing a statistically non-significant upward trend in prevalence in recent years. Figure S4. Random-effects meta-regression analyses of the prevalence of Ascaris infection in the general human population, according to (A) geographical latitude, showing a statistically significant downward trend in prevalence with increasing geographical latitude; and (B) geographical longitude, showing a statistically non-significant upward trend in prevalence with increasing geographical longitude. Figure S5. Random-effects meta-regression analyses of the prevalence of Ascaris infection in the general human population, according to (A) the mean annual relative humidity, showing a statistically significant upward trend in prevalence with increasing humidity; and (B) the mean annual precipitation rate, showing a statistically significant upward trend in prevalence with increasing precipitation; (C), the mean annual temperature, showing a statistically significant upward trend in prevalence with increasing environmental temperature. [file 40249_2022_1038_MOESM4_ESM.docx]

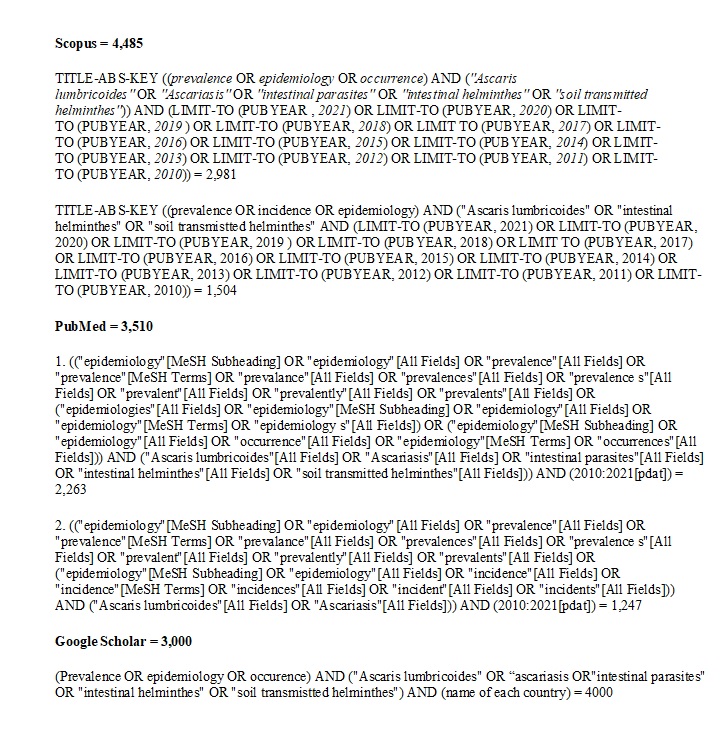


**Figure S1.** Search strategy in databases


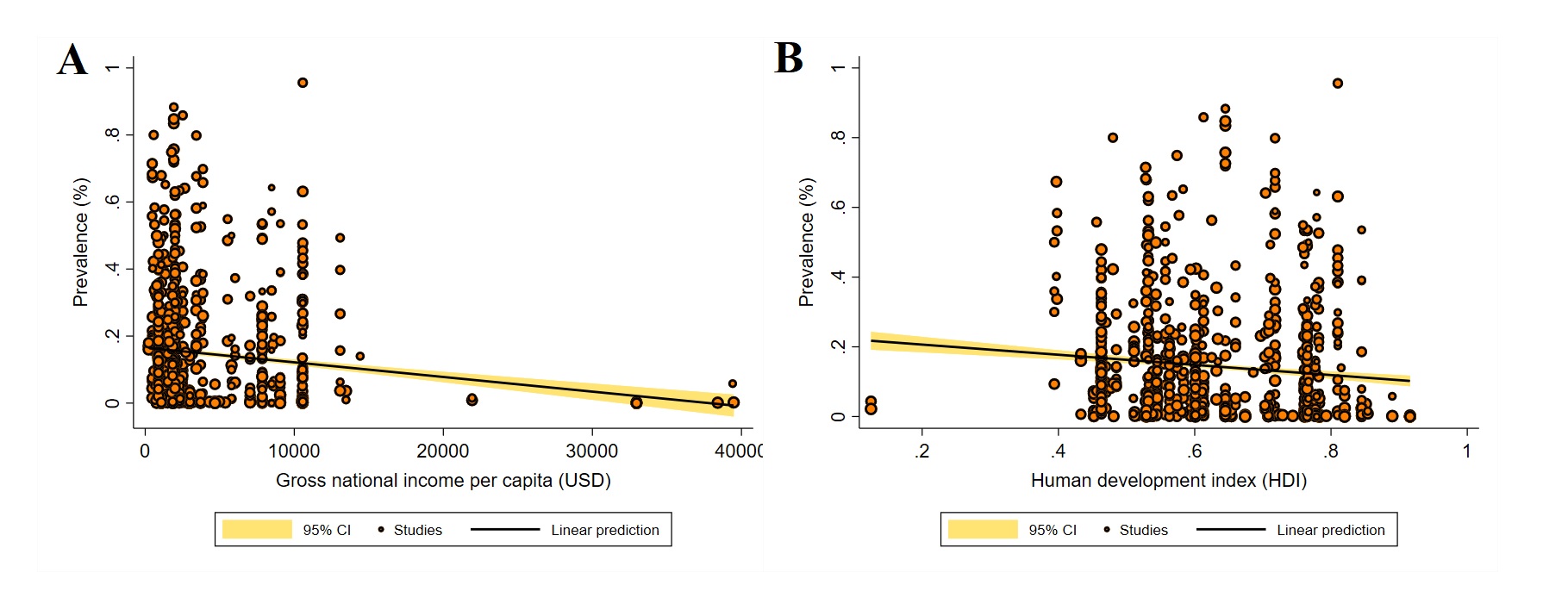


**Figure S2.** Random-effects meta-regression analyses of the prevalence of *Ascaris* infection in general population according to (A) a country's income level, showing a statistically significant downward trend in prevalence in countries with higher income levels; (B) human development index (HDI), showing a statistically significant downward trend in prevalence in countries with higher HDIs.


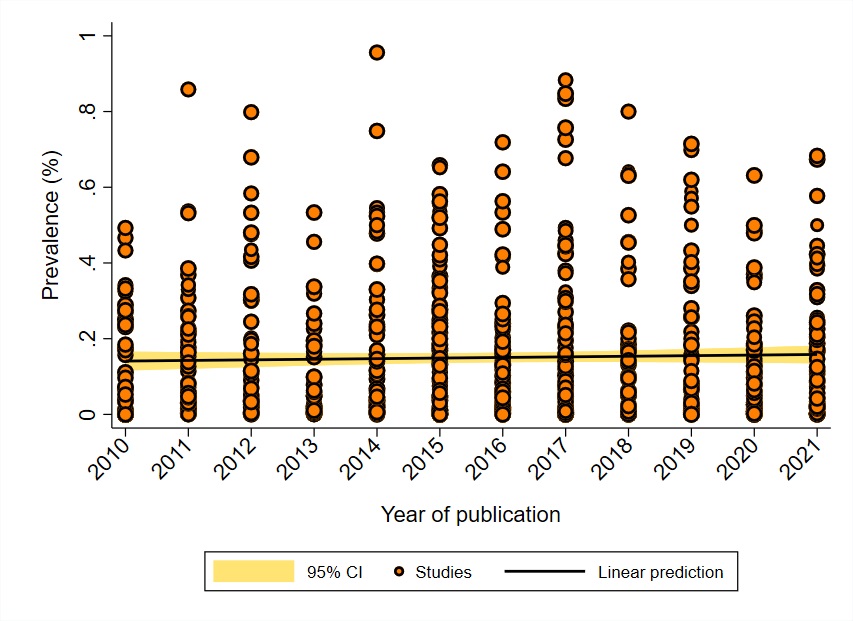


**Figure S3.** Random-effects meta-regression analyses of the prevalence of *Ascaris* infection in general population according to publication year, showing a statistically non-significant upward trend in prevalence in recent years.


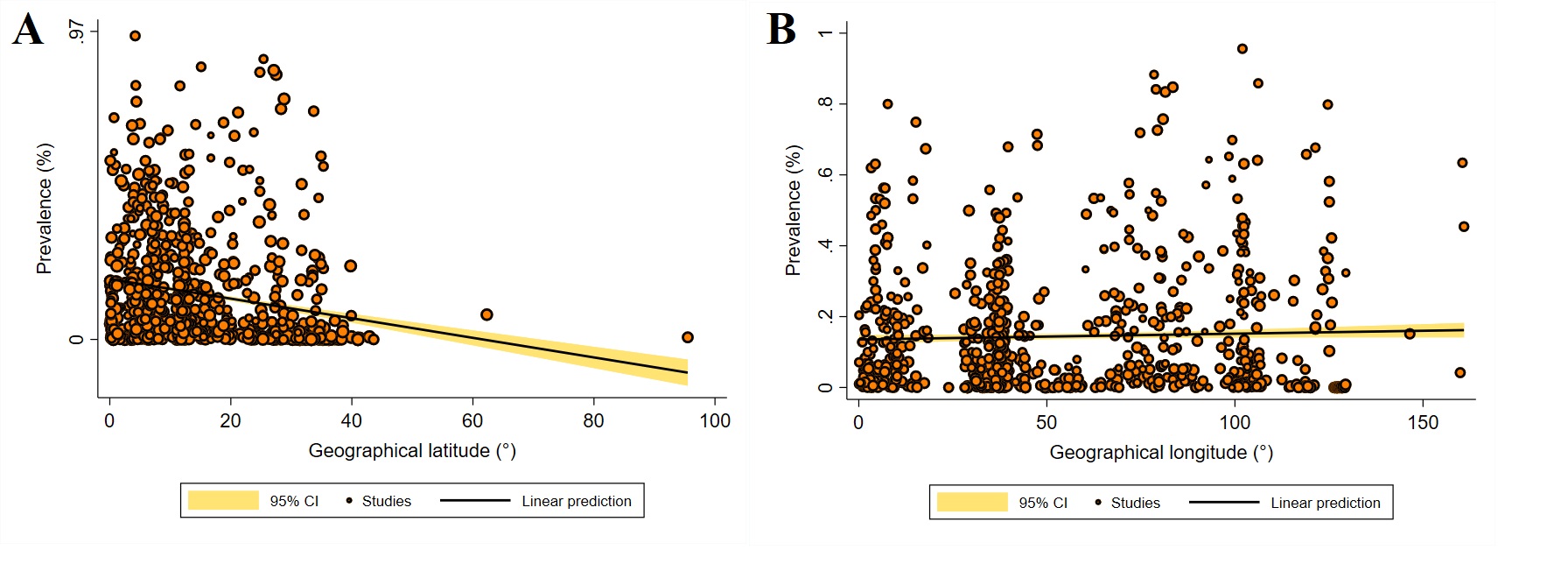


**Figure S4.** Random-effects meta-regression analyses of the prevalence of *Ascaris* infection in general population according to (A) geographical latitude, showing a statistically significant downward trend in prevalence with increasing geographical latitude; and (B) geographical longitude, showing a statistically non-significant upward trend in prevalence with increasing geographical longitude.


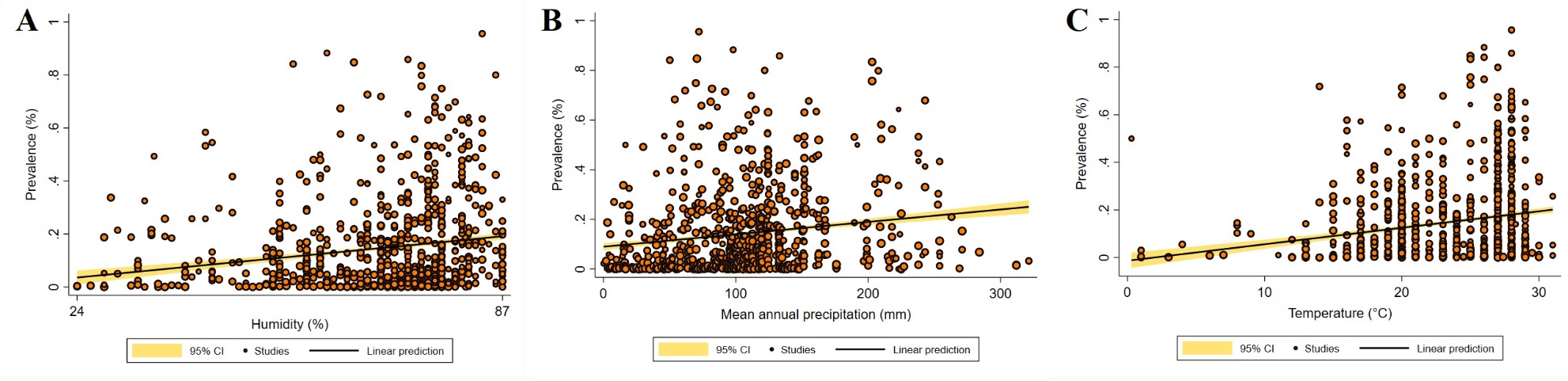


**Figure S5.** Random-effects meta-regression analyses of the prevalence of *Ascaris* infection in general population according to (A) the mean annual relative humidity, showing a statistically significant upward trend in prevalence with increasing humidity; and (B) the mean annual precipitation rate, showing a statistically significant upward trend in prevalence with increasing precipitation; (C), the mean annual temperature, showing a statistically significant upward trend in prevalence with increasing environmental temperature.
